# First Order Expansion in the Semiclassical Limit of the Levy–Lieb Functional

**DOI:** 10.1007/s00205-025-02143-7

**Published:** 2025-11-07

**Authors:** Maria Colombo, Simone Di Marino, Federico Stra

**Affiliations:** 1 EPFL, AMCV, Lausanne, Switzerland; 2 Università di Genova (DIMA), MaLGa, Genova, Italy; 3 Politecnico di Torino (DISMA), Torino, Italy

**Keywords:** 81Q05, 82C70, 49Q20

## Abstract

We prove the conjectured first order expansion of the Levy–Lieb functional in the semiclassical limit, arising from Density Functional Theory (DFT). In particular, we prove a general asymptotic first order lower bound in terms of the zero point oscillation functional and the corresponding asymptotic upper bound in the case of two electrons in one dimension. This is accomplished by interpreting the problem as the singular perturbation of an Optimal Transport problem via a Dirichlet penalization.

## Introduction

A revolutionary approach to finding the ground state energy of a many-body electron system was developed in the 19s by Hohenberg and Kohn [24]: The Density Functional Theory (DFT). Their idea, subsequently formalized mathematically by Lieb and Levy [30], can be seen as breaking up the minimization over all wave functions into a first minimization over those wavefunctions having a given one-particle density and then minimizing the resulting function over the one-particle density. In particular we define that

1.1 $$\begin{aligned} F^\varepsilon (\rho ) = \inf \left\{ { \int _{\mathbb {R}^{dN}} \left( \frac{\varepsilon }{2} |{\nabla \psi }|^2 + V_{\textrm{ee}} |{\psi }|^2 \right) \textrm{d}x_1 \dots \textrm{d}x_N}\right\} {\psi \in H^1(\mathbb {R}^{dN}),\ \psi \mapsto \rho }, \end{aligned}$$

where $\psi \mapsto \rho$ means that the one electron density of $\psi$ is $\rho$, that is, for all $i=1, \ldots , N$, we require $\rho (x_i)= \iint | \psi |^2(x_1, \ldots , x_n) \, dx_1 \, \ldots \, \widehat{ d x_i } \, \ldots d x_N$, and, moreover,

$$\begin{aligned} V_{\textrm{ext}}(x_1,\dots ,x_n) = \sum _{i<j} \frac{1}{|{x_i-x_j}|}. \end{aligned}$$

Then, for any external potential $V_{ext}$ the corresponding energy of the ground state will be equal to

$$\begin{aligned} E(V_{\textrm{ext}}) = \inf \left\{ { F^{\hbar ^2}(\rho ) + \int _{\mathbb {R}^d} V_{\textrm{ext}} \textrm{d}\rho }\right\} {\rho \in \mathscr {M}_+(\mathbb {R}^d),\ \rho (\mathbb {R}^d)=N}. \end{aligned}$$

In particular, $F^{\hbar ^2}(\rho )$ is a *universal functional* in the sense that depends only on the number of electrons, whereas the dependence on the external field appears only in the term $\int V_\textrm{ext}\textrm{d}\rho$ in the outer minimization. It becomes then fundamental for applications to approximate $F^\hbar (\rho )$ and compute this value efficiently. In recent years a new approach gained importance, relying on the analysis of the Strictly Correlated Electrons (SCE) case. The interest in this approach resides in two main reasons: it is mathematically rigorous since it is a limiting procedure starting from the exact functional, and it is highly non-local in nature, thus it can be thought as complementary in some sense to the more classical Local Density Approximation (LDA) approach [27] (see also [28] for other universal approximating functionals). The idea is to have a parameter $\beta >0$ which tunes the strength of the interaction between the electrons and the functional

$$\begin{aligned} F^{\hbar ^2}_\beta (\rho ) = \inf \left\{ { \int _{\mathbb {R}^{dN}} \left( \frac{\hbar ^2}{2} |{\nabla \psi }|^2 + \beta V_{\textrm{ee}} |{\psi }|^2 \right) \textrm{d}x_1 \dots \textrm{d}x_N}\right\} {\psi \in H^1(\mathbb {R}^{dN}),\ \psi \mapsto \rho }. \end{aligned}$$

The SCE limit considers the case $\beta \rightarrow \infty$, the asymptotic expansion of $F^{\hbar ^2}_{\beta } (\rho )$ in $\beta$, to then deduce information about $\beta <\infty$. This limiting procedure has more equivalent formulations. Using the homogeneity of $V_{ee}$, $F^{\hbar ^2}_{\beta }$ is computed from $F^{\hbar ^2}$ by scaling, namely $F^{\hbar ^2}_{\beta }(\rho )= \beta ^2 F^{\hbar ^2} (\rho _\beta )$ where $\rho _{\beta }(x)= \beta ^{-d} \rho (x/\beta )$. Another equivalent approach considers a varying kinetic energy coefficient: since $F^{\hbar ^2}_{\beta } (\rho ) = \beta F^{\hbar ^2/\beta }(\rho )$, the asymptotic expansion of $F^{\hbar ^2}_{\beta }$ as $\beta \rightarrow +\infty$ can be studied by means of the asymptotic expansion of $F^{\varepsilon }(\rho )$ as $\varepsilon \rightarrow 0$, which is mathematically convenient since the zeroth-order term of the expansion does not need to be renormalized. This is formally the same as changing the value of $\hbar$ in the original definition of the Levy–Lieb functional, but the rigorous justification of this non-physical procedure (since $\hbar$ is a physical constant) relies on the previous explanation.

Seidl was the first to conjecture the SCE limit case for $\varepsilon \rightarrow 0$ in [39] and further investigated in [23]. This new functional found its use, for example in [6, 32, 33, 43] it states that

$$\begin{aligned} \lim _{\varepsilon \rightarrow 0} F^{\varepsilon }(\rho ) = F_{OT}(\rho ) := \inf \left\{ \int _{ \mathbb {R}^{3N} } V_{\textrm{ee}} \, d \gamma \; : \; \gamma \in \Pi _N(\rho ) \right\} , \end{aligned}$$

where $\Pi _N(\rho )$ is the set of probabilities in $\mathbb {R}^{dN}$ which induce $\rho$, namely such that its push-forward through any projection $e_i: (x_1, \ldots , x_N) \mapsto x_i$ is $\rho$. We denote with $\Pi _0(\rho )$ the set of $\gamma \in \Pi _N(\rho )$ which are minimizers for $F_{OT}(\rho )$. Moreover, in the physics literature, ansatz of minimizers in the 1D case and radial case were conjectured in [39, 40]: in the 1D case the conjecture was proven to hold in [9], while in the radial case various counterexamples were found in [3, 11, 38]. The functional $F_{OT}$ has been studied in the last years also with regard to the limit of infinitely many particles: the zeroth order expansion for $N \rightarrow \infty$ was investigated in [15], proving the mean field limit, while the first order was proven independently in [26] and [16, 17], with ideas coming from the seminal papers [36, 37], and in connection with the Lieb-Oxford inequality [29, 31]. We refer the reader also to the recent review [20].

Already in [39], the next order in the asymptotic expansion of $F^\varepsilon$ for $\varepsilon \rightarrow 0$ was conjectured in some cases, while the most general formulation appears in [22]:

1.2 $$\begin{aligned} F^{\varepsilon }(\rho ) = F_{OT}(\rho ) + \sqrt{ \varepsilon } F_{ZPO}(\rho ) + O(\varepsilon ^{3/4} ). \end{aligned}$$

In both [22, 39] there is an explicit conjecture for the *zero point oscillation* functional $F_{ZPO}(\rho )$ in the case $d=1$, involving the eigenvalues of the Hessian of an effective potential described in the following. As in [8], we introduce the potential *u* which solves the dual problem for $F_{OT}$; its existence and regularity have been studied in [7, 10, 18, 21]. This is known to be unique if the support of $\rho$ is connected, *u* is Lipschitz, $F_{OT}(\rho )= N\int u \textrm{d}\rho$,

1.3 $$\begin{aligned} V(x_1, \ldots , x_N) = V_{ee}(x_1, \ldots , x_N) - u(x_1) - \ldots - u(x_N) \ge 0 \quad \forall x_1, \ldots , x_N \in \mathbb {R}^d, \end{aligned}$$

and the optimal plans are supported in the set $\{V=0\}$. In particular, $D^2V$ is well defined $\gamma$-almost everywhere for every optimal plan $\gamma$ and so we can define that

$$\begin{aligned} F_{ZPO}(\rho )= \inf \left\{ { \frac{1}{2}\int _{\mathbb {R}^{dN}}{{\,\textrm{tr}\,}}\left( \sqrt{D^2V}\right) \textrm{d}\gamma }\right\} { \gamma \in \Pi _0 (\rho )}. \end{aligned}$$

In the physics literature *V* is called the effective potential; *u* is the Lagrange multiplier associated with the constraint on the marginals, thought as a confining potential which keeps the optimal plan concentrated on minimal energy configurations with respect to the effective potential; $\frac{1}{2} {{\,\textrm{tr}\,}}\left( \sqrt{D^2V}\right)$ is the lowest eigenvalue of the harmonic oscillator associated with the quadratic form given by $D^2V$.

From the mathematical viewpoint, the 0-th order approximation was obtained in [13] for the bosonic case and for the fermionic case only when $N=2$. Then it was settled in [2] for $N=3$ with the help of spins; in [25] with a different approach involving mixed states (that is, considering the convex relaxation $\bar{F}_{\varepsilon }(\rho )$ of $F_{\varepsilon }(\rho )$); finally, [14] provided a proof using only pure states for every *N*.

Regarding the 1st order, the best known bound in [25] proves that $\bar{F}_{\varepsilon } (\rho ) \le F_{OT}(\rho ) + \sqrt{\varepsilon } C_{\rho }$ for every admissible $\rho$, namely an upper bound on the conjectured order of the next term. Our main contribution can be summarised as follows:

### Theorem 1.1

Let $N\ge 2$ and $d\ge 1$ be fixed integers. Let $\rho \in \mathcal {P}(\mathbb {R}^d)$ be such that $F^1(\rho ) < \infty$, *u* the optimal Kantorovich potential associated to the problem $F_{OT}(\rho )$ and suppose that *V* as in (1.3) is such that $V \in C^2_{loc} ( \{ V=0 \})$. Then

- we always have
  1.4 $$\begin{aligned} \liminf _{\varepsilon \rightarrow 0} \frac{ F^{\varepsilon }( \rho ) - F_{OT}(\rho ) }{\sqrt{\varepsilon }} \ge F_{ZPO} (\rho ); \end{aligned}$$
- if in addition $N=2$, $d=1$ and $\rho \in C^1(\mathbb {R})$ is a positive probability density, we also have
  1.5 $$\begin{aligned} \limsup _{\varepsilon \rightarrow 0} \frac{ F^{\varepsilon }( \rho ) - F_{OT}(\rho ) }{\sqrt{\varepsilon }} \le F_{ZPO} (\rho ). \end{aligned}$$

A few comments on the previous statement are listed below. Firstly, the regularity condition on *V* is natural when computing linear expansions and is automatically guaranteed under assumptions on $\rho$, namely the finiteness of its kinetic energy and a condition on its decay, see the observation following Theorem 1.3. Secondly, the restriction to $N=2$ in the upper bound (1.5) is just used to simplify technical details and not make the notation heavier, the general case $N\ge 2$ will be treated in a forthcoming note. In turn, a different strategy would be needed in dimension $d \ge 2$. In fact we rely heavily on the one dimensional setting for the existence, regularity and structure of optimal maps; even the existence of optimal maps is an open problem if $d \ge 2$ and $N \ge 3$. Thirdly, in $d\ge 2$, one may wish to study the problem with the addition of the constraint of the antisymmetry on $\psi$ (instead in $d=1$ it is sufficient to consider carefully the signs): however we believe that, as in [25], this issue is just of technical nature and can be circumvented considering mixed states.

We now describe the approach to Theorem 1.1 and provide in Theorems 1.2 and 1.3 below two strengthened statements, which easily imply respectively the lower bound (1.4) and the upper bound (1.5). Our approach is based on the variational formulation for the quantity $\frac{ F^{\varepsilon }(\rho ) - F_{OT}(\rho )}{\sqrt{\varepsilon }}$. Using that $F_{OT}(\rho )=N\int u \, d \rho =\int _{\mathbb {R}^{dN}} (u(x_1) + \ldots + u(x_N) ) | \psi |^2(x_1, \ldots , x_N) \,d x$ for every $\psi \mapsto \rho$, we obtain that

1.6 $$\begin{aligned} \frac{ F^{\varepsilon }(\rho ) - F_{OT}(\rho )}{\sqrt{\varepsilon }}&= \min \left\{ { E_{\varepsilon }(\psi )}\right\} {\psi \mapsto \rho }, \end{aligned}$$

1.7 $$\begin{aligned} E_{\varepsilon }(\psi )&= \int _{ \mathbb {R}^{dN} }\varepsilon ^{1/2} \frac{ |\nabla \psi (x)|^2}{2} + \varepsilon ^{-1/2} V(x) | \psi (x)|^2 \textrm{d}x. \end{aligned}$$

The core of Theorem 1.1 relies in a precise convergence result on $E_\varepsilon$. Firstly we study the convergence when we *drop the marginals constraint*, which simplifies our analysis but is not the one of physical interest, and we show, roughly speaking, that

1.8 $$\begin{aligned} E_{\varepsilon }(\psi ) {\mathop { \longrightarrow }\limits ^{\Gamma }} {\left\{ \begin{array}{ll} \displaystyle \frac{1}{2}\int _{\mathbb {R}^{dN}}{{\,\textrm{tr}\,}}\left( \sqrt{D^2V}\right) \textrm{d}\gamma \quad &  \text { if}\, \gamma ( \{ V>0 \}) =0\\ +\infty &  \text { otherwise.} \end{array}\right. } \end{aligned}$$

The notion of convergence in (1.8) is the so called $\Gamma$-convergence, the right variational tool to take the limit in sequences of minimizers, as it happens in Theorem 1.1. For brevity, we don’t introduce this abstract notion here but we just present its outcomes in Theorem 1.2. For $\mu _n, \mu \in \mathscr {M}(\mathbb {R}^d)$, we denote with $\mu _n \rightharpoonup \mu$ the weak-* convergence in duality with $C_0(\mathbb {R}^d)$ functions (continuous functions tending to zero at infinity).

### Theorem 1.2

($\Gamma$-convergence with free maginals) Let $V \in C^0(\mathbb {R}^d)$ with $V \ge 0$ and *V* locally $C^2$ in a neighborhood of $\{V=0\}$. Then we have

- for every sequence $\psi _{\varepsilon } \in H^1(\mathbb {R}^d)$ with $\int | \psi _{\varepsilon }|^2=1$ and $\liminf E_{\varepsilon } (\psi _{\varepsilon }) < \infty$, there exists $\gamma \in \mathscr {M}(\mathbb {R}^d)$ with $\gamma (\{ V>0 \})=0$ and $\gamma (\mathbb {R}^d) \le 1$ such that $| \psi _{\varepsilon }|^2 \rightharpoonup \gamma$ up to subsequences and
  $$\begin{aligned} \liminf _{\varepsilon \rightarrow 0} E_{\varepsilon }(\psi _{\varepsilon }) \ge \frac{1}{2}\int _{\mathbb {R}^{dN}}{{\,\textrm{tr}\,}}\left( \sqrt{D^2V}\right) \textrm{d}\gamma ; \end{aligned}$$
- for every $\gamma \in \mathcal {P}(\mathbb {R}^d)$ with $\gamma (\{ V>0 \})=0$, there exists a *recovery sequence* $\psi _{\varepsilon } \in H^1(\mathbb {R}^d)$ with $\int | \psi _{\varepsilon }|^2=1$ such that $| \psi _{\varepsilon }|^2 \rightharpoonup \gamma$ and
  $$\begin{aligned} \limsup _{\varepsilon \rightarrow 0} E_{\varepsilon }(\psi _{\varepsilon }) \le \frac{1}{2}\int _{\mathbb {R}^{dN}}{{\,\textrm{tr}\,}}\left( \sqrt{D^2V}\right) \textrm{d}\gamma . \end{aligned}$$

In order to deal with the actual first order expansion of $F^{\varepsilon }(\rho )$ we have to enforce the marginal constraint. For the lower bound we can use Theorem 1.2 (a), since the weak-* convergence preserves marginal constraints. In order to prove the upper bound we need to construct a recovery sequence $\bar{\psi }_{\varepsilon }$ with an almost optimal energy, but with the additional constraint on the marginals. This is in general nontrivial, since the Lavrentiev gap phenomenon could appear as happens for instance in an example of [14], where the constrained limit energy is smaller than the unconstrained one. This result is established in Theorem 1.3.

### Theorem 1.3

Let $\rho \in C^1(\mathbb {R})$ be a strictly positive probability density with finite kinetic energy

1.9 $$\begin{aligned} {{{\,\textrm{KE}\,}}(\rho ) = \frac{1}{2} \int _\mathbb {R}\left|{\partial _x\sqrt{\rho (x)}}\right|^2 \textrm{d}x = \int _\mathbb {R}\frac{|{\partial _x\rho (x)}|^2}{8\rho (x)}\textrm{d}x,} \end{aligned}$$

let $\gamma$ be the optimal transport plan for the problem with Coulomb cost and marginals $\rho$, let *u* be its Kantorovich potential, and assume that $u'' \in L^\infty (\mathbb {R})$. Let $V(x,y) = |x-y|^{-1} - u(x) - u(y)$.

Then there exists a recovery sequence $\psi _\varepsilon \in H^1(\mathbb {R}^2)$ such that $\int |{\psi _\varepsilon }|^2=1$, $|{\psi _\varepsilon }|^2\rightharpoonup \gamma$, $|{\psi _\varepsilon }|^2$ has the same marginals as $\gamma$ and

$$\begin{aligned} \limsup _{\varepsilon \rightarrow 0} E_\varepsilon (\psi _\varepsilon )= &   \limsup _{\varepsilon \rightarrow 0} \int _{\mathbb {R}^2} \biggl ( \varepsilon ^{1/2} \frac{|{\nabla \psi _\varepsilon }|^2}{2} + \varepsilon ^{-1/2} V |{\psi _\varepsilon }|^2 \biggr ) \textrm{d}x\\\le &   \frac{1}{2} \int _{\mathbb {R}^2} {{\,\textrm{tr}\,}}\bigl (\sqrt{D^2V}\bigr ) \textrm{d}\gamma . \end{aligned}$$

Theorem 1.3 is stated with a rather implicit assumption, namely that $u'' \in L^\infty (\mathbb {R})$. This is essentially equivalent to the condition (1.10) on the growth of the tails of $\rho$. For instance, we show in Lemma 4.1 that Theorem 1.3 can be applied to any strictly positive $C^1$ probability density $\rho$ such that ${{\,\textrm{KE}\,}}(\rho )<\infty$ and

1.10 $$\begin{aligned} \liminf _{|{x}|\rightarrow \infty }{} |{x}|^3 \rho (x) > 0. \end{aligned}$$

It would be interesting to prove the ZPO conjectured asymptotics for every $\rho$ and in any dimension, in particular removing the heavy tails assumption (1.10) since one expects physical densities to have exponential tails.

**Singular perturbations of optimal transport.** Since, without loss of generality, we can assume that $\psi >0$, we rewrite the functional $F^{\varepsilon }(\rho )$ in terms of $\gamma = | \psi |^2$ as

$$\begin{aligned} F^{\varepsilon }(\rho ) = \inf \left\{ \int _{\mathbb {R}^{dN}} V_{ee} (x_1, \ldots , x_n) \, d \gamma + \varepsilon \int _{\mathbb {R}^{dN}} \frac{ |\nabla \gamma |^2}{\gamma } \, d x \, : \; \gamma \in \Pi _N(\rho ) \right\} . \end{aligned}$$

We think $F^{\varepsilon }$ as a penalized multi-marginal optimal transport problem, where the penalization on the plan $\gamma$ is the Fisher information with respect to the Lebesgue measure $I(\gamma )=\int \frac{ |\nabla \gamma |^2}{\gamma } \, d x$. The ZPO conjecture is about the asymptotic expansion of this penalized OT problem. Other results in a similar spirit regard only the case $N=2$ with the entropic penalization $Ent(\gamma )= \int \gamma \ln (\gamma ) \, dx$ and either the Monge cost ( [19], only in 1D), or the quadratic distance ( [12] in a general Riemannian manifold) or a smooth convex cost ( [35], in $\mathbb {R}^d$). However, in [12] and in [35] the peculiar structure of the problem and an additivity property of the entropy are heavily used. The approach closest to ours is [19], but the construction of the recovery sequence is heavily simplified by taking advantage of working in the region $\{ T = \textrm{id}\}$. To our knowledge, the present result is the first next-order asymptotics with marginal constraint proven with a rather general strategy.

Theorem 1.2 works in a rather general setting and for any nonnegative potential which is locally $C^2$ around its zero set (we recall that here $\rho$ does not play a role). Its proof is also short and flexible, sharing analogies with other previous results in the literature. For instance, similar studies look at the spectrum of the operator $- \Delta + \varepsilon ^{-1} V$ as $\varepsilon \rightarrow 0$ (see for example [41, 42] and subsequent literature), but our point of view is slightly different. Instead of focusing on the behavior of the eigenvalues we want to know what is the minimial asymptotic energy given the asymptotic behavior of the wavefunction (that is, their weak limit $\gamma$). This is in view of applying the result to a generic $\gamma \in \Pi _N(\rho )$ which might not come from a sequence of eigenvectors.

Theorem 1.2 builds on the idea that, to have the correct minimal energy, the wavefunction must localize around $\{V=0\}$ and behave locally like a gaussian with variance depending on $D^2V$. Moreover this minimal energy can be achieved by a recovery sequence built superposing these gaussians, which will be elongated along the zero set of *V*. In the proof of Theorem 1.2 we construct a wavefunction for $\varepsilon >0$ which has the proven almost correct energy and therefore can be of interest also for numerical purposes.

**Ansatz on wavefunctions with asymptotically minimal energy.** The ansatz of a recovery sequence obtained as a superposition of Gaussians is already present in [22], but then, instead of superposing elongated Gaussians, a local nonlinear change of variables is considered, as well as Gaussians in the variable perpendicuar to the manifold $\{V=0\}$. In our opinion, there are no guarantees on the smallness of the kinetic energy in the direction tangent to the manifold. In [22] the constraint on the marginals is also diuscussed via the introduction of a Lagrange multiplier. However, the latter has no reason to exist for $\varepsilon >0$ and it is not clear the claim that it intervenes in the asymptotic expansion only at higher order terms.

**Outline of the paper.** The main idea in Theorem 1.2, once we show that every finite energy sequence needs to concentrate on the set $\{V=0\}$, is to analyze the energy locally around the zero set of *V*: being at a minimum, the first non-zero term in the Taylor expansion of *V* is the quadratic form corresponding to the second derivative. But then the energy is analogous to that of an anisotropic harmonic oscillator, of which we know the ground state (a Gaussian with proper covariance) and ground energy (see Lemma 2.1 and Lemma 2.2). On one hand, an approximation of *V* with its Taylor expansion will provide the lower bound, while in order to get an upper bound, we use Gaussians elongated along the zero set of *V*, and then we superpose them to obtain

1.11 $$\begin{aligned} f(x) = \int f_{\varepsilon ,x'}(x-x') \textrm{d}\gamma (x'), \end{aligned}$$

where $f_{\varepsilon ,x'}(x)$ is a suitable truncation of the Gaussian $C_{\varepsilon ,x'} \exp \left( -\varepsilon ^{-1/2}x^t\right. \left. {\sqrt{\nabla ^2V(x')}}{} x\right)$. The convexity of $E_{\varepsilon }$ when written in terms of $\gamma = | \psi |^2$ lets us conclude the upper bound with no marginal constraint. Along our recovery sequence the contribution of the two competing functionals, namely the rescaled kinetic and potential energies, is asymptotically equal. This equipartition of energy is expected since, in the case of convergence to a single $\delta _0$ and *V* quadratic, the kinetic energy rescales as ${{\,\textrm{KE}\,}}(\gamma _\lambda )=\lambda ^2{{\,\textrm{KE}\,}}(\gamma )$ and the potential energy as $\int V\textrm{d}\gamma _\lambda =\lambda ^{-2}\int V\textrm{d}\gamma$, where $\gamma (x)=\lambda \gamma (\lambda x)$.

To tackle Theorem 1.3, namely to modify our ansatz (1.11) to keep the marginals constraint, we split the total mass in two components: a main portion of the mass and a remaining mass. The main part consists of most of the mass and must have the correct asymptotic energy. In Sect.  4 we prove that a suitable superposition of truncated Gaussians (introduced in Sect.  3), similar to the one adopted for Theorem 1.2, produces a sequence with asymptotically optimal energy. The superposition is carefully chosen so as to leave a remaining mass with good properties for the subsequent construction.

The remaining mass is used to correct the marginals (see Sect.  5) with asymptotically vanishing cost (see Sect.  6). In the literature general methods for re-instating the marginals have been proposed, but they usually miss the correct order of magnitude for the kinetic energy or the potential energy: the only construction that controls in a fine way both of them has been proposed by [1], and then used then by [25] to retrieve in fact an upper bound with the correct order of magnitude. However the problem of this approximation is that it does not catch the correct local behavior because it is a superposition of gaussians with a fixed variance throughout the space. The latter issue motivates our choice of applying the deconvolution procedure in an unconventional way, namely only to the remaining mass, to connect the remaining marginals. Here we use the construction by Bindini in [1] since we are not interested in the sharp local energy but only the correct order of magnitude: in this way we have better control on the kinetic energy, which is bounded by the kinetic energy of the remaining marginals. This construction is presented in Sect.  5, where we show that this deconvolution procedure does not degrade too much the energy estimates. In particular, we estimate the potential energy energy of the deconvolved plan in terms the potential energy of the original plan connecting the remaining marginals up to the correct order in $\varepsilon$. This section unveals the most delicate issue of the paper, needed to meet the assumption of the deconvolution. In Sect.  6 we prove that there exists $\tilde{\gamma }_H$ such that $\int V \, d \tilde{\gamma } = o( \sqrt{\varepsilon })$.

This last estimate of the potential energy means that the remaining marginals $\rho -\rho ^1_\varepsilon$ and $\rho -\rho ^2_\varepsilon$ can be connected by a plan which is concentrated near $\{V=0\}={{\,\textrm{graph}\,}}T$. To prove it, we show that $T_\#(\rho -\rho ^1_\varepsilon )$ is close to $\rho -\rho ^2_\varepsilon$ in the Wasserstein sense. The marginals are obtained by convolution with very anisotropic kernels with size $\varepsilon ^{1/4}$ by $\beta ^{1/2}$, which is much larger. A naive argument about balance of mass can show that the Wasserstein distance is at most $\beta ^{1/2}$, but this is not sufficient. To show the desired estimate, we therefore need to employ a delicate analysis based on the Benamou-Brenier estimate of the transport cost, the linearization of the map *T* and a technical Proposition 6.3 to take advantage of the special structure of the projections of our truncated Gaussian kernels, introduced in Sect.  3.

## $\Gamma$-limit of the unconstrained problem

In this section we prove Theorem 1.2. The strategy is to prove that for a finite energy sequence, the measure $| \psi _{\varepsilon }|^2 \,d x$ will concentrate around $\{ V=0\}$, and here the potential term can be well approximated by its second order Taylor expansion. More precisely, at each minimum point *V* will have some degenerate directions, and some other nondegenerate directions along which it is bounded from below by a suitable quadratic form.

We can thus look at the linearized problem: using the explicit solution (a gaussian with known variance) when *V* is a positive definite quadratic form, we estimate locally from below the energy around minimal points (Lemma 2.1), proving in particular Theorem 1.2(a) for *V* a nondegenerate quadratic potential. In Lemma 2.2 for any semidefinite quadratic form we build a sequence that attains the minimum energy in the limit, while concentrating the mass at a point. This proves Theorem 1.2(b) for $\gamma =\delta _0$ and the construction is inspired by the equality cases in Lemma 2.1, but needs to deal with the possible degeneracy of *V* at 0 and with a localization, since we have no information on the potential outside the origin.

### Lemma 2.1

Let *A* be a positive definite symmetric matrix such that $\lambda Id \le A \le \Lambda Id$ for some $\Lambda> \lambda > 0$. Whenever $B_{2r}(0) \subset \Omega \subseteq \mathbb {R}^n$, there exist a constant *C* depending only on $\lambda$ and *r*, such that if $\sqrt{\varepsilon } \le \lambda ^2 r^2/n\Lambda$, we have

2.1 $$\begin{aligned} \int _{\Omega } \Bigl ( \varepsilon |\nabla \psi |^2 + | Ax|^2 | \psi |^2 \Bigr ) \, dx \ge \frac{ {{\,\textrm{tr}\,}}(A)\sqrt{\varepsilon }}{1+C \sqrt{\varepsilon }} \int _{\Omega } | \psi |^2\, dx \qquad \forall \psi \in H^1(\Omega ). \end{aligned}$$

Moreover, if $\Omega = \mathbb {R}^n$ we can take $C=0$ in the previous formula and equality holds if and only if $\psi$ is a multiple of $e^{-x^tAx/2\sqrt{\varepsilon }}$.

### Proof

First let us prove that we can take $C=0$ if $\Omega =\mathbb {R}^n$. In fact, for every $\psi \in C_c^{\infty }$ we can define $g(x)=\psi (x)e^{x^tAx/2}$. Then we have

$$\begin{aligned} |\nabla \psi |^2= &   \left| \nabla g e^{-x^tAx/2} - g Ax e^{-x^tAx/2} \right| ^2\\= &   \left( |\nabla g|^2 - \nabla ( g^2) \cdot Ax + g^2 |Ax|^2 \right) e^{-x^tAx}. \end{aligned}$$

Integrating this identity $\mathbb {R}^n$ and then using the integration by parts formula we get

$$\begin{aligned} \int _{\mathbb {R}^n} | \nabla \psi |^2 \, dx&= \int _{\mathbb {R}^n} | \nabla g|^2 e^{-x^tAx} \, dx+ \int _{\mathbb {R}^n} |Ax|^2 g^2 e^{-x^tAx}\, dx \\&\quad + \int _{\mathbb {R}^n} g^2 \nabla \cdot ( Ax e^{-x^tAx}) \, dx \\&\ge \int _{\mathbb {R}^n} |Ax|^2 g^2 e^{-x^tAx}\, dx + {{\,\textrm{tr}\,}}(A) \int _{\mathbb {R}^n} g^2 e^{-x^tAx}\, dx\\&\quad - 2 \int _{\mathbb {R}^n} |Ax|^2 g^2 e^{-x^tAx}\, dx\\&= - \int _{\mathbb {R}^n} | Ax|^2 | \psi |^2 \, dx + {{\,\textrm{tr}\,}}(A), \end{aligned}$$

where we used that $|\nabla g|^2$ is nonnegative and that $g^2e^{-x^tAx} = | \psi |^2$. Using this inequality with $A/\sqrt{\varepsilon }$ and using the density of $C_c^{\infty }$ in $H^1_0(\mathbb {R}^n)$ we have that

2.2 $$\begin{aligned} \int _{\mathbb {R}^n} \Bigl ( \varepsilon |\nabla \psi |^2 + | Ax|^2 | \psi |^2 \Bigr ) \, dx \ge {{\,\textrm{tr}\,}}(A)\sqrt{\varepsilon } \int _{\mathbb {R}^n} | \psi |^2\, dx \quad \forall \psi \in H^1(\mathbb {R}^n); \end{aligned}$$

moreover we can have equality in (2.2) using $g \equiv C_{\varepsilon }$. In particular the previous computation shows that $\psi _{\varepsilon } = C_{\varepsilon } e^{-x^tAx/2\sqrt{\varepsilon }}$ is the minimizer for $E^{\varepsilon }_{\mathbb {R}^n}$ (here $C_{\varepsilon }$ is the normalization constant such that $\int | \psi _{\varepsilon }|^2 =1$).^1^.

In the general case let us consider the cut off function $f_{r} (x)= \min \{1, \bigl ( \frac{|x|}{r} - 2\bigr )_+\}$. We have that $|\nabla f_r | \le \frac{1}{r} \chi _{B_{2r} \setminus B_r }$ and $f_r=1$ on $B_r$ while $f_r=0$ on $B_{2r}^c$. In particular, for every function $\psi \in H^1(\Omega )$, we have $f_r \psi \in H^1_0(\mathbb {R}^n)$. However, we can then apply (2.2) in order to obtain

2.3 $$\begin{aligned} \int _{\Omega } \Bigl ( \varepsilon | \nabla ( \psi f_r )|^2 + |Ax|^2 | \psi |^2f_r^2 \Bigr )\, dx \ge \sqrt{\varepsilon }{{\,\textrm{tr}\,}}(A) \int _{\Omega } | \psi |^2 f_r^2. \end{aligned}$$

Now, by Young’s inequality, we have that, for every $\delta <1$,

2.4 $$\begin{aligned} | \nabla ( \psi f_r )|^2= &   | \nabla \psi |^2 f_r^2 + | \nabla f_r|^2 | \psi |^2 + 2 \nabla f_r \cdot \nabla \psi f_r \psi \nonumber \\\le &   ( 1+ \delta ) | \nabla \psi |^2 f_r^2 + (1+\delta ^{-1}) | \nabla f_r|^2 | \psi |^2. \end{aligned}$$

Notice also that, since $|Ax|^2 \ge \lambda |x|^2 \ge \lambda ^2 r^2$ if $|x|\ge r$, we have

2.5 $$\begin{aligned} (1+\delta ^{-1})| \nabla f_r|^2 = \frac{\delta + 1}{\delta r^2 } \chi _{B_{2r} \setminus B_r} \le \frac{2}{\delta r^2 } \cdot \frac{|Ax|^2}{\lambda ^2 r^2}; \end{aligned}$$

moreover we also have that

2.6 $$\begin{aligned} {{\,\textrm{tr}\,}}(A) (1-f_r^2) \le n \Lambda \chi _{B_{2r} \setminus B_r}(1-f_r^2) \le \frac{n \Lambda |Ax|^2}{\lambda ^2 r^2}(1-f_r^2). \end{aligned}$$

Now, starting from Equation (2.3) and then adding the term $\sqrt{\varepsilon } {{\,\textrm{tr}\,}}(A) \int _{\Omega } | \psi |^2 (1-f_r^2)$ on both sides, we can use (2.6), (2.4) and (2.5) to obtain

$$\begin{aligned}  &   \int _{\Omega } \varepsilon (1+\delta ) |\nabla \psi |^2 f_r^2 + \left( f_r^2+ \frac{n\Lambda \sqrt{\varepsilon }}{\lambda ^2 r^2 }(1-f_r^2) + \frac{ 2 \varepsilon }{\delta r^4 \lambda ^2} \right) | Ax|^2 | \psi |^2 \, dx\\  &   \quad \ge \sqrt{\varepsilon } {{\,\textrm{tr}\,}}(A) \int _{\Omega } | \psi |^2\, dx; \end{aligned}$$

In particular, as long as $r^2\lambda ^2 \ge n \Lambda \sqrt{\varepsilon }$ we have $f_r^2+ \frac{n\Lambda \sqrt{\varepsilon }}{\lambda ^2 r^2 }(1-f_r^2) \le 1$ and then we optimize in $\delta$, choosing $\delta = \frac{\sqrt{2 \varepsilon }}{r^2 \lambda }$, getting

$$\begin{aligned} \left( 1+ \frac{\sqrt{2 \varepsilon } }{r^2 \lambda }\right) \cdot \int _{\Omega } \Bigl ( \varepsilon |\nabla \psi |^2 + | Ax|^2 | \psi |^2 \Bigr )\, dx \ge \sqrt{\varepsilon } {{\,\textrm{tr}\,}}(A) \int _{\Omega } | \psi |^2\, dx, \end{aligned}$$

which is precisely (2.1), with $C=\frac{ \sqrt{2}}{r^2 \lambda }$. $\square$

### Lemma 2.2

Let us consider a continuous potential $V \ge 0$ such that $V(0)=0$ and *V* is twice differentiable in 0. Then there exist a sequence $\psi _{\varepsilon }$ such that $|\psi _{\varepsilon }|^2 \rightharpoonup \delta _0$ and

2.7 $$\begin{aligned} \limsup _{\varepsilon \rightarrow 0} \int _{\mathbb {R}^d} \Bigl ( \varepsilon ^{1/2} \frac{ | \nabla \psi _{\varepsilon }|^2 }{2} + \varepsilon ^{-1/2} V(x) | \psi _{\varepsilon }|^2 \Bigr )\, dx \le \frac{1}{2} {{\,\textrm{tr}\,}}\left( \sqrt{ D^2V(0)} \right) . \end{aligned}$$

### Proof

First, for a positive definite symmetric matrix *A*, let us consider consider the function

$$\begin{aligned} f_{\varepsilon } = \left( e^{ - \frac{x^t A x }{ 2\sqrt{\varepsilon }} } - e^{-N} \right) _+. \end{aligned}$$

Then, letting $\Omega _{\varepsilon }= \{ f_{\varepsilon } \ne 0 \} =\{ x^t A x < 2N\sqrt{\varepsilon } \}$, we claim that

2.8 $$\begin{aligned} \frac{ \int _{\mathbb {R}^d} \varepsilon | \nabla f_{\varepsilon }|^2 + |Ax|^2 |f_{\varepsilon }|^2 \, dx }{ \int _{\mathbb {R}^d} |f_{\varepsilon }|^2 \, dx } = \sqrt{\varepsilon } {{\,\textrm{tr}\,}}(A) \cdot h(N)\end{aligned}$$

for some universal function *h*(*N*) such that $h(N) \rightarrow 1$ as $N \rightarrow \infty$. We postpone to the end the proof of the claim.

Now, since *V* has an absolute minimum at $x=0$ we have that $D^2V(0)$ is a positive semidefinite symmetric matrix .

Since *V* is twice differentiable at 0, for $\eta >0$ there exists an increasing continuous function $\delta (\eta )>0$ with $\delta (\eta )\rightarrow 0$ for $\eta \rightarrow 0$ such that

$$\begin{aligned} 2V(x) \le x^t D^2V(0) x + \eta |x|^2 \le |{(\Lambda + \sqrt{\eta }\textrm{Id})x}|^2 \quad \forall x \in B_{\delta _n}(0). \end{aligned}$$

Since the function $\frac{\sqrt{\eta }}{-\log \eta }$ is continuous and strictly increasing, for every $\varepsilon$ sufficiently small there exists a unique $\eta =\eta (\varepsilon )$ such that $\sqrt{\varepsilon }= \frac{\sqrt{\eta }\delta (\eta )^2}{-2\log \eta }$. Now, let $A=\sqrt{D^2V(0)} + \sqrt{\eta }\textrm{Id}$, $N=-\log \eta$ and $\psi _{\varepsilon }= f_{\varepsilon }/ \sqrt{ \int |f_{\varepsilon }|^2 }$. With this choice of parameters, we get $\Omega _{\varepsilon } \subseteq B_{\delta }(0)$ (using that $A \ge \sqrt{\eta }\textrm{Id}$). Since $\psi _{\varepsilon }$ is also normalized, we get that $|\psi _{\varepsilon }|^2 \rightharpoonup \delta _0$. Moreover $2V(x) \le |Ax|^2$ in $\Omega _{\varepsilon }$ and hence, we can say that

$$\begin{aligned}&\int _{\mathbb {R}^d} \Bigl ( \varepsilon ^{1/2} \frac{ | \nabla \psi _{\varepsilon }|^2}{2} + \varepsilon ^{-1/2} V(x) |\psi _{\varepsilon }|^2 \Bigr ) \, dx \\&\quad \le \frac{1}{2\sqrt{\varepsilon }} \int _{\mathbb {R}^d}\Bigl ( \varepsilon | \nabla \psi _{\varepsilon }|^2 + |Ax|^2|\psi _{\varepsilon }|^2\Bigr ) \, dx \\&\quad = \frac{1}{2} {{\,\textrm{tr}\,}}(A) \cdot h(-\log {\eta }) \\&\quad = \frac{1}{2} \left( {{\,\textrm{tr}\,}}\Bigl (\sqrt{D^2V(0)}\Bigr )+d \cdot \sqrt{\eta } \right) \cdot h(-\log {\eta }). \end{aligned}$$

Letting $\varepsilon \rightarrow 0$, hence $\eta (\varepsilon ) \rightarrow 0$, we deduce (2.7).

We now prove the claim (2.8). First of all,

$$\begin{aligned} |\nabla f_{\varepsilon }|^2= &   \frac{1}{\varepsilon } |Ax|^2 e^{ - \frac{ x^t A x }{ \sqrt{\varepsilon }} } \chi _{\Omega _{\varepsilon }}, \end{aligned}$$

2.9 $$\begin{aligned} |f_{\varepsilon }|^2= &   \chi _{\Omega _{\varepsilon }}(e^{ - \frac{x^t A x}{ \sqrt{\varepsilon }} } - 2 e^{ - \frac{ x^t A x }{2\sqrt{\varepsilon }} }e^{-N} + e^{-2N}). \end{aligned}$$

We make the change of variable $z=\frac{\sqrt{A}}{\root 4 \of {\varepsilon }} x$ which maps $\Omega _{\varepsilon })$ in $B_{\sqrt{2N}}(0)=B$. We obtain

$$\begin{aligned}&\frac{\det \sqrt{A}}{\varepsilon ^{1/2}} \int _{\Omega _{\varepsilon }} \varepsilon | \nabla f_{\varepsilon }|^2 + |Ax|^2 |f_{\varepsilon }|^2 \, dx \\&\quad =\int _{B} \sqrt{\varepsilon } z^t A z \cdot ( 2e^{-|z|^2} - 2e^{-\frac{|z|^2}{2} -N} + e^{-2N} ) \, dz \\&\quad = \sqrt{\varepsilon } \cdot \frac{{{\,\textrm{tr}\,}}(A)}{d} \int _B |z|^2 (2e^{-|z|^2} -2e^{-\frac{|z|^2}{2}-N} + e^{-2N})\, dz, \end{aligned}$$

where in the second step we used that in $z^tAz=\sum _{i,j}A_{ij}z_jz_i$ every term with $i\ne j$ integrates to 0 (when multiplied by the function in brackets) because the function is antisymmetric and every term $z_i^2$ gives the same integral as $|{z}|^2/d$ by symmetry. Performing a similar calculation for $\int | f_{\varepsilon }|^2$, we find that

$$\begin{aligned} \frac{ \int _{\Omega _{\varepsilon }} \varepsilon | \nabla f_{\varepsilon }|^2 + \frac{1}{\varepsilon } |Ax|^2 |f_{\varepsilon }|^2 \, dx }{ \int _{\Omega _{\varepsilon }} |f_{\varepsilon }|^2 \, dx } = \frac{{{\,\textrm{tr}\,}}(A)}{d} \frac{\int _B z^2 (2e^{-z^2} -2e^{-\frac{z^2}{2}-N} + e^{-2N})\, dz}{\int _B (e^{-z^2} -2e^{-\frac{z^2}{2}-N} + e^{-2N})\, dz}. \end{aligned}$$

Since $\int _{\mathbb {R}^d} 2z^2e^{-z^2}\, dz = d \int _{\mathbb {R}^d} e^{-z^2}$ it is easy to see that $h(N):= \frac{\int _B z^2 (2e^{-z^2} -2e^{-\frac{z^2}{2}-N} + e^{-2N})\, dz}{d\int _B (e^{-z^2} -2e^{-\frac{z^2}{2}-N} + e^{-2N})\, dz}$ is such that $h(N) \rightarrow 1$ as $N\rightarrow \infty$. $\square$

### Proof of Theorem 1.2

Starting from point (a) first of all we have that $| \psi _{\varepsilon }|^2 \rightharpoonup \gamma$ for some $\gamma \in \mathcal {M}_+(\mathbb {R}^n)$ with $\gamma ( \mathbb {R}^d) \le 1$. Then, assuming $\liminf _{\varepsilon \rightarrow 0} E_{\varepsilon }(\psi _{\varepsilon })=C < \infty$, we have that for every $\delta >0$, as $\{V > \delta \}$ is an open set

$$\begin{aligned} \gamma ( \{ V> \delta \})\le &   \liminf _{\varepsilon \rightarrow 0} \int _{ \{V > \delta \}} | \psi _{\varepsilon }|^2\\\le &   \liminf _{\varepsilon \rightarrow 0}\frac{\sqrt{\varepsilon }}{\delta }\cdot \frac{1}{\sqrt{\varepsilon }}\int _{ \mathbb {R}^d} V(x)| \psi _{\varepsilon }^2| \\\le &   \lim _{\varepsilon \rightarrow 0} \frac{\varepsilon }{\delta }C =0. \end{aligned}$$

By the arbitrariness of $\delta$ we conclude that $\gamma ( \{V>0\} ) =0$.

Now we define the energy density $\mu _{\varepsilon } = \left( \varepsilon ^{1/2} \frac{| \nabla \psi _{\varepsilon }|^2}{2} +\varepsilon ^{-1/2} V(x) |\psi _{\varepsilon }|^2\right) \mathcal {L}^d$; by hypotesis we know that the sequence $\mu _{\varepsilon }$ is uniformly bounded and in particular, up to subsequences, there exists a weak limit $\mu$. By the semicontinuity of the mass, it will be sufficient to prove that the Radon-Nikodym derivative of $\mu$ with respect to $\gamma$ is greater or equal than $\frac{1}{2} {{\,\textrm{tr}\,}}( \sqrt{D^2V} )$. In particular, using a result by [34] (see Theorem 5.3 and Theorem 5.7 in [4] for a clearer explanation) we have that

$$\begin{aligned} \frac{ d\mu }{d \gamma } (v_0)= \lim _{ r \rightarrow 0} \frac{ \mu (C_r)}{\gamma (C_r)}, \end{aligned}$$

where $C_r$ are cylinders centered in $v_0$, that is a set of the form $B^X_r(x_0) \times B^Y_r(y_0)$ for an orthogonal splitting $\mathbb {R}^d= X \times Y$ and $v_0=(x_0,y_0)$. It is crucial to notice that any such cylinder is convex and $B_r(v_0) \subseteq C_r \subseteq B_{\sqrt{2}r}(v_0)$: in particular $C_r \in \mathscr {S}_{\sqrt{2}}(v_0)$ in the notation of [4].

Thus we are done if for every $v_0 \in \{V=0\}$ and every $0<\delta <1$, for $r>0$ small enough there is an open cylinder $C_r$ such that

2.10 $$\begin{aligned} \mu (\overline{C_r})&\ge \liminf _{\varepsilon \rightarrow 0} \mu _{\varepsilon }( C_r) = \liminf _{\varepsilon \rightarrow 0} \left\{ \int _{C_r} \varepsilon ^{1/2} \frac{| \nabla \psi _{\varepsilon }|^2}{2} +\varepsilon ^{-1/2} V(x) |\psi _{\varepsilon }|^2 \, dx \right\} \\&\ge \frac{\sqrt{1-\delta }}{2} {{\,\textrm{tr}\,}}\left( \sqrt{D^2V(v_0)} \right) \gamma (C_r). \end{aligned}$$

In order to do this let us suppose that $D^2V \ne 0$, otherwise the statement is trivial. In that case, denote $A= \sqrt{ D^2 V (v_0)}$ and make an orthonormal change of coordinates such that $D^2V(v_0)$ becomes diagonal with the eigenvalues in decreasing order. Then, letting $n=\textrm{rk} (D^2V (v_0))$ we consider *X* the span of the first *n* eigenvectors and *Y* the remaining ones. In particular we have $c \textrm{Id}\le D^2V (v_0)|_X \le C \textrm{Id}$ for some $C>c>0$ (depending on $v_0$) and $D^2V|_Y=0$. Moreover, we have that $\nabla _XV(x_0,y_0)=0$ and $\nabla _X\nabla _XV(x_0,y_0)=D^2V(v_0)|_X$ is invertible, therefore by the implicit function theorem there exists $r>0$ and a $C^1$ function $m:B^y_r(y_0)\rightarrow B^x_r(x_0)$ such that $\nabla _XV(m(y),y)=0$ for every $y\in B^y_r(y_0)$. The gradient of the implicit function is

$$\begin{aligned} \nabla _Ym(y)=-(\nabla _{XX}V(m(y),y))^{-1} \nabla _{XY}V(m(y),y), \end{aligned}$$

which vanishes for $y=y_0$ since $\nabla _{XY}V(v_0)=0$. Therefore, up to possibly reducing *r*, we have that $|m(y)-x_0|\le \delta |y-y_0|$ for every $y\in B^y_r(y_0)$. By the continuity of $D^2V$, up to reducing *r* a second time, we have also $D^2V(x,y)|_X\ge (1-\delta )D^2V(x_0,y_0)|_X$ for every $(x,y)\in B^x_r(x_0)\times B^y_r(y_0)$. Therefore, for every $(x,y)\in B^x_r(x_0)\times B^y_r(y_0)$, we consider the second order Taylor expansion of *V* at (*y*, *m*(*y*)) (recall that $V \ge 0$ and that $\nabla _X V$ vanishes at this point) to find that

$$\begin{aligned} \begin{aligned} V(x,y)&\ge \frac{1-\delta }{2}(x-m(y))^T D^2V(v_0)|_X(x-m(y)) = \frac{1-\delta }{2} |A(x-m(y))|^2, \end{aligned} \end{aligned}$$

where we denoted that $Ax=A(x,0)$; since *A* is non degenerate only in the *x* directions in particular, we have $|Ax| \ge c|x|$ for some $c>0$ and ${{\,\textrm{tr}\,}}_x (A)={{\,\textrm{tr}\,}}(A)$.

Since $B^x_{r(1-\delta )}(m(y)) \subseteq B^x_r(x_0)$, we can use Lemma 2.1 in order to say that there exists a constant *C* such that, for every $y \in B_r^y(y_0)$,

$$\begin{aligned}&\int _{B^x_r(x_0)} \varepsilon \frac{| \nabla _x \psi _{\varepsilon } (x,y)|^2}{2} + V(x,y) | \psi _{\varepsilon }(x,y)|^2 \, dx \\&\quad \ge \frac{1}{2} \int _{B^x_r(x_0)} \varepsilon | \nabla _x \psi _{\varepsilon }|^2 + (1-\delta ) |A(x-m(y))|^2 | \psi _{\varepsilon }|^2 \, dx \\&\quad \ge \sqrt{\varepsilon } \frac{ \sqrt{1-\delta } }{1+C \sqrt{\varepsilon } } \cdot \frac{{{\,\textrm{tr}\,}}(A)}{2} \int _{B^x_r(x_0)} | \psi _{\varepsilon }(x,y)|^2 \, dx . \end{aligned}$$

Integrating with respect to *y* and then passing to the limit as $\varepsilon \rightarrow 0$ we obtain (2.9).

In order to prove part (b) we use Lemma 2.2 in case $\gamma = \delta _{v_0}$ and in the rest of the cases we argue by convexity: let us consider $\psi _{\varepsilon }^v(x):=\psi _\varepsilon (x-v)$ a recovery sequence for $\delta _v$ and let us define $f_{\varepsilon }^v = | \psi _{\varepsilon }^v|^2$. We notice that

$$\begin{aligned} \frac{1}{\sqrt{\varepsilon }}\int _{\mathbb {R}^d} \Bigl ( \varepsilon \frac{| \nabla \psi _{\varepsilon }^v|^2}{2} + V(x) | \psi _{\varepsilon }^v|^2\Bigr ) \, dx = \frac{1}{\sqrt{\varepsilon }}\int _{\mathbb {R}^d} \Bigl (\varepsilon \frac{ | \nabla f_{\varepsilon }^v|^2}{8f_{\varepsilon }^v} + V(x) f_{\varepsilon }^v \Bigr )\, dx \end{aligned}$$

denoting by $H_{\varepsilon } (f_{\varepsilon }^v)$ the functional in the right-hand side, we observe that $H_{\varepsilon }$ is convex. Let us consider moreover the set $V_{\varepsilon } \subseteq \{ V=0\}$ defined as

$$\begin{aligned} V_{\varepsilon } = \left\{ v \in \{ V=0 \} \; : \; H_{\varepsilon }(f_{\varepsilon }^v) \le \frac{1}{2} {{\,\textrm{tr}\,}}\Bigl (\sqrt{ D^2V}(v) \Bigr ) + 1 \right\} . \end{aligned}$$

By construction we have that $\limsup _{\varepsilon } H_{\varepsilon }(f_{\varepsilon }^v) \le \frac{1}{2} {{\,\textrm{tr}\,}}\Bigl (\sqrt{ D^2V}(v) \Bigr )$ for every *v*; in particular this implies that $\chi _{V_{\varepsilon }} \rightarrow \chi _{ \{V=0\} }$ pointwise.

Now given a measure $\gamma \in \mathcal {P}(\mathbb {R}^d)$ with $\gamma (\mathbb {R}^d)$ supported in $\{V=0\}$, by dominated convergence we get $\gamma (V_{\varepsilon }) \rightarrow 1$. In particular for $\varepsilon$ sufficiently small $\gamma (V_{\varepsilon }) \ge \frac{1}{2}$ and for such $\varepsilon$ we define the functions

$$\begin{aligned} f^{\gamma }_{\varepsilon } (x) = \frac{1}{ \gamma (V_{\varepsilon })} \int _{ V_{\varepsilon }} f^{v}_{\varepsilon } (x) \, d \gamma . \end{aligned}$$

Consider now $\psi _{\varepsilon } (x)= \sqrt{ f^{\gamma }_{\varepsilon } (x) }$. By construction we have $\int | \psi _{\varepsilon }|^2 =1$; moreover, by testing with a continuous function we see that $|\psi _{\varepsilon }|^2 \rightharpoonup \gamma$ and thanks to the convexity of $H_{\varepsilon }$ we get that

$$\begin{aligned} \limsup _{\varepsilon \rightarrow 0} E_{\varepsilon } (\psi _{\varepsilon })&= \limsup _{\varepsilon \rightarrow 0} H_{\varepsilon } (f^{\gamma }_{\varepsilon }) \le \limsup _{\varepsilon \rightarrow 0} \frac{1}{\gamma (V_{\varepsilon })} \int _{V_{\varepsilon }} H_{\varepsilon }( f^v_{\varepsilon }) \, d \gamma \\&= \limsup _{\varepsilon \rightarrow 0} \int _{\mathbb {R}^d} \frac{\chi _{V_{\varepsilon }}}{\gamma (V_{\varepsilon })} E_{\varepsilon }( \psi ^v_{\varepsilon }) \, d \gamma .\end{aligned}$$

Now let us consider the functions $g_{\varepsilon } (v) = \frac{\chi _{V_{\varepsilon }}(v)}{\gamma (V_{\varepsilon })} E_{\varepsilon }( \psi ^v_{\varepsilon })$ and $g(v) = {{\,\textrm{tr}\,}}\bigl (\sqrt{ D^2V(v) } \bigr )$; if *g* is not $\gamma$-integrable there is nothing to prove; so we can assume $g \in L^1(\gamma )$. By the definition of $V_{\varepsilon }$, for $\varepsilon$ sufficiently small we have that

$$\begin{aligned} \frac{\chi _{V_{\varepsilon }}(v)}{\gamma (V_{\varepsilon })} E_{\varepsilon }( \psi ^v_{\varepsilon }) \le {{\,\textrm{tr}\,}}\Bigl (\sqrt{ D^2V(v) } \Bigr ) + 1, \end{aligned}$$

and we notice that the right-hand side is $\gamma$-integrable, otherwise there is nothing to prove. By Fatou’s lemma, using also that $\gamma (V_{\varepsilon }) \rightarrow 1$, $\chi _{V_{\varepsilon }} \rightarrow 1$, we have

$$\begin{aligned} \limsup _{\varepsilon \rightarrow 0} E_{\varepsilon }(\psi _{\varepsilon })\le &   \limsup _{\varepsilon \rightarrow 0} \int _{\mathbb {R}^d} \frac{\chi _{V_{\varepsilon }}}{\gamma (V_{\varepsilon })} E_{\varepsilon }( \psi ^v_{\varepsilon }) \, d \gamma \\\le &   \int _{\mathbb {R}^d} \limsup _{\varepsilon \rightarrow 0} \frac{\chi _{V_{\varepsilon }}}{\gamma (V_{\varepsilon })} E_{\varepsilon }( \psi ^v_{\varepsilon }) \, d \gamma \\\le &   \frac{1}{2} \int _{\mathbb {R}^d} {{\,\textrm{tr}\,}}( \sqrt{ D^2V} ) \, d \gamma . \end{aligned}$$

$$\square $$

## Rectangular truncation of Gaussians

As described in the introduction, Gaussian densities are asymptotically optimal for the energy of a single delta, therefore, to approach the general case, it is natural to construct a recovery sequence which is a superposition of Gaussian kernels. However, since we do not have global estimates on the potential *V*, only short-range interactions can be allowed, hence we need the kernels to have compact support. To achieve this, we truncate the densities with a slowly growing parameter *N*. The construction we present in Sect. 4.3 of the recovery sequence for Theorem 1.3 is then given by a convolution of *x*-dependent suitably rescaled and truncated Gaussian-like kernels, whose major axis is parallel at each point to ${{\,\textrm{graph}\,}}T$.

The requirements discussed above leads us to introduce some kernels which resemble Gaussian densities but have compact support, built in a suitable way that guarantees good properties for both the kinetic and potential energy.

Let *M* be a positive definite symmetric $2\times 2$ matrix with eigenvalues $a\ge b>0$ and let *w* and *z* be the orthonormal coordinates in the direction of the corresponding eigenvectors. For $N\in \mathbb {R}$ we define the unnormalized Gaussian and unnormalized truncated Gaussian

$$\begin{aligned} {\tilde{\Gamma }}_{M,\infty }({{\varvec{x}}})&= e^{-{{\varvec{x}}}^TM{{\varvec{x}}}} = e^{-aw^2} e^{-bz^2}, \\ {\tilde{\Gamma }}_{M,N}({{\varvec{x}}})&= \left( e^{-aw^2/2} - e^{-N/2}\right) _+^2 \left( e^{-bz^2/2} - e^{-N/2}\right) _+^2 . \end{aligned}$$

In the definition of these truncated kernels, we adopt a rectangular truncation, which leads to kernels of product form; we call it rectangular because the support of the truncated Gaussian is a tilted rectangle, aligned with the eigenvectors of the matrix. This structure is crucial to do the computations in Sect.  6.1. Moreover, instead of the obvious truncation, we have to truncate the square root of the Gaussian, and then put a square outside, to guarantee the finiteness of the kinetic energy.

In the proof of the main theorem we will apply the results of this section to the matrix

$$\begin{aligned} M = M_{\varepsilon ,\beta }(x) = \frac{A(x)}{\varepsilon ^{1/2}} + \frac{I}{\beta }, \end{aligned}$$

where $A(x)=\sqrt{\nabla ^2V\bigl (x,T(x)\bigr )}$, hence $a=q(x)/\varepsilon ^{1/2}+1/\beta$ and $b=1/\beta$, with *q*(*x*) being the non-zero eigenvalue of *A*(*x*). The parameter $\beta \gg \varepsilon ^{1/2}$ is required to ensure that the resulting Gaussian has compact support, since $\nabla ^2V\bigl (x,T(x)\bigr )$ is a singular matrix, hence the natural choice of $M=A(x)/\varepsilon ^{1/2}$ wouldn’t work properly. We will discuss more about $\beta$ when we present all the parameters in Sect.  4.2.

Define now the one dimensional integrals

$$\begin{aligned} G_{\alpha ,\infty }&= \int _\mathbb {R}e^{-\alpha t^2} \textrm{d}t = \frac{\sqrt{\pi }}{\sqrt{\alpha }}, \\ G_{\alpha ,N}&= \int _\mathbb {R}\left( e^{-\alpha t^2/2}-e^{-N/2}\right) _+^2 \textrm{d}t = \int _{-\sqrt{N/\alpha }}^{\sqrt{N/\alpha }} \left( e^{-\alpha t^2/2}-e^{-N/2}\right) ^2 \textrm{d}t, \end{aligned}$$

and the integrals of the two dimensional densities

$$\begin{aligned} G_{M,\infty }&= \int _{\mathbb {R}^2} {\tilde{\Gamma }}_{M,\infty }({{\varvec{x}}}) \textrm{d}{{\varvec{x}}}= G_{a,\infty } G_{b,\infty } = \frac{\pi }{\sqrt{\det M}}, \\ G_{M,N}&= \int _{\mathbb {R}^2} {\tilde{\Gamma }}_{M,N}({{\varvec{x}}}) \textrm{d}{{\varvec{x}}}= G_{a,N} G_{b,N} . \end{aligned}$$

We can now introduce the normalized Gaussian and truncated Gaussian, which are probability densities, given by

3.1 $$\begin{aligned} \Gamma _{M,N}({{\varvec{x}}}) = \frac{{\tilde{\Gamma }}_{M,N}({{\varvec{x}}})}{G_{M,N}}, \qquad N\in \mathbb {R}\cup \{\infty \}. \end{aligned}$$

Finally, let $\eta _{M,\infty }$ and $\eta _{M,N}$ be the first marginal of $\Gamma _{M,\infty }$ and $\Gamma _{M,N}$, respectively.

We present here two lemmas, but we postpone their proof to Sect.  7.

### Lemma 3.1

With the definition above, there exists a constant $C>0$ such that, for every $N \ge 3$, we have

3.2 $$\begin{aligned} G_{M,N}< G_{M,\infty }&< G_{M,N} + Ce^{-N/2}G_{M,\infty } , \end{aligned}$$

3.3 $$\begin{aligned} \Vert {\Gamma _{M,N}-\Gamma _{M,\infty }}\Vert _\infty&\le C\sqrt{\det M} e^{-N/2} , \end{aligned}$$

3.4 $$\begin{aligned} \Vert {\Gamma _{M,N}-\Gamma _{M,\infty }}\Vert _1&\le C N e^{-N/2} , \end{aligned}$$

3.5 $$\begin{aligned} \Vert {\eta _{M,N}-\eta _{M,\infty }}\Vert _\infty&\le C\sqrt{a} \sqrt{N} e^{-N/2} . \end{aligned}$$

Let us now compute the kinetic and potential energies of a Gaussian. In analogy with (1.9) we define the kinetic energy of a plan $\Gamma \in \mathscr {P}(\mathbb {R}^2)$ as

3.6 $$\begin{aligned} {{\,\textrm{KE}\,}}(\Gamma ) = \frac{1}{2} \int _{\mathbb {R}^2} \left|{\nabla \sqrt{\Gamma ({{\varvec{x}}})}}\right|^2 \textrm{d}{{\varvec{x}}}= \int _{\mathbb {R}^2} \frac{|{\nabla \Gamma ({{\varvec{x}}})}|^2}{8\Gamma ({{\varvec{x}}})}\textrm{d}{{\varvec{x}}}. \end{aligned}$$

A direct computation shows that, if $Be_w=fe_w$ and $Be_z=ge_z$, then

$$\begin{aligned} \int _{\mathbb {R}^2} |{B{{\varvec{x}}}}|^2 \Gamma _{M,\infty }({{\varvec{x}}}) \textrm{d}{{\varvec{x}}}= &   \int _{\mathbb {R}^2} (f^2w^2+g^2z^2) \frac{\sqrt{ab}}{\pi } e^{-aw^2-bz^2} \textrm{d}w \textrm{d}w\\= &   \frac{1}{2} \left( \frac{f^2}{a} + \frac{g^2}{b}\right) . \end{aligned}$$

Therefore, using $\left|{\nabla \sqrt{\Gamma _{M,\infty }}}\right|^2 = \frac{1}{2}\left|{\nabla [\log \Gamma _{M,\infty }]}\right|^2 \Gamma _{M,\infty } = |{M{{\varvec{x}}}}|^2 \Gamma _{M,\infty }({{\varvec{x}}})$ and the previous identity with $B=M$ we get

3.7 $$\begin{aligned} \begin{aligned} {{\,\textrm{KE}\,}}(\Gamma _{M,\infty })&= \frac{1}{2} \int _{\mathbb {R}^2} |{M{{\varvec{x}}}}|^2 \Gamma _{M,\infty }({{\varvec{x}}}) \textrm{d}x = \frac{{{\,\textrm{tr}\,}}M}{4} . \end{aligned} \end{aligned}$$

The following lemma compares the potential energy associated to the quadratic potential induced by the matrix *M* and the kinetic energy of the Gaussian $\Gamma _{M,\infty }$ and the truncated Gaussian $\Gamma _{M,N}$.

### Lemma 3.2

There is a universal constant $C>0$ such that

3.8 $$\begin{aligned}  &   \int _{\mathbb {R}^2} |{M{{\varvec{x}}}}|^2 |{\Gamma _{M,N}({{\varvec{x}}})-\Gamma _{M,\infty }({{\varvec{x}}})}| \textrm{d}{{\varvec{x}}}\le C{{\,\textrm{tr}\,}}(M) N e^{-N/2} , \end{aligned}$$

3.9 $$\begin{aligned}  &   |{{{\,\textrm{KE}\,}}(\Gamma _{M,N}) - {{\,\textrm{KE}\,}}(\Gamma _{M,\infty })}| \le C {{\,\textrm{KE}\,}}(\Gamma _{M,\infty }) e^{-N/2} = C {{\,\textrm{tr}\,}}(M) e^{-N/2} .\nonumber \\ \end{aligned}$$

## Construction of the recovery sequence

### Structure of optimal plans, maps and potentials in one dimension

In this section we comment on the assumption on the boundedness of the second derivative of the Kantorovich potential *u* in Theorem 1.3 and we provide a class of $\rho$ for which this assumption is satisfied and the main Theorem 1.3 is applicable: we will use the structural results for the 1D Coulomb multimarginal optimal transport problem contained in [9], which allows us to transfer the regularity of $\rho$ to information on *V* and the optimal maps.

Let $\rho \in C^1_\textrm{loc}(\mathbb {R})$ be a strictly positive probability density. Without loss of generality, by translating we may assume that the origin 0 is the median of the probability $\rho$, i.e. $\rho \bigl ((-\infty ,0]\bigr )=\rho \bigl ([0,\infty )\bigr )=1/2$. By the result in [9], the unique optimal plan is induced by a map *T* from $\rho$ to itself which can be written explicitly in terms of the repartition function of $\rho$, which is $C^2$ with strictly positive first derivative. As a consequence, we have that $T\in C^2(\mathbb {R}\setminus \{0\})$, *T* is increasing in $\mathbb {R}_-$ and $\mathbb {R}_+$, $T(\mathbb {R}_-)=\mathbb {R}_+$, $T(\mathbb {R}_+)=\mathbb {R}_-$ and

$$\begin{aligned} \lim _{x\rightarrow -\infty } T(x)&= 0,&\lim _{x\rightarrow 0^-} T(x)&= \infty ,&\lim _{x\rightarrow 0^+} T(x)&= -\infty ,&\lim _{x\rightarrow +\infty } T(x)&= 0. \end{aligned}$$

The Kantorovich potential $u:\mathbb {R}\rightarrow \mathbb {R}$ satisfies by definition $u(x) + u(y) \le |x-y|^{-1}$ with equality if and only if $y= T(x)$; hence, using the first order condition of the maximality of $u(x)+u(y)-1/|x-y|$ at points $(x,y)=(x, T(x))$, we get

4.1 $$\begin{aligned} u'(x) = - \frac{{{\,\textrm{sign}\,}}\bigl (x-T(x)\bigr )}{\bigl (T(x)-x\bigr )^2} = - \frac{{{\,\textrm{sign}\,}}(x)}{\bigl (T(x)-x\bigr )^2} \qquad \forall x \in \mathbb {R}\setminus \{0\}, \end{aligned}$$

therefore *u* is determined up to a constant. Let *V* be as in (1.3), which in $d=1$ and $N=2$ reads as

4.2 $$\begin{aligned} V(x,y) = |x-y|^{-1} - u(x) - u(y) \qquad \text{ for } x,y\in \mathbb {R}. \end{aligned}$$

Assuming that $x<0$ and $y>0$ (the other case is analogous), we can compute the gradient of the potential

$$\begin{aligned} \nabla V(x,y) = \begin{pmatrix} \frac{1}{(y-x)^2} - u'(x) \\ -\frac{1}{(y-x)^2} - u'(y) \end{pmatrix} ; \end{aligned}$$

notice that $\nabla V\bigl (x,T(x)\bigr )=0$. Differentiating again, we get the Hessian

4.3 $$\begin{aligned} \nabla ^2V(x,y) = \frac{2}{(y-x)^3}\begin{pmatrix}1 &  -1 \\ -1 &  1\end{pmatrix} - \begin{pmatrix} u''(x) &  0 \\ 0 &  u''(y) \end{pmatrix}. \end{aligned}$$

In the proof of Theorem 1.3 we are interested in its behavior in a neighborhood of the graph of *T*, i.e. when $y=T(x)$.

The aim of the next lemma is twofold: first, we provide an assumption on the tails of $\rho$ which is sufficient to obtain boundedness of the second derivatives of *u*. Second, we show how the assumption on the boundedness of $u''$ will be used in the rest of the paper: through the computation of the Hessian of *V*, it will provide a sufficient condition to control the growth of *V* with a (uniform) parabola around the graph of *T*. We expect Theorem 1.3 to hold even without the uniform growth condition (4.4) below, but at the price of several technical complications that we don’t address here.

#### Lemma 4.1

Let $\rho \in C^1_\textrm{loc}(\mathbb {R})$ be a strictly positive probability density, let *u* be its Kantorovich potential and let *V* as in (4.2).

(i) If $u'' \in L^\infty (\mathbb {R})$, then there exists $\varepsilon _0,C>0$ such that
  4.4 $$\begin{aligned} V(x,y) \le C \textsf{d}\bigl ((x,y),{{\,\textrm{graph}\,}}(T)\bigr )^2 \qquad \forall (x,y) \text { s.t.}\, \textsf{d}\bigl ((x,y),{{\,\textrm{graph}\,}}(T)\bigr )< \varepsilon _0; \end{aligned}$$
(ii) if $\rho$ satisfies also (1.10), then $u'' \in L^\infty (\mathbb {R})$ and $\nabla ^2V$ is locally Lipschitz in a neighborhood of ${{\,\textrm{graph}\,}}(T)$.

#### Proof

Since $\rho \in L^1(\mathbb {R})$, there exists $\delta >0$ such that every interval *I* of length $<\delta$ has $\int _I \rho (x) \, dx <1/2$. Since $\int _x^{T(x)} \rho (x')\, dx'=1/2$ for every $x <0$ and $\int _{T(x)}^x \rho (x')\, dx' =1/2$ for every $x >0$, we obtain that $|T(x) - x|>\delta$, namely that ${{\,\textrm{graph}\,}}(T)$ has positive distance from the diagonal. The hessian of *V* is given by (4.3) and,

in a neighborhood of ${{\,\textrm{graph}\,}}(T)$, the first term is bounded because we are far from the diagonal $y=x$, whereas the second term is bounded everywhere since we have proved that $u''\in L^\infty (\mathbb {R})$. This implies that *V* has controlled quadratic growth in a neighborhood of ${{\,\textrm{graph}\,}}(T)$, namely that $V(x,y) \le C \textsf{d}\bigl ((x,y),{{\,\textrm{graph}\,}}(T)\bigr )^2$ in $\mathbb {R}^2 \setminus \{|x-y|< \varepsilon$ for any $\varepsilon >0$. The size $\varepsilon$ of the neighborhood only depends on $\rho$ (and in particular on the positive distance between the graph of the optimal map *T* and the diagonal $\{x=y\}$). This establishes (i).

Let us now prove (ii). From the formula (4.1) and the fact that $T\in C^1_\textrm{loc}(\mathbb {R}\setminus \{0\})$ we deduce that $u\in C^2_\textrm{loc}(\mathbb {R}\setminus \{0\})$. We need to check that $u''(x)$ stays bounded as $x\rightarrow 0^\pm$ and $x\rightarrow \pm \infty$. For $x<0$, we have that

$$\begin{aligned} u''(x) = \frac{\textrm{d}}{\textrm{d}x}\left[ \frac{1}{\bigl (T(x)-x\bigr )^2}\right] = 2\frac{1}{\bigl (T(x)-x\bigr )^3} - 2 \frac{T'(x)}{\bigl (T(x)-x\bigr )^3}. \end{aligned}$$

In both cases when $x\rightarrow -\infty$ or $x\rightarrow 0^-$, the first fraction goes to 0, therefore $u''(x)$ has the same asymptotic behavior as $T'(x)/\bigl (T(x)-x\bigr )^3$, let it be having a limit, being bounded or diverging. By the change of variable formula, we have moreover that

$$\begin{aligned} \frac{T'(x)}{\bigl (T(x)-x\bigr )^3} = \frac{\rho (x)}{\bigl (T(x)-x\bigr )^3\rho \bigl (T(x)\bigr )}, \end{aligned}$$

so our goal is to show that this fraction stays bounded as $x\rightarrow -\infty$ or $x\rightarrow 0^-$. The quantity is clearly non-negative, so we need to prove that the $\limsup$ is finite. It is immediately appearent that

$$\begin{aligned} \limsup _{x\rightarrow 0^-} \frac{T'(x)}{\bigl (T(x)-x\bigr )^3}= &   \limsup _{x\rightarrow 0^-} \frac{\rho (x)}{T(x)^3\rho \bigl (T(x)\bigr )} = \limsup _{y\rightarrow \infty } \frac{\rho (0)}{y^3\rho (y)}\\= &   \frac{\rho (0)}{\liminf \limits _{y\rightarrow \infty } y^3\rho (y)} < \infty , \end{aligned}$$

because of (1.10). Let us now turn to studying the limit for $x\rightarrow -\infty$. Since $T(x)\rightarrow 0^+$ for $x\rightarrow -\infty$, we have

$$\begin{aligned} \limsup _{x\rightarrow -\infty } \frac{T'(x)}{\bigl (T(x)-x\bigr )^3} = \limsup _{x\rightarrow -\infty } \frac{\rho (x)}{-x^3\rho \bigl (T(x)\bigr )} = \limsup _{x\rightarrow -\infty } \frac{\rho (x)}{-x^3\rho (0)} < \infty , \end{aligned}$$

because $$\rho \in L^\infty (\mathbb {R})$$.

Exactly the same can be said for $x\rightarrow 0^+$ and $x\rightarrow \infty$, and therefore we have that $u\in C^2(\mathbb {R})$ and $u''\in L^\infty (\mathbb {R})$.

Notice moreover that by (4.1) and the fact that $T\in C^2(\mathbb {R}\setminus \{0\})$ it follows that $u\in C^3(\mathbb {R}\setminus \{0\})$. Hence, denoting $D\subset \mathbb {R}^2$ the diagonal, by (4.3) we have that $\nabla ^2V$ is locally Lipschitz in $(\mathbb {R}\setminus \{0\})^2\setminus D$, which is a neighborhood of ${{\,\textrm{graph}\,}}(T)$. $\square$

Observe that (1.10) and ${{\,\textrm{KE}\,}}(\rho )<\infty$ are simultaneously satisfied if

$$\begin{aligned} \liminf _{|{x}|\rightarrow \infty }{} |{x}|^{\alpha +1} \rho '(x) > 0 \qquad \text {and} \qquad \limsup _{|{x}|\rightarrow \infty }{} |{x}|^{\beta +1} \rho '(x) < \infty \end{aligned}$$

for $$\alpha , \beta \in (1,3)$$ with $$1< \beta \le \alpha < 2\beta +1$$.

### Choice of the parameters

The construction of ${\bar{\gamma }}_\varepsilon$ depends on the choice of many parameters. Here we introduce them and specify which inequalities between them are necessary for the construction. First of all, we fix a parameter $H>1$. All the constructions in the following sections depend on *H*, which is considered as a fixed parameter, but to ease the notation we don’t always make this dependence explicit. This parameter *H* will play a role in the proof of Theorem 1.3 where we then use a diagonal argument to extract a recovery sequence.

Our construction relies on several structural assumptions. First of all, we need to split the mass in two components: the main part, where most of the energy comes from, and a remaining mass, used to fix the marginals of the recovery sequence. In order to do so, we need to lower the mass $\rho$ by some constant, and for this reason we have to restrict ourselves to the bulk of the mass and avoid the tails at $\pm \infty$. Moreover, in order to be able to estimate the potential energy, we require a linearization of the map *T* (for the application of Proposition 6.3), whose error depends on $T''$. Again for the application of Proposition 6.3, we want the slope of the linearized map to be far from 0 and $\infty$. Since *T* has asymptotes at $\pm \infty$ and 0, we want then to work in a domain that stays away from those regions. Finally, we need a control on the eigenvalue of $\nabla ^2V$ evaluated on ${{\,\textrm{graph}\,}}T$, as this will determine the width of the Gaussians used in the construction.

All these reasons motivate the introduction of a set where we have good estimates of the objects intervening in the construction. The invariant domain that we will use is the following. Given $H>0$ such that $\rho \bigl ([H,\infty )\bigr )<1/4$, define $0<r_H<H$ such that $\rho \bigl ([0,r_H]\bigr )=\rho \bigl ([H,\infty )\bigr )$ and consider the set

4.5 $$\begin{aligned} \Omega _H = [T(r_H),T(H)] \cup [r_H,H] \end{aligned}$$

and the enlarged set

4.6 $$\begin{aligned} \Omega _H' = [T(r_{H+1}),T(H+1)] \cup [r_{H+1},H+1] \supset \Omega _H. \end{aligned}$$

We have that $T(\Omega _H)=T^{-1}(\Omega _H)=\Omega _H$ and $\Omega _H\nearrow \mathbb {R}\setminus \{0\}$, hence $\int _{\Omega _H^c}\rho (x)\textrm{d}x\rightarrow 0$. Moreover $\rho \bigl (\Omega _H\cap (-\infty ,0]\bigr )=\rho \bigl (\Omega _H\cap ([0,\infty )\bigr )$ and similarly for $\Omega _H'$.

Let $q(x) = \lambda _1 \bigl (\nabla ^2V(x,T(x))\bigr )$ the positive eigenvalue. Notice that $q(x)>0$ because, as already mentioned, from (4.3) follows that $\nabla ^2 V$ cannot be the zero matrix. Moreover, *q* is a continuous function because the roots of a polynomial are continuous with respect to the coefficients.

As mentioned above, we want a control on some crucial quantities that influence the estimates of our construction. We therefore introduce the constant

4.7 $$\begin{aligned} \begin{aligned} L = L(H)&= \max \Bigl \{ \Vert {\rho }\Vert _{L^\infty (\Omega _H')},\ \Vert {1/\rho }\Vert _{L^\infty (\Omega _H')},\ \Vert {1/T'}\Vert _{L^\infty (\Omega _H')},\ \Vert {T}\Vert _{C^2(\Omega _H')},\\&\quad \Vert {u}\Vert _{C^2(\Omega _H')},\ \Vert {q}\Vert _{L^\infty (\Omega _H')},\ \Vert {1/q}\Vert _{L^\infty (\Omega _H')}, \\&\quad {{\,\textrm{Lip}\,}}\left( \rho |_{\Omega _H'}\right) ,\ {{\,\textrm{Lip}\,}}\left( u''|_{\Omega _H'}\right) \Bigr \}. \end{aligned} \end{aligned}$$

We now introduce the parameters on which our construction depends. They all depend on $\varepsilon >0$, the variable that indexes the sequence of energies and the recovery sequence. First of all, we fix a parameter $N = N(\varepsilon ) \rightarrow \infty$ faster than $\log \varepsilon$, which will be used to truncate the Gaussian-like kernels so that they have compact support such that

4.8 $$\begin{aligned} N(\varepsilon ) = |{\log \varepsilon }|^{5/4} ; \end{aligned}$$

then a parameter $\beta = \beta (\varepsilon ) \rightarrow 0$ such that

4.9 $$\begin{aligned} \varepsilon ^{1/2}N \ll \beta \ll \varepsilon ^{2/5} , \end{aligned}$$

used to desingularize the matrix $\sqrt{\nabla ^2V(x,T(x))}$; then a parameter $\delta = \delta (\varepsilon ) \rightarrow 0$ such that

4.10 $$\begin{aligned} (\beta N )^{1/2}\ll \delta \ll \varepsilon ^{1/8} N^{-3/5} , \end{aligned}$$

which controls the resolution of the linearization of the map *T*; and finally a parameter $\tau = \tau (\varepsilon ) \rightarrow 0$ such that

4.11 $$\begin{aligned} \tau&\gg \frac{\delta ^2}{\varepsilon ^{1/4}} ,&\tau&\gg \frac{\varepsilon ^{1/2}N^2}{\beta } ,&\tau&\gg \frac{\beta \delta N}{\varepsilon ^{1/2}} \gg \frac{\beta \delta ^2}{\varepsilon ^{1/2}} ,&\tau&\gg \beta ^{1/2}N^{1/2} \gg \varepsilon ^{1/4} \gg \beta , \end{aligned}$$

which controls the amount of mass that we subtract from $\rho$ in order to subdivide it in main mass and remaining mass.

In the following sections, we will write many of the estimates relying only on these inequalities, so that their use will be easier in the future. Finally, we provide a choice of the parameters that fulfills the previous inequalities:

4.12 $$\begin{aligned}&N(\varepsilon ) = |{\log \varepsilon }|^{5/4}, \quad \beta (\varepsilon ) = \varepsilon ^{1/2} |{\log (\varepsilon )}|^3, \quad \delta (\varepsilon ) = \varepsilon ^{1/8} |{\log \varepsilon }|^{-1}, \nonumber \\&\tau (\varepsilon ) = |{\log \varepsilon }|^{-1/3}. \end{aligned}$$

### Construction of ${\bar{\gamma }}_\varepsilon$

Let *T* be the optimal map between $\rho$ and itself with respect to the Coulomb cost, which induces the plan $\gamma$. For every $\bigl (x,T(x)\bigr ) \in \mathbb {R}^2$ on the graph of *T*, let us consider the truncated Gaussian $\Gamma _{M_{\varepsilon ,\beta (x)},N}$ given by (3.1) with covariance matrix $M_{\varepsilon ,\beta }(x)=\sqrt{D^2V(x,T(x))}/\varepsilon ^{1/2}+ \textrm{Id}/\beta$ and truncated through *N*.

We subdivide $\Omega _H$ into intervals $I^1_i=[a_i,b_i]$ of length $\delta /2<b_i-a_i<\delta$. Let $T_\delta$ be the piecewise linear interpolation of *T* on the intervals $I^1_i=[a_i,b_i]$, namely a map that is affine in each $I^1_i$ and which coincides with *T* at the boundary of each interval. We define also $T_\delta =T$ outside of $\Omega _H$. With this definition, writing $T(a_i)$ and $T(b_i)$ in terms of the Taylor expansion of *T* at *x*, we observe that

4.13 $$\begin{aligned} |{T_\delta (x)- T(x)}|\le &   \left|{ \frac{x-a_i}{b_i-a_i} T(a_i) + \frac{b_i-x}{b_i-a_i} T(b_i) - T(x) }\right|\nonumber \\\le &   \Vert {T''}\Vert _{L^\infty (\Omega _H)} \delta ^2 \le L\delta ^2 \end{aligned}$$

for every $x\in I^1_i$ and for every *i*. Let $I^2_i=T(I^1_i)=T_\delta (I^1_i)$. Fix also points $x_i\in I_i^1$ such that $T'(x_i)=T_\delta '(x_i)$ that will be used to freeze the coefficients of the truncated Gaussians. The existence of such a point is ensured by Lagrange Theorem.

For convenience, in the following we use the notation $\Gamma _{M,N}^{{\varvec{x}}}({{\varvec{x}}}')=\Gamma _{M,N}({{\varvec{x}}}'-{{\varvec{x}}})$ to denote the Gaussian with covariance matrix *M*, truncated at level *N*, centered at ${{\varvec{x}}}$.

Ideally, we would like to build a recovery sequence which is a superposition of Gaussians on ${{\,\textrm{supp}\,}}\gamma ={{\,\textrm{graph}\,}}T$ as

$$\begin{aligned} \gamma _\varepsilon ({{\varvec{x}}}') = \int _{\mathbb {R}^2} \Gamma _{M_{\varepsilon ,\beta }(x),N}^{{{\varvec{x}}}}({{\varvec{x}}}') \textrm{d}\gamma ({{\varvec{x}}}) = \int _\mathbb {R}\Gamma _{M_{\varepsilon ,\beta }(x),N}^{(x,T(x))}({{\varvec{x}}}') \textrm{d}\rho (x) . \end{aligned}$$

Unfortunately, this plan has the wrong marginals, and there isn’t a simple way to fix them without changing too much the energy. For instance one can notice that the construction in [1] pays an excessive price in the deconvolution step (this is analogous to applying Proposition 5.1 to the whole transport plan). We follow therefore a different route.

The actual construction of the recovery sequence is more involved. We split the total mass $\rho$ in two components: a main portion of the mass and a remaining mass. The main part is dealt with in this section by superposing some truncated Gaussian kernels, whereas the remaining mass is used to bring back the marginals to what they need to be (see Sect.  5) without disrupting the energy estimates (see Sect.  6).

The main part of the recovery sequence is a suitable approximation of $\gamma _\varepsilon$, which is given by the plan

4.14 $$\begin{aligned} {\bar{\gamma }}_\varepsilon ({{\varvec{x}}}') = \sum _{i=1} {\bar{\gamma }}_{\varepsilon ,i}({{\varvec{x}}}'), \end{aligned}$$

where each piece is built by superposing along the graph of $T_\delta$ Gaussians with frozen covariance matrix $M_{\varepsilon ,\beta }(x_i)$ with $x_i\in I^1_i$, wheighing them with the density $\rho -\tau$ (which is positive in $\Omega _H'$ for $\tau$, and hence $\varepsilon$, sufficiently small):

4.15 $$\begin{aligned} \begin{aligned} {\bar{\gamma }}_{\varepsilon ,i}({{\varvec{x}}}')&= \int _{I^1_i} \Gamma _{M_{\varepsilon ,\beta }(x_i),N}^{(x,T_\delta (x))}({{\varvec{x}}}') (\rho (x)-\tau ) \textrm{d}x \\&= \int _{I^1_i} \Gamma _{M_{\varepsilon ,\beta }(x_i),N}\bigl ({{\varvec{x}}}'-(x,T_\delta (x))\bigr ) (\rho (x)-\tau ) \textrm{d}x \\&= (\textrm{Id},T_\delta )_\#\bigl ((\rho -\tau ){\varvec{1}}_{I^1_i}\bigr ) * \Gamma _{M_{\varepsilon ,\beta }(x_i),N} ({{\varvec{x}}}'). \end{aligned} \end{aligned}$$

We denote that two marginals by

4.16 $$\begin{aligned} \rho ^1_\varepsilon = {{\,\textrm{proj}\,}}^1_\# {\bar{\gamma }}_\varepsilon , \qquad \rho ^2_\varepsilon = {{\,\textrm{proj}\,}}^2_\# {\bar{\gamma }}_\varepsilon . \end{aligned}$$

The next proposition collects the properties of ${\bar{\gamma }}_\varepsilon$. First, it shows that its marginals are quantitatively below $\rho$, a property which crucially relies on the presence of $\tau$ in the definition of ${\bar{\gamma }}_\varepsilon$ and on the regularity of $\rho$ and *V*. Second, it proves that this is still a recovery sequence (without prescribed marginals) as the one build in the proof of Theorem 1.2 was.

#### Proposition 4.2

Under the same assumptions of Theorem 1.3, let ${\bar{\gamma }}_\varepsilon$ be as defined in (4.14), with ${\bar{\gamma }}_\varepsilon$ constructed with parameter *H*. Then,

4.17 $$\begin{aligned} \lim _{\varepsilon \rightarrow 0} E_\varepsilon (\sqrt{{\bar{\gamma }}_\varepsilon }) \le \frac{1}{2} \int _{\Omega _H} {{\,\textrm{tr}\,}}\left( \sqrt{\nabla ^2V(x, T(x))}\right) \rho (x) \textrm{d}x . \end{aligned}$$

Moreover, there exists $0<c_H<1$ such that, for $\varepsilon$ sufficiently small,

4.18 $$\begin{aligned} \rho ^i_\varepsilon (x) \le \rho (x) - c_H \tau \qquad \text {for } i=1,2 \text { and } x\in \Omega _H', \end{aligned}$$

and

4.19 $$\begin{aligned} (\rho - \rho ^i_\varepsilon )(\mathbb {R}) = 1 - {\bar{\gamma }}_\varepsilon (\mathbb {R}^2) = \rho (\Omega _H^c) + \tau |{\Omega _H}| . \end{aligned}$$

#### Proof

We use here the notation introduced in Sects.  3 and  4.

*Step 1: proof of* (4.18). Let us deal first with the case of $\rho ^1_\varepsilon$. First of all, notice that ${{\,\textrm{supp}\,}}\rho ^1_\varepsilon \subset \Omega _H'$ and $\rho >\tau$ on $\Omega _H'$.

We have that

4.20 $$\begin{aligned} \rho ^1_\varepsilon (x) = \sum _i \Bigl (\bigl [(\rho -\tau ) {\varvec{1}}_{I^1_i}\bigr ]*\eta ^1_i\Bigr )(x), \end{aligned}$$

where $\eta ^1_i = {{\,\textrm{proj}\,}}^1_\# \Gamma _{M_{\varepsilon ,\beta }(x_i),N}$. For every *x* there can be at most two non zero terms contributing to the sum, because the convolutions kernels $\eta ^1_i$ have support with diameter ${{\,\textrm{diam}\,}}({{\,\textrm{supp}\,}}\eta ^1_i) \le C\beta ^{1/2}N^{1/2}$, whereas two non-consecutive intervals are further away than $\textsf{d}(I^1_{i-1},I^1_{i+1}) \ge C \delta \gg \beta ^{1/2}N^{1/2}$, thanks to (4.10).

If $\textsf{d}\bigl (x,(I^1_i)^c\bigr )\ge C\beta ^{1/2}N^{1/2}$, there is actually only the *i*-th term in the sum. Since the functions $(\rho -\tau ) {\varvec{1}}_{I^1_i}$ and $(\rho -\tau )$ coincide in $B(x,C\beta ^{1/2}N^{1/2})$, when we convolve them with $\eta ^1_i$ they give the same value at *x*, and therefore we can compute that

$$\begin{aligned} \rho ^1_\varepsilon (x)= &   \Bigl (\bigl [(\rho -\tau ) {\varvec{1}}_{I^1_i}\bigr ] * \eta ^1_i\Bigr )(x) = \Bigl ((\rho -\tau ) * \eta ^1_i\Bigr )(x)\\\le &   \rho (x) - \tau + C {{\,\textrm{Lip}\,}}\left( \rho |_{\Omega _H'}\right) \beta ^{1/2} N^{1/2}. \end{aligned}$$

Otherwise, let *x* be a point where the two non zero terms in the sum (4.20) are $i-1$ and *i*. Then, since the functions ${\varvec{1}}_{I^1_{i-1}\cup I^1_i}$ and 1 coincide in $B(x,C\beta ^{1/2}N^{1/2})$, we have

$$\begin{aligned} \begin{aligned} \rho ^1_\varepsilon (x)&= \Bigl (\bigl [(\rho -\tau ) {\varvec{1}}_{I^1_{i-1}}\bigr ] * \eta ^1_{i-1}\Bigr )(x) + \Bigl (\bigl [(\rho -\tau ) {\varvec{1}}_{I^1_i}\bigr ] * \eta ^1_i\Bigr )(x) \\&= \Bigl (\bigl [(\rho -\tau ) {\varvec{1}}_{I^1_{i-1}\cup I^1_i}\bigr ] * \eta ^1_{i-1}\Bigr )(x) + \Bigl (\bigl [(\rho -\tau ) {\varvec{1}}_{I^1_i}\bigr ] * (\eta ^1_i-\eta ^1_{i-1})\Bigr )(x) \\&= \Bigl ((\rho -\tau ) * \eta ^1_{i-1}\Bigr )(x) + \Bigl (\bigl [(\rho -\tau ) {\varvec{1}}_{I^1_i}\bigr ] * (\eta ^1_i-\eta ^1_{i-1})\Bigr )(x) \\&\le \rho (x) - \tau + C {{\,\textrm{Lip}\,}}\left( \rho |_{\Omega _H'}\right) \beta ^{1/2} N^{1/2} + \Vert {\rho }\Vert _\infty \Vert {\eta ^1_i-\eta ^1_{i-1}}\Vert _1 \\&\le \rho (x) - \tau + C L \beta ^{1/2} N^{1/2} + C_H \varepsilon ^{-1/4}\delta ^2 + C_H \varepsilon ^{-1/2}\beta \delta \end{aligned} \end{aligned}$$

because $\Vert {\eta ^1_i-\eta ^1_{i-1}}\Vert _1 \le \Vert {\Gamma _{M_{\varepsilon ,\beta }(x_i),N} - \Gamma _{M_{\varepsilon ,\beta }(x_{i-1}),N}}\Vert _1 \le C_H \varepsilon ^{-1/4}\delta ^2 + C_H \varepsilon ^{-1/2}\beta \delta$ by Lemma 4.3 (with ${{\varvec{x}}}_1={{\varvec{x}}}_2=0$). Therefore (4.18) follows (with any $c_H<1$) because, from (4.8)–(4.11), we have $\tau \gg \varepsilon ^{-1/2}\beta \delta N \gg \varepsilon ^{-1/2}\beta \delta$, $\tau \gg \beta ^{1/2}N^{1/2}$ and $\tau \gg \varepsilon ^{-1/4}\delta ^2$.

Let us now deal with the case of $\rho ^2_\varepsilon$. From the definitions (4.14)–(4.16), we have

$$\begin{aligned} \begin{aligned} \rho ^2_\varepsilon (y)&= \sum _i {{\,\textrm{proj}\,}}^2_\# {\bar{\gamma }}_{\varepsilon ,i}(y) = \sum _i {{\,\textrm{proj}\,}}^2_\# \left( (\textrm{Id},T_\delta )_\#\bigl ((\rho -\tau ){\varvec{1}}_{I^1_i}\bigr ) * \Gamma _{M_{\varepsilon ,\beta }(x_i),N} \right) (y) \\&= \sum _i \left( {{\,\textrm{proj}\,}}^2_\# (\textrm{Id},T_\delta )_\# \bigl ((\rho -\tau ){\varvec{1}}_{I^1_i}\bigr )\right) * \left( {{\,\textrm{proj}\,}}^2_\# \Gamma _{M_{\varepsilon ,\beta }(x_i),N}\right) (y) \\&= \sum _i \left( \bigl [{T_\delta }_\# \bigl ((\rho -\tau ){\varvec{1}}_{I^1_i}\bigr )\bigr ] * \eta ^2_i \right) (y) = \sum _i \Bigl (\bigl [{T_\delta }_\#(\rho -\tau ) {\varvec{1}}_{I^2_i}\bigr ]*\eta ^2_i\Bigr )(y), \end{aligned} \end{aligned}$$

where $\eta ^2_i = {{\,\textrm{proj}\,}}^2_\# \Gamma _{M_{\varepsilon ,\beta },N}$. For $y\in \Omega _H$ we have

$$\begin{aligned} \begin{aligned} |{{T_\delta }_\#\rho (y) - \rho (y)}|&= |{{T_\delta }_\#\rho (y) - T_\#\rho (y)}| = \left|{\frac{\rho \bigl (T_\delta ^{-1}(y)\bigr )}{T_\delta '\bigl (T_\delta ^{-1}(y)\bigr )} - \frac{\rho \bigl (T^{-1}(y)\bigr )}{T'\bigl (T^{-1}(y)\bigr )}}\right| \\&\le \frac{|{\rho \bigl (T_\delta ^{-1}(y)\bigr )-\rho \bigl (T^{-1}(y)\bigr )}|}{T'\bigl (T^{-1}(y)\bigr )}\\&\quad + \rho \bigl (T_\delta ^{-1}(y)\bigr ) \frac{|{T_\delta '\bigl (T_\delta ^{-1}(y)\bigr )-T'\bigl (T^{-1}(y)\bigr )}|}{T_\delta '\bigl (T_\delta ^{-1}(y)\bigr )T'\bigl (T^{-1}(y)\bigr )} \\&\le {{\,\textrm{Lip}\,}}(\rho |_{\Omega _H'})C\delta L + L {{\,\textrm{Lip}\,}}(T'|_{\Omega _H'})C\delta L^2 \le C_H \delta , \end{aligned} \end{aligned}$$

where we used (4.7) to bound the norms of $\rho$ and $T'$ and the Lipschitz constants. In particular, if $y\in I^2_i$, then ${T_\delta }_\#(\rho -\tau )(y) = {T_\delta }_\#\rho (y)-\tau /T'(x_i) \le \rho (y) + C_H\delta - \tau /L$.

We have, therefore, that

$$\begin{aligned} \rho ^2_\varepsilon (y) \le \sum _i \Bigl (\bigl [\bigl (\rho -\tau /L+C_H\delta \bigr ) {\varvec{1}}_{I^2_i}\bigr ] * \eta ^2_i\Bigr )(y). \end{aligned}$$

We observe the similarity with (4.20), where $\rho -\tau$, $I^1_i$ and $\eta ^1_i$ have been replaced by $\rho -\tau /L+C_H\delta$, $I^2_i$ and $\eta ^2_i$ respectively. We have ${{\,\textrm{diam}\,}}(I^2_i) \ge C\delta /L \gg \beta ^{1/2}N^{1/2}$ and ${{\,\textrm{diam}\,}}({{\,\textrm{supp}\,}}\eta ^2_i)\le C\beta ^{1/2}N^{1/2}$, so with the same argument as before we obtain

$$\begin{aligned} \rho ^2_\varepsilon (y) \le \rho (y) - \tau /L + C_H\bigl ( \delta + \beta ^{1/2}N^{1/2} + \varepsilon ^{-1/4}\delta ^2 + \varepsilon ^{-1/2}\beta \delta \bigr ), \end{aligned}$$

from which the thesis follows with any $c_H<1/L(H)$ because $\tau$ is asymptotically larger than all the error terms.

*Step 2: proof of* (4.19).

Since $\Gamma _{M_{\varepsilon ,\beta }(x_i),N}^{(x,T_\delta (x))}$ is a probability measure, we have

$$\begin{aligned} \begin{aligned} {\bar{\gamma }}_\varepsilon (\mathbb {R}^2)&= \sum _i \int _\mathbb {R}\int _{I^1_i} \Gamma _{M_{\varepsilon ,\beta }(x_i),N}^{(x,T_\delta (x))}({{\varvec{x}}}') (\rho (x)-\tau )\textrm{d}{{\varvec{x}}}' \\&= \sum _i \int _{I^1_i} (\rho (x)-\tau ) \textrm{d}x = \int _{\Omega _H} (\rho (x)-\tau ) \textrm{d}x = \rho (\Omega _H) - \tau |{\Omega _H}| , \end{aligned} \end{aligned}$$

from which

$$\begin{aligned} 1 - {\bar{\gamma }}_\varepsilon (\mathbb {R}^2) = 1 - [\rho (\Omega _H) - \tau |{\Omega _H}|] = \rho (\Omega _H^c) + \tau |{\Omega _H}| . \end{aligned}$$

*Step 3*.

For every *i* and $x\in I^1_i$,

we claim the estimate

4.21 $$\begin{aligned} \begin{aligned} E_\varepsilon&\left( \sqrt{\Gamma ^{(x,T_\delta (x))}_{M_{\varepsilon ,\beta }(x_i),N}}\right) - E_\varepsilon \left( \sqrt{\Gamma _{M_{\varepsilon ,\beta }(x),N}^{(x,T(x))}}\right) \\&\le C_H (\varepsilon ^{-3/4}\delta ^2 + \varepsilon ^{-1}\beta \delta ) \bigl ( \delta ^4 + \varepsilon ^{1/2}N + \beta N \delta ^2 \bigr ) + C_H \varepsilon ^{1/2} e^{-N/2} + C_H\delta . \end{aligned} \end{aligned}$$

Notice that the right hand side of (4.21) goes to 0. In fact, thanks to (4.10) and the fact that $N\rightarrow \infty$ we have

$$\begin{aligned} \beta N\delta ^2 \ll \delta ^4 \ll \varepsilon ^{1/2} \ll \varepsilon ^{1/2}N, \end{aligned}$$

therefore, thanks to (4.8)–(4.10), the right hand side of (4.21) is less than

$$\begin{aligned} \begin{aligned}&C_H(\varepsilon ^{-3/4}\delta ^2 + \varepsilon ^{-1}\beta \delta ) \varepsilon ^{1/2}N + C_H\varepsilon ^{1/2}e^{-N/2} + C_H\delta \\&\quad \le C_H(\varepsilon ^{-1/4}\delta ^2 + \varepsilon ^{-1/2}\beta \delta )N + C_H\varepsilon ^{1/2}e^{-N/2} + C_H\delta \\&\quad \ll N |{\log \varepsilon }|^{-2} + \varepsilon ^{1/40}N^{2/5} + e^{-N/2} + \delta \ll 1 . \end{aligned} \end{aligned}$$

We observe that by (4.13) the points ${{\varvec{x}}}_1=\bigl (x,T_\delta (x)\bigr ),{{\varvec{x}}}_2=\bigl (x,T(x)\bigr ) \in \mathbb {R}^2$ and the symmetric matrices $A_1=\sqrt{\nabla ^2V\bigl (x_i,T(x_i)\bigr )}$, $A_2=\sqrt{\nabla ^2V({{\varvec{x}}}_2)}$ which generate $M_1=M_{\varepsilon ,\beta }(x_i)$ and $M_2=M_{\varepsilon ,\beta }(x)$ satisfy

4.22 $$\begin{aligned} |{{{\varvec{x}}}_1-{{\varvec{x}}}_2}| \le L\delta ^2 \qquad \text {and} \qquad |{A_1-A_2}| \le C_H\delta . \end{aligned}$$

In fact, $\bigl (x_i,T(x_i)\bigr )$ and $\bigl (x,T(x)\bigr )$ belong to a region of ${{\,\textrm{graph}\,}}(T)$ where $\nabla ^2 V$ is Lipschitz. As a consequence,

4.23 $$\begin{aligned} |{M_1-M_2}| = \left|{\left( \frac{A_1}{\varepsilon ^{1/2}}-\frac{I}{\beta }\right) -\left( \frac{A_2}{\varepsilon ^{1/2}}-\frac{I}{\beta }\right) }\right| = \varepsilon ^{-1/2} |{A_1-A_2}| \le C_H \varepsilon ^{-1/2}\delta . \end{aligned}$$

As regards the potential energy, denoting by *E* the union of the supports of the probability measures appearing in (4.21)

$$\begin{aligned} E = {{\,\textrm{supp}\,}}\Gamma ^{(x,T_\delta (x))}_{M_{\varepsilon ,\beta }(x_i),N} \cup {{\,\textrm{supp}\,}}\Gamma _{M_{\varepsilon ,\beta }(x),N}^{(x,T(x))} \end{aligned}$$

we have

4.24 $$\begin{aligned} \begin{aligned} \int _{\mathbb {R}^2}&V({{\varvec{x}}}') \Gamma ^{(x,T_\delta (x))}_{M_{\varepsilon ,\beta }(x_i),N} ({{\varvec{x}}}') \textrm{d}{{\varvec{x}}}' - \int _{\mathbb {R}^2} V({{\varvec{x}}}') \Gamma _{M_{\varepsilon ,\beta }(x),N}^{(x,T(x))}({{\varvec{x}}}') \textrm{d}{{\varvec{x}}}' \\&\le \left( \sup _E V\right) \int _{\mathbb {R}^2} \left|{\Gamma ^{(x,T_\delta (x))}_{M_{\varepsilon ,\beta }(x_i),N} ({{\varvec{x}}}') \textrm{d}{{\varvec{x}}}' - \Gamma _{M_{\varepsilon ,\beta }(x),N}^{(x,T(x))}({{\varvec{x}}}')}\right| \textrm{d}{{\varvec{x}}}' \end{aligned} \end{aligned}$$

Observe that, by Lemma 4.3, we have

4.25 $$\begin{aligned} \int \Big |\Gamma ^{(x,T_\delta (x))}_{M_{\varepsilon ,\beta }(x_i),N}({{\varvec{x}}}') d{{\varvec{x}}}' - \Gamma _{M_{\varepsilon ,\beta }(x),N}^{(x,T(x))}({{\varvec{x}}}')\Big | d{{\varvec{x}}}' \le C_H \varepsilon ^{-1/4}\delta ^2 + C_H \varepsilon ^{-1/2}\beta \delta . \end{aligned}$$

We claim that *E* is contained in a strip of size $C (\delta ^2 + \varepsilon ^{1/4}N^{1/2} + \beta ^{1/2} N^{1/2} \delta {{\,\textrm{Lip}\,}}(T'))$ around the graph of the affine map which equals $T_\delta$ in $I^1_i$

4.26 $$\begin{aligned} E \subseteq S= \{ {{\varvec{x}}}\in \mathbb {R}^2 : \textsf{d}\bigl ({{\varvec{x}}}, {{\,\textrm{graph}\,}}(T_\delta )\bigr ) \le C (\delta ^2 + \varepsilon ^{1/4}N^{1/2} + \beta ^{1/2} N^{1/2} \delta {{\,\textrm{Lip}\,}}(T'))\}. \end{aligned}$$

Indeed, by construction ${{\,\textrm{supp}\,}}\Gamma ^{(x,T_\delta (x))}_{M_{\varepsilon ,\beta }(x_i),N}$ is a rectangle centered on the graph of $T_\delta$ and with the longer side aligned with the graph of $T_\delta$. Moreover, its width is $\varepsilon ^{1/4}N^{1/2}$; hence the claim (4.26) is proved for the first rectangle in the definition of *E*.

On the other side, we observe that the rectangle ${{\,\textrm{supp}\,}}\Gamma _{M_{\varepsilon ,\beta }(x),N}^{(x,T(x))}$ is centered in the point (*x*, *T*(*x*)), whose distance from the graph of $T_\delta$ is estimated by $\Vert T'' \Vert _{L^\infty } \delta ^2$ by (4.13); moreover, this rectangle is tilted with respect to the previous one proportionally to $|T'(x)-T'_\delta (x)| \le \delta {{\,\textrm{Lip}\,}}(T')$, which in turn gives a contribution of $\beta ^{1/2} N^{1/2} \delta {{\,\textrm{Lip}\,}}(T')$. This proves (4.26).

Since for every *x* the point (*x*, *T*(*x*)) and the point are at distance at most $(x,T_\delta (x))$ $\Vert T'' \Vert _{L^\infty } \delta ^2$ by (4.13), from (4.26) we deduce that *S* is contained in the strip of width $C (1+\Vert T'' \Vert _{L^\infty } ) (\delta ^2 + \varepsilon ^{1/4}N^{1/2} + \beta ^{1/2} N^{1/2} \delta )$ around the graph of *T*. Hence, recalling that both *V* and $\nabla V$ vanish along the graph of *T*, and more precisely recalling that *V* has uniform quadratic growth around the graph of *T* has stated in we obtain that

4.27 $$\begin{aligned} \sup _E V \le C (1+\Vert T'' \Vert _{L^\infty } )^2 (\delta ^2 + \varepsilon ^{1/4}N^{1/2} + \beta ^{1/2} N^{1/2} \delta )^2. \end{aligned}$$

Hence we deduce by (4.24), (4.25), and (4.27) that

4.28 $$\begin{aligned} \begin{aligned} \int V({{\varvec{x}}}')&\Gamma ^{(x,T_\delta (x))}_{M_{\varepsilon ,\beta }(x_i),N} ({{\varvec{x}}}') \textrm{d}{{\varvec{x}}}' - \int V({{\varvec{x}}}') \Gamma _{M_{\varepsilon ,\beta }(x),N}^{(x,T(x))}({{\varvec{x}}}') \textrm{d}{{\varvec{x}}}' \\&\le C_H (\varepsilon ^{-1/4}\delta ^2 + \varepsilon ^{-1/2}\beta \delta ) (\delta ^2 + \varepsilon ^{1/4}N^{1/2} + \beta ^{1/2} N^{1/2} \delta )^2 \\&\le C_H (\varepsilon ^{-1/4}\delta ^2 + \varepsilon ^{-1/2}\beta \delta ) (\delta ^4 + \varepsilon ^{1/2}N + \beta N \delta ^2). \end{aligned} \end{aligned}$$

In view of (4.28), we deduce the corresponding statement to (4.21) for the potential energy.

As regards the kinetic energy, defined by (3.6), we observe that it equals

$$\begin{aligned} \begin{aligned} {{\,\textrm{KE}\,}}(\Gamma _{M_1,N}^{{{\varvec{x}}}_1}) - {{\,\textrm{KE}\,}}(\Gamma _{M_2,N}^{{{\varvec{x}}}_2})&= {{\,\textrm{KE}\,}}(\Gamma _{M_1,\infty }^{{{\varvec{x}}}_1}) - {{\,\textrm{KE}\,}}(\Gamma _{M_2,\infty }^{{{\varvec{x}}}_2})\\&\quad + [{{\,\textrm{KE}\,}}(\Gamma _{M_1,N}^{{{\varvec{x}}}_1}) - {{\,\textrm{KE}\,}}(\Gamma _{M_1,\infty }^{{{\varvec{x}}}_1})] \\&\quad - [{{\,\textrm{KE}\,}}(\Gamma _{M_2,N}^{{{\varvec{x}}}_2}) - {{\,\textrm{KE}\,}}(\Gamma _{M_2,\infty }^{{{\varvec{x}}}_2})] . \end{aligned} \end{aligned}$$

The first difference is

$$\begin{aligned} {{\,\textrm{KE}\,}}(\Gamma _{M_1,\infty }^{{{\varvec{x}}}_1}) - {{\,\textrm{KE}\,}}(\Gamma _{M_2,\infty }^{{{\varvec{x}}}_2}) = \frac{{{\,\textrm{tr}\,}}M_1}{4} - \frac{{{\,\textrm{tr}\,}}M_2}{4} = \frac{q_1-q_2}{4\varepsilon ^{1/2}} \le \frac{\delta }{4\varepsilon ^{1/2}} \end{aligned}$$

because, by the variational characterization of the largest eigenvalue, we have

$$\begin{aligned} q_1 = \max _{|{v}|\le 1}v^TA_1v \le \max _{|{v}|\le 1}v^TA_2v + \max _{|{v}|\le 1}v^T(A_1-A_2)v \le q_2 + C_H \delta . \end{aligned}$$

Using (3.9) for the two terms in square brackets, we get

$$\begin{aligned} {{\,\textrm{KE}\,}}(\Gamma _{M_1,N}^{{{\varvec{x}}}_1}) - {{\,\textrm{KE}\,}}(\Gamma _{M_2,N}^{{{\varvec{x}}}_2}) \le C({{\,\textrm{tr}\,}}M_1 + {{\,\textrm{tr}\,}}M_2) e^{-N/2} + \frac{C_H\delta }{\varepsilon ^{1/2}}\le C_H e^{-N/2} + \frac{C_H\delta }{\varepsilon ^{1/2}} . \end{aligned}$$

*Step 4*. We conclude the proof. By convexity of the kinetic energy, since the potential energy is linear and thanks to (4.21), with the remark following it, we have that

$$\begin{aligned} \begin{aligned} \limsup _{\varepsilon \rightarrow 0} E_\varepsilon (\sqrt{{\bar{\gamma }}_\varepsilon })&\le \limsup _{\varepsilon \rightarrow 0} \sum _{i=1} \int _{I^1_i} E_\varepsilon \left( \sqrt{\Gamma _{M_{\varepsilon ,\beta }(x_i),N}^{(x,T_\delta (x))}}\right) \rho (x) \textrm{d}x \\&\le \limsup _{\varepsilon \rightarrow 0} \int _{\Omega _H} E_\varepsilon \left( \sqrt{\Gamma _{M_{\varepsilon ,\beta }(x),N}^{(x,T(x))})}\right) \rho (x) \textrm{d}x . \end{aligned} \end{aligned}$$

We now estimate the integrand in the right-hand side with ${{\,\textrm{tr}\,}}\left( \sqrt{\nabla ^2V(x,T(x))}\right) \rho (x)$ up to small errors. To this end, we bound the kinetic energy by passing to the non-truncated Gaussians with the estimate (3.9) and by the exact computation (3.7). We obtain

$$\begin{aligned} \begin{aligned}&\varepsilon ^{1/2} KE\left( \sqrt{\Gamma _{M_{\varepsilon ,\beta }(x),N}^{(x,T(x))})}\right) = \varepsilon ^{1/2}{{\,\textrm{KE}\,}}\left( \sqrt{\Gamma _{M_{\varepsilon ,\beta }(x),N}}\right) \\&\quad \le \varepsilon ^{1/2}{{\,\textrm{KE}\,}}\left( \sqrt{\Gamma _{M_{\varepsilon ,\beta }(x),\infty }}\right) + C \varepsilon ^{1/2} {{\,\textrm{tr}\,}}\bigl (M_{\varepsilon ,\beta }(x)\bigr ) e^{-N/2} \\&\quad = \varepsilon ^{1/2}\frac{{{\,\textrm{tr}\,}}M_{\varepsilon ,\beta }(x)}{4} + Ce^{-N/2} = \frac{1}{4}{{\,\textrm{tr}\,}}\left( \sqrt{\nabla ^2V(x,T(x))}\right) + \frac{\varepsilon ^{1/2}}{2\beta } + Ce^{-N/2}. \end{aligned} \end{aligned}$$

In order to estimate the potential energy, we need to compare the *V* with its second order Taylor expansion. Since $\nabla ^2V$ is locally Lipschitz in a neighborhood of ${{\,\textrm{graph}\,}}T$, there is $r>0$ such that for every ${{\varvec{x}}}\in {{\,\textrm{supp}\,}}\gamma \cap \Omega _H^2$ and ${{\varvec{y}}}\in B_r(0)$ we have

$$\begin{aligned} \left|{V({{\varvec{x}}}+{{\varvec{y}}}) - \frac{1}{2}\left|{\sqrt{\nabla ^2V({{\varvec{x}}})}{{\varvec{y}}}}\right|^2}\right| \le \Vert {\nabla ^3V}\Vert _{L^\infty } \cdot |{{{\varvec{y}}}}|^3 \le C_H |{{{\varvec{y}}}}|^3, \end{aligned}$$

where the $L^\infty$ norm is taken in a neighborhood of size $(\beta N)^{1/2}$ of ${{\,\textrm{supp}\,}}\gamma \cap \Omega _H^2$. Then, thanks to (3.8)^2^ and (3.7), and since $|{{\varvec{y}}}| \le (\beta N)^{1/2}$ for ${{\varvec{y}}}\in {{\,\textrm{supp}\,}}\Gamma _{M_{\varepsilon ,\beta }(x),N}$, we have

$$\begin{aligned} \begin{aligned}&\varepsilon ^{-1/2}\int V({{\varvec{y}}}) \Gamma _{M_{\varepsilon ,\beta }(x),N}^{(x,T(x))}({{\varvec{y}}})\, d{{\varvec{y}}}\\&\quad \le \frac{\varepsilon ^{-1/2}}{2} \int _{\mathbb {R}^2} \left|{\sqrt{\nabla ^2V(x,T(x))}{{\varvec{y}}}}\right|^2 \Gamma _{M_{\varepsilon ,\beta }(x),N}({{\varvec{y}}}) \textrm{d}{{\varvec{y}}}\\&\qquad + C_H \varepsilon ^{-1/2} \int _{\mathbb {R}^2} |{{\varvec{y}}}|^3 \Gamma _{M_{\varepsilon ,\beta }(x),N}({{\varvec{y}}}) \textrm{d}{{\varvec{y}}}\\&\quad \le \frac{\varepsilon ^{-1/2}}{2} \int _{\mathbb {R}^2} \left|{\sqrt{\nabla ^2V(x,T(x))}{{\varvec{y}}}}\right|^2 \Gamma _{M_{\varepsilon ,\beta }(x),\infty }({{\varvec{y}}}) \textrm{d}{{\varvec{y}}}\\&\qquad + C \varepsilon ^{-1/2} N e^{-N/2} + C_H \varepsilon ^{-1/2} \beta ^{3/2} N^{3/2} \\&\quad \le \frac{1}{4} {{\,\textrm{tr}\,}}\left( \sqrt{\nabla ^2V(x,T(x))}\right) + C \varepsilon ^{-1/2} N e^{-N/2} + C_H \varepsilon ^{-1/2} \beta ^{3/2} N^{3/2} . \end{aligned} \end{aligned}$$

Overall, from the estimates in Step 4 we obtain that

$$\begin{aligned} \begin{aligned} E_\varepsilon (\sqrt{{\bar{\gamma }}_\varepsilon })&\le \frac{1}{2} \int _{\Omega _H} {{\,\textrm{tr}\,}}\left( \sqrt{\nabla ^2V(x,T(x))}\right) \rho (x) \textrm{d}x \\&\quad +C_H \left( \frac{\varepsilon ^{1/2}}{\beta }+ Ce^{-N/2} + C \varepsilon ^{-1/2} N e^{-N/2} + C_H \varepsilon ^{-1/2} \beta ^{3/2} N^{3/2} \right) , \end{aligned} \end{aligned}$$

which concludes the proof of (4.17) since by (4.8) and (4.9) we have

$$\begin{aligned} \begin{aligned}&\frac{\varepsilon ^{1/2}}{\beta }+ Ce^{-N/2} + C \varepsilon ^{-1/2}N\varepsilon ^{-1/2} N e^{-N/2} + C_H \varepsilon ^{-1/2} \beta ^{3/2} N^{3/2} \\&\quad \ll \frac{1}{N} + C e^{-N/2} + \varepsilon ^{1/10}N^{3/2} \ll 1 . \end{aligned} \end{aligned}$$

As a side note, it could be easily seen by means of the kind of computations performed in this proof that (4.17) is actually an equality, but since we will not need this in the sequel we do not pursue this matter here. $\square$

#### Lemma 4.3

Let ${{\varvec{x}}}_1,{{\varvec{x}}}_2\in \mathbb {R}^2$ with $|{{{\varvec{x}}}_1-{{\varvec{x}}}_2}|\le C_0\delta ^2$ and let $A_1,A_2$ be two degenerate positive-semidefinite symmetric $2\times 2$ matrices with $|{A_i}|\le L$ and $|{A_1-A_2}|\le C_0\delta$; finally let $M_i=M_{\varepsilon ,\beta }(A_i)=\frac{A_i}{\varepsilon ^{1/2}}+\frac{I}{\beta }$. Then there exists a positive constant $C_H$ only depending on *H* and $C_0$ such that

$$\begin{aligned} \Vert {\Gamma _{M_1,N}^{{{\varvec{x}}}_1}-\Gamma _{M_2,N}^{{{\varvec{x}}}_2}}\Vert _1 \le C_H \varepsilon ^{-1/4}\delta ^2 + C_H \varepsilon ^{-1/2}\beta \delta . \end{aligned}$$

Observe that, under the assumptions (4.8)–(4.10), the right hand side goes to 0 when $\varepsilon \rightarrow 0$.

#### Proof

Thanks to (3.4) we have

$$\begin{aligned} \Vert {\Gamma _{M_1,N}^{{{\varvec{x}}}_1}-\Gamma _{M_2,N}^{{{\varvec{x}}}_2}}\Vert _1 \le \Vert {\Gamma _{M_1,\infty }^{{{\varvec{x}}}_1}-\Gamma _{M_2,\infty }^{{{\varvec{x}}}_2}}\Vert _1 + CNe^{-N/2}. \end{aligned}$$

Notice that, by (4.8)–(4.10), $Ne^{-N/2} \ll N^2\varepsilon ^{1/4}=\varepsilon ^{1/2}N^2\varepsilon ^{-1/4}\ll \beta N\varepsilon ^{-1/4}\ll \delta ^2\varepsilon ^{-1/4}$, so we can forget about this error term because it is dominated by the right hand side of the thesis.

We split the estimate into first changing only the center point, and then changing the matrix. By the triangle inequality we have

4.29 $$\begin{aligned} \Vert {\Gamma _{M_1,\infty }^{{{\varvec{x}}}_1}-\Gamma _{M_2,\infty }^{{{\varvec{x}}}_2}}\Vert _1 \le \Vert {\Gamma _{M_1,\infty }^{{{\varvec{x}}}_1}-\Gamma _{M_1,\infty }^{{{\varvec{x}}}_2}}\Vert _1 + \Vert {\Gamma _{M_1,\infty }^{{{\varvec{x}}}_2}-\Gamma _{M_2,\infty }^{{{\varvec{x}}}_2}}\Vert _1 . \end{aligned}$$

The first norm can be estimated in the following way. If *a* and *b* are the eigenvalues of $M_1$, we have $\nabla \Gamma _{M_1,\infty }(w,z) = 2 (aw,bz)^T \Gamma _{M_1,\infty }(w,z)$, therefore

$$\begin{aligned} \begin{aligned} \Vert {\nabla \Gamma _{M_1,\infty }}\Vert _1&\le G_{M_1,\infty }^{-1} \int _{\mathbb {R}^2} (2a|{w}|+2b|{z}|)e^{-aw^2-bz^2} \textrm{d}w \textrm{d}z \\&= \frac{\sqrt{ab}}{\pi }\left( \frac{2\sqrt{\pi }}{\sqrt{b}}+\frac{2\sqrt{\pi }}{\sqrt{a}}\right) = \frac{2}{\sqrt{\pi }}(\sqrt{a}+\sqrt{b})\\&\le C q(x)^{1/2} \varepsilon ^{-1/4} \le C_H \varepsilon ^{-1/4} . \end{aligned} \end{aligned}$$

From this we get

$$\begin{aligned} \begin{aligned} \Vert {\Gamma _{M_1,\infty }^{{{\varvec{x}}}_1}-\Gamma _{M_1,\infty }^{{{\varvec{x}}}_2}}\Vert _1&= \int _{\mathbb {R}^2} |{\Gamma _{M_1,\infty }({{\varvec{y}}}+{{\varvec{x}}}_2-{{\varvec{x}}}_1)-\Gamma _{M_1,\infty }({{\varvec{y}}})}| \textrm{d}{{\varvec{y}}}\\&= \int _{\mathbb {R}^2} \left|{\int _0^1 \nabla \Gamma _{M_1,\infty }\bigl ({{\varvec{y}}}+s({{\varvec{x}}}_2-{{\varvec{x}}}_1)\bigr ) \cdot ({{\varvec{x}}}_2-{{\varvec{x}}}_1) \textrm{d}s}\right| \textrm{d}{{\varvec{y}}}\\&\le \int _{\mathbb {R}^2} \int _0^1 \left|{\nabla \Gamma _{M_1,\infty }\bigl ({{\varvec{y}}}+s({{\varvec{x}}}_2-{{\varvec{x}}}_1)\bigr )}\right| \cdot |{{{\varvec{x}}}_2-{{\varvec{x}}}_1}| \textrm{d}s \textrm{d}{{\varvec{y}}}\\&\le C\delta ^2 \Vert {\nabla \Gamma _{M_1,\infty }}\Vert _1 \le C_H \delta ^2 \varepsilon ^{-1/4}. \end{aligned} \end{aligned}$$

Let’s now turn to the second norm in (4.29). For $t\in [0,1]$, define the matrix $B_t=M_1+t(M_2-M_1)$. Notice that

$$\begin{aligned} B_t = \frac{1}{\varepsilon ^{1/2}}\left( A_1+\frac{\varepsilon ^{1/2}}{\beta }I+t(A_2-A_1)\right) \end{aligned}$$

and $|{A_2-A_1}|<\delta \ll \varepsilon ^{1/2}/\beta$, because $\beta \delta \ll \varepsilon ^{2/5+1/8}N^{-3/5}\ll \varepsilon ^{21/40}\ll \varepsilon ^{1/2}$ by (4.9)–(4.10). Therefore we have that for $\varepsilon$ small $1/2\det M_1 \le \det B_t \le 2\det M_1$ and $1/2{{\,\textrm{tr}\,}}M_1 \le {{\,\textrm{tr}\,}}B_t \le 2{{\,\textrm{tr}\,}}M_1$. Moreover,

$$\begin{aligned} \begin{aligned} \left|{\frac{\textrm{d}}{\textrm{d}t}\log \det B_t}\right|&= \frac{1}{\det B_t}\left|{{{\,\textrm{tr}\,}}\left( {{\,\textrm{adj}\,}}(B_t) \frac{\textrm{d}B_t}{\textrm{d}t}\right) }\right| = \frac{\left|{{{\,\textrm{tr}\,}}\bigl ({{\,\textrm{adj}\,}}(B_t)(M_2-M_1)\bigr )}\right|}{\det B_t} \\&\le C_H \frac{|{B_t}| \cdot |{M_2-M_1}|}{\det B_t} \le C_H \frac{\varepsilon ^{-1/2}\cdot \delta \varepsilon ^{-1/2}}{\varepsilon ^{-1/2}\beta ^{-1}} = C_H \frac{\beta \delta }{\varepsilon ^{1/2}} \end{aligned} \end{aligned}$$

and

$$\begin{aligned} \begin{aligned} \frac{\textrm{d}}{\textrm{d}t}\Gamma _{B_t,\infty }({{\varvec{x}}})&= \frac{\textrm{d}}{\textrm{d}t} \left( \frac{\sqrt{\det B_t}}{\pi } e^{-{{\varvec{x}}}^TB_t{{\varvec{x}}}}\right) \\&= \frac{\frac{\textrm{d}}{\textrm{d}t}\det B_t}{2\pi \sqrt{\det B_t}} e^{-{{\varvec{x}}}^TB_t{{\varvec{x}}}} - {{\varvec{x}}}^T\frac{\textrm{d}B_t}{\textrm{d}t}{{\varvec{x}}}\frac{\sqrt{\det B_t}}{\pi } e^{-{{\varvec{x}}}^TB_t{{\varvec{x}}}} \\&= \frac{1}{2} \left( \frac{\textrm{d}}{\textrm{d}t}\log \det B_t\right) \Gamma _{B_t,\infty } - {{\varvec{x}}}^T(M_2-M_1){{\varvec{x}}}\Gamma _{B_t,\infty } . \end{aligned} \end{aligned}$$

Therefore,

$$\begin{aligned} \begin{aligned} \Vert {\Gamma _{M_1,\infty }^{{{\varvec{x}}}_2}-\Gamma _{M_2,\infty }^{{{\varvec{x}}}_2}}\Vert _1&= \Vert {\Gamma _{M_1,\infty }-\Gamma _{M_2,\infty }}\Vert _1 \le \int _0^1 \int _{\mathbb {R}^2} \left|{\frac{\textrm{d}}{\textrm{d}t}\Gamma _{B_t,\infty }({{\varvec{x}}})}\right| \textrm{d}{{\varvec{x}}}\textrm{d}t \\&\le C_H \int _0^1 \left|{\frac{\textrm{d}}{\textrm{d}t}\log \det B_t}\right| \int _{\mathbb {R}^2} \Gamma _{B_t,\infty } \textrm{d}{{\varvec{x}}}\textrm{d}t \\&\quad + \int _0^1 |{M_2-M_1}| \frac{\sqrt{\det B_t}}{\pi } \int _{\mathbb {R}^2} |{{{\varvec{x}}}}|^2 e^{-{{\varvec{x}}}^TB_t{{\varvec{x}}}}\textrm{d}{{\varvec{x}}}\textrm{d}t \\&= C_H \frac{\beta \delta }{\varepsilon ^{1/2}} + \frac{\delta }{\varepsilon ^{1/2}} \int _0^1 \frac{{{\,\textrm{tr}\,}}B_t}{2\det B_t} \textrm{d}t \\&\le C_H \frac{\beta \delta }{\varepsilon ^{1/2}} + C_H \frac{\delta }{\varepsilon ^{1/2}}\beta = C_H \frac{\beta \delta }{\varepsilon ^{1/2}}. \end{aligned} \end{aligned}$$

$$\square $$

## Deconvolution of plans

Let $\sigma ^1,\sigma ^2\in \mathscr {M}_+(\mathbb {R})$ with the same mass and $\Pi _0\in \Pi (\sigma ^1,\sigma ^2)$ be a transport plan. Fix a radial convolution kernel $\Theta \in C^\infty (\mathbb {R}^2;[0,1])$ with ${{\,\textrm{supp}\,}}\Theta \subset B(0,1)$. Define the rescaling $\Theta _\varepsilon (x)=\varepsilon ^{-1/2}\Theta (\varepsilon ^{-1/4}x)$ and the marginals $\theta (x)=({{\,\textrm{proj}\,}}^1_\#\Theta )(x)$, $\theta _\varepsilon (x)=({{\,\textrm{proj}\,}}^1_\#\Theta _\varepsilon )(x)=\varepsilon ^{-1/4}\theta (\varepsilon ^{-1/4}x)$. Define the convolved plan

$$\begin{aligned} \Pi _\varepsilon =\Pi _0*\Theta _\varepsilon . \end{aligned}$$

Let $\sigma ^i_\varepsilon = {{\,\textrm{proj}\,}}^i_\#\Pi _\varepsilon = \sigma ^i*\theta _\varepsilon$, for $i=1,2$, be the two marginals of the convolved plan.

We now define the deconvolved plan as introduced in [1, Theorem 6.3]:

$$\begin{aligned} \begin{aligned} {\tilde{\Pi }}_\varepsilon (x, y)&= \int _{\mathbb {R}^2} \frac{\sigma ^1(x)\theta _\varepsilon (x'-x)}{\sigma ^1_\varepsilon (x')} \frac{\sigma ^2(y)\theta _\varepsilon (y'-y)}{\sigma ^2_\varepsilon (y')} \Pi _\varepsilon (x',y') \textrm{d}x' \textrm{d}y' \\&= \int _{\mathbb {R}^2} P(x, y, x', y') \textrm{d}x' \textrm{d}y' . \end{aligned} \end{aligned}$$

We verify that ${\tilde{\Pi }}_\varepsilon \textrm{d}x \textrm{d}y\in \Pi (\sigma ^1,\sigma ^2)$. Indeed, for any integrable function $F: \mathbb {R}^2 \rightarrow \mathbb {R}$ and for every $x\in \mathbb {R}$ we perform this useful computation, integrating first the variable *y* and then $y'$5.1By taking the function $F\equiv 1$ this reads

$$\begin{aligned} \begin{aligned} \int _{\mathbb {R}} {\tilde{\Pi }}_\varepsilon (x,y) \textrm{d}y&= {\sigma ^1(x)}. \end{aligned} \end{aligned}$$

Analogously, one can show that the second marginal of ${\tilde{\Pi }}_\varepsilon$ is $\sigma ^2$. Moreover, ${\tilde{\Pi }}_\varepsilon$ is supported in a neighborhood of size $\varepsilon ^{1/4}$ of the support of $\Pi _\varepsilon$ because, in the definition of ${\tilde{\Pi }}_\varepsilon$, $(x',y')$ is in the support of $\Pi _\varepsilon$ and $x' -x$ is less than $\varepsilon ^{1/4}$. Therefore,

5.2 $$\begin{aligned} {\tilde{\Pi }}_\varepsilon \,\text{ is } \text{ supported } \text{ in } \text{ a } \text{ neighborhood } \text{ of } \text{ size }\, 2\varepsilon ^{1/4}\, \text{ of } \text{ the } \text{ support } \text{ of }\, \Pi _0. \end{aligned}$$

We claim in the next proposition that the operation of deconvolving a transport plan $\Pi _0$ worsens its potential energy in a controlled way, namely proportionally to the total mass of $\Pi _0$, while on the other hand the kinetic energy of the deconvolution is controlled only in terms of the kinetic energy of the marginals of $\Pi _0$. The loss in potential energy proportional to the mass of $\Pi _0$ is the reason why in the proof of Theorem 1.3 we use this deconvolution procedure only on the remaining mass, rather than on the full plan ${\bar{\gamma }}_\varepsilon$. Let us also point out that, in the proof of Theorem 1.3, $\Pi _0$ will in turn be an $\varepsilon$-dependent plan, built in Sect.  6 below. For this reason we keep explicit all dependences on $\Pi _0$ in the next proposition.

### Proposition 5.1

Let *V* be as in Theorem 1.3, $H>0$ and $\Omega _H, \Omega _H'$ be the sets introduced in (4.5), (4.6). Let $\sigma ^1,\sigma ^2\in \mathscr {M}_+(\mathbb {R})$ with the same mass, let $\Pi _0\in \Pi (\sigma ^1,\sigma ^2)$, and let ${\tilde{\Pi }}_\varepsilon$ be the plan introduced at the beginning of this section. Then there exists a universal constant $C>0$.

5.3 $$\begin{aligned} {{\,\textrm{KE}\,}}({\tilde{\Pi }}_\varepsilon ) \le {{\,\textrm{KE}\,}}(\sigma ^1) + {{\,\textrm{KE}\,}}(\sigma ^2) + \frac{C}{\sqrt{\varepsilon }}\Vert {\Pi _0}\Vert _1 \qquad \text {for every}\, \varepsilon >0. \end{aligned}$$

Moreover, there exists a constant $c_H>0$ depending only on *H* such that if

5.4 $$\begin{aligned} \textsf{d}\bigl ((x,y),{{\,\textrm{graph}\,}}(T)\bigr ) \le c_H{\varvec{1}}_{\Omega _H'}(x) \qquad \text {for every } (x,y)\in {{\,\textrm{supp}\,}}\Pi _0, \end{aligned}$$

then there exists a universal constant $C>0$ and a constant $C_H>0$ depending only on *H* such that for $\varepsilon$ sufficiently small

5.5 $$\begin{aligned} \frac{1}{ \sqrt{\varepsilon }} \int _{\mathbb {R}^2} V\textrm{d}{\tilde{\Pi }}_\varepsilon \le \frac{C_H}{ \sqrt{\varepsilon }} \int _{\mathbb {R}^2} V\textrm{d}\Pi _0 + C\Vert {\Pi _0}\Vert _1. \end{aligned}$$

We notice that the dependence of the constants in (5.5) is of fundamental importance: in particular the first term will go to 0 (at *H* fixed) by the particular choice of $\Pi _0$, while the second term will be estimated with (4.19) by the mass of $\rho$ outside $\Omega _H$ (times the constant *C* independent of *H*); hence this will be small sending $H \rightarrow \infty$ uniformly in $\varepsilon$.

### Remark 5.2

Another estimate of the potential energy of the deconvolved plan in terms of the original one was proposed by Lewin [25].

In our notations, tracing in his proof the dependence on $\Vert \Pi _0\Vert _1$, we would get an estimate of the form

$$\begin{aligned} \left| \int _{\mathbb {R}^2} \Phi \, d \bar{\Pi }_{\varepsilon } - \int _{\mathbb {R}^2} \Phi \, d \Pi _0 \right| \le \sqrt{\varepsilon } \left( \Vert \nabla \Phi \Vert _{\infty } \int |\nabla \sigma _1| + \Vert D^2 \Phi \Vert _{\infty } { \Vert \Pi _0\Vert _1} \right) , \end{aligned}$$

where the norms of $\nabla \Phi$ and $D^2\Phi$ are calculated in the neighbourhood of the graph of *T* where $\bar{\Pi }_{\varepsilon }$ is supported. Since $\bar{\Pi }_{\varepsilon }$ and $\Pi _0$ have the same marginals, we notice that

$$\begin{aligned} \int _{\mathbb {R}^2} V \, d \bar{\Pi }_{\varepsilon } - \int _{\mathbb {R}^2} V \, d \Pi _0 = \int _{\mathbb {R}^2} V_{ee} \, d \bar{\Pi }_{\varepsilon } - \int _{\mathbb {R}^2} V_{ee} \, d \Pi _0, \end{aligned}$$

Applying the estimate above for $\Phi =V_{ee}$ and using that $|x-y| \ge 1/ (2\Vert \rho \Vert _{\infty })$ on the graph of *T*, we would get that for $\varepsilon$ small enough

$$\begin{aligned} \left| \int _{\mathbb {R}^2} V\, d \bar{\Pi }_{\varepsilon } - \int _{\mathbb {R}^2} V \, d \Pi _0 \right| \le C\sqrt{\varepsilon } \Vert \rho \Vert _{\infty }^3 \sqrt{ \Vert \Pi _0\Vert _1} \cdot \left( \int |\nabla \sigma _1|+ \sqrt{ \Vert \Pi _0\Vert _1} \right) . \end{aligned}$$

However, this estimate is not good enough for our application since the *BV* norm of $\sigma _1$ does not go to 0 as $\varepsilon \rightarrow 0$ (recall that $\sigma _1$ coincides with $\rho$ in a neighborhood of 0).

### Proof

We have that

$$\begin{aligned} \begin{aligned} {{\,\textrm{KE}\,}}({\tilde{\Pi }}_\varepsilon )&= \frac{1}{2} \int _{\mathbb {R}^2} \left|{\nabla \sqrt{{\tilde{\Pi }}_\varepsilon ({{\varvec{x}}})}}\right|^2 \textrm{d}{{\varvec{x}}}= \int _{\mathbb {R}^2} \frac{|{\partial _x{\tilde{\Pi }}_\varepsilon }|^2 + |{\partial _y{\tilde{\Pi }}_\varepsilon }|^2}{8{\tilde{\Pi }}_\varepsilon } \textrm{d}x. \end{aligned} \end{aligned}$$

Let us deal with the first term; the second one is treated analogously. By Hölder inequality and since $\int _{\mathbb {R}^2} P \textrm{d}x' \textrm{d}y'= {\tilde{\Pi }}_\varepsilon (x,y)$, we have

$$\begin{aligned} \begin{aligned} |{\partial _x{\tilde{\Pi }}_\varepsilon (x,y)}|^2&= \left|{\int _{\mathbb {R}^2} \frac{\partial }{\partial x}\left( \frac{\sigma ^1(x)\theta _\varepsilon (x'-x)}{\sigma ^1_\varepsilon (x')}\right) \frac{\sigma ^2(y)\theta _\varepsilon (y'-y)}{\sigma ^2_\varepsilon (y')} \Pi _\varepsilon (x',y') \textrm{d}x' \textrm{d}y' }\right|^2 \\&= \left|{\int _{\mathbb {R}^2} \left( \frac{\partial _x\sigma ^1(x)}{\sigma ^1(x)} + \frac{\partial _x\theta _\varepsilon (x'-x)}{\theta _\varepsilon (x'-x)}\right) P(x,y,x',y') \textrm{d}x' \textrm{d}y' }\right|^2 \\&\le {\tilde{\Pi }}_\varepsilon (x,y) \int _{\mathbb {R}^2} \left[ \left( \frac{\partial _x\sigma ^1(x)}{\sigma ^1(x)}\right) ^2 + \left( \frac{\partial _x\theta _\varepsilon (x'-x)}{\theta _\varepsilon (x'-x)}\right) ^2 \right] \\&\quad \, P(x,y,x',y') \textrm{d}x' \textrm{d}y' . \end{aligned} \end{aligned}$$

Then

$$\begin{aligned} \begin{aligned}&\int _{\mathbb {R}^2} \frac{|{\partial _x{\tilde{\Pi }}_\varepsilon (x,y)}|^2}{8{\tilde{\Pi }}_\varepsilon (x,y)} \textrm{d}x \textrm{d}y \\&\quad = \int _{\mathbb {R}^4} \frac{1}{8} \left[ \left( \frac{\partial _x\sigma ^1(x)}{\sigma ^1(x)}\right) ^2 + \left( \frac{\partial _x\theta _\varepsilon (x'-x)}{\theta _\varepsilon (x'-x)}\right) ^2 \right] P(x,y,x',y') \textrm{d}x \textrm{d}y\textrm{d}x' \textrm{d}y' . \end{aligned} \end{aligned}$$

Applying (5.1) with $F(x,x')$ given by the expression in square brackets above and we find that

$$\begin{aligned} \begin{aligned} \int _{\mathbb {R}^2} \frac{|{\partial _x{\tilde{\Pi }}_\varepsilon (x,y)}|^2}{8{\tilde{\Pi }}_\varepsilon (x,y)} \textrm{d}x \textrm{d}y&= \int _{\mathbb {R}^4} \frac{1}{8} \left[ \left( \frac{\partial _x\sigma ^1(x)}{\sigma ^1(x)}\right) ^2 + \left( \frac{\partial _x\theta _\varepsilon (x'-x)}{\theta _\varepsilon (x'-x)}\right) ^2 \right] \\&\quad \times {\sigma ^1(x)\theta _\varepsilon (x'-x)}\textrm{d}x' \textrm{d}x \\&= \int _\mathbb {R}\frac{|{\partial _x\sigma ^1(x)}|^2}{8\sigma ^1(x)} \textrm{d}x + \Vert {\sigma ^1}\Vert _1 \int _\mathbb {R}\frac{|{\partial _x\theta _\varepsilon (z)}|^2}{8\theta _\varepsilon (z)} \textrm{d}z \\&= {{\,\textrm{KE}\,}}(\sigma _1) + {{\,\textrm{KE}\,}}(\theta _\varepsilon ) \Vert {\Pi _0}\Vert _1 . \end{aligned} \end{aligned}$$

The kinetic energy of the marginal of the rescaled convolution kernel scales as

$$\begin{aligned} \begin{aligned} {{\,\textrm{KE}\,}}(\theta _\varepsilon )&= \frac{1}{2} \int _\mathbb {R}\left|{\partial _x\sqrt{\theta _\varepsilon (x)}}\right|^2 \textrm{d}x = \int _\mathbb {R}\frac{|{\theta _\varepsilon '(x)}|^2}{8\theta _\varepsilon (x)} \textrm{d}x\\&= \int _\mathbb {R}\varepsilon ^{-3/4} \frac{|{\theta '(y)}|^2}{8\theta (y)} \varepsilon ^{1/4}\textrm{d}y = \varepsilon ^{-1/2} {{\,\textrm{KE}\,}}(\theta ), \end{aligned} \end{aligned}$$

and the kinetic energy of $\theta$ is a universal constant since $\theta$ is fixed. In conclusion, putting everything together, we obtain (5.3).

Let $c_{0, H}$ be any constant strictly less than the distance of ${{\,\textrm{graph}\,}}T$ from the diagonal $\{ x=y\}$. For $\varepsilon$ sufficiently small, ${{\,\textrm{supp}\,}}\Pi _\varepsilon$ will be supported at positive distance from the diagonal thanks to (5.2), as soon as the constant $c_0$ appearing there is less or equal than $c_{0,H}$. By using the Taylor expansion of *V* centered at $(x',y')$ and the fact that thanks to Lemma 4.1$\nabla ^2V$ is bounded by a universal constant *C* independent of *H* in the region of interest because we are far from the diagonal, we have that

$$\begin{aligned} \begin{aligned}&\int _{\mathbb {R}^2} V\textrm{d}\Pi _\varepsilon \\&\quad \le \int _{\mathbb {R}^4} V(x',y') \Theta _\varepsilon (x-x',y-y')\textrm{d}\Pi _0(x',y')\textrm{d}x\textrm{d}y\\&\qquad + \int _{\mathbb {R}^4} \nabla V(x',y')\cdot (x-x',y-y') \Theta _\varepsilon (x-x',y-y')\textrm{d}\Pi _0(x',y')\textrm{d}x\textrm{d}y\\&\qquad + \int _{\mathbb {R}^4} C\bigl (|{x-x'}|^2+|{y-y'}|^2\bigr ) \Theta _\varepsilon (x-x',y-y')\textrm{d}\Pi _0(x',y')\textrm{d}x\textrm{d}y \\&\quad \le \int _{\mathbb {R}^2} V\textrm{d}\Pi _0 + C\int _{\mathbb {R}^4} \bigl (|{x-x'}|^2+|{y-y'}|^2\bigr ) \Theta _\varepsilon (x-x',y-y')\textrm{d}\Pi _0(x',y')\textrm{d}x\textrm{d}y, \end{aligned} \end{aligned}$$

because the integral in the second line vanishes (it’s the integral of a linear function in *x*, *y* with respect to a symmetric kernel). The terms involving $|{x-x'}|^2$ and $|{y-y'}|^2$ in the last integral can be both computed as

$$\begin{aligned} \begin{aligned}&\int _{\mathbb {R}^4} |{y-y'}|^2 \Theta _\varepsilon (x-x',y-y') \textrm{d}\Pi _0(x',y') \textrm{d}x \textrm{d}y\\&\quad = \int _{\mathbb {R}^3} |{y-y'}|^2 \theta _\varepsilon (y-y') \textrm{d}\Pi _0(x',y') \textrm{d}y \\&\quad = \int _{\mathbb {R}^2} |{y-y'}|^2 \theta _\varepsilon (y-y') \textrm{d}y \textrm{d}\sigma ^2(y') \\&\quad = C\sqrt{\varepsilon }\Vert {\sigma ^2}\Vert _1 = C\sqrt{\varepsilon }\Vert {\Pi _0}\Vert _1 . \end{aligned} \end{aligned}$$

Therefore

$$\begin{aligned} \int V \textrm{d}\Pi _\varepsilon \le \int V \textrm{d}\Pi _0 + C\sqrt{\varepsilon }\Vert {\Pi _0}\Vert _1 . \end{aligned}$$

Using again the Taylor expansion of *V* centered at $(x',y')$ we can proceed to estimate that

5.6 $$\begin{aligned} \begin{aligned} \int V(x,y)\textrm{d}{\tilde{\pi }}_\varepsilon (x,y)&\le \int _{\mathbb {R}^4} V(x',y') P(x,y,x',y')\textrm{d}x\textrm{d}y\textrm{d}x'\textrm{d}y'\\&\quad + \int _{\mathbb {R}^4} \nabla V(x',y')\cdot (x-x',y-y') P(x,y,x',y')\textrm{d}x\textrm{d}y\textrm{d}x'\textrm{d}y'\\&\quad + \int _{\mathbb {R}^4} C\bigl (|{x-x'}|^2+|{y-y'}|^2\bigr ) P(x,y,x',y')\textrm{d}x\textrm{d}y\textrm{d}x'\textrm{d}y' . \end{aligned} \end{aligned}$$

By the definition of *P* and integrating first the variable *x* and secondly the variable *y*, the first integral in the right-hand side isGiven $x\in \Omega _H'$ and *y* such that $d((x,y), {{\,\textrm{graph}\,}}T) \le c_{0,H}$, let $(x_0,y_0)\in {{\,\textrm{graph}\,}}(T)$ be a projection, i.e. a point realising the minimum of the distance, and let $v=(x-x_0,y-y_0)$ be the difference vector and $d=\textsf{d}\bigl ((x,y),{{\,\textrm{graph}\,}}(T)\bigr )=|{v}|$ the distance. By looking at the Taylor expansion of *V* at $(x_0, y_0)$, recalling that $\nabla V(x_0, y_0)=0$, we have that

$$\begin{aligned} |{V(x,y)-v^T \nabla ^2V(x_0,y_0) v}| \le \Vert {\nabla ^3 V}\Vert _\infty d^3 \le C_H d^3, \end{aligned}$$

where the $L^\infty$ norm is taken over the set of points (*x*, *y*) such that $x\in \Omega _H'$ and *y* such that $d((x,y), {{\,\textrm{graph}\,}}T) \le c_{0,H}$. This $L^\infty$ norm is in turn estimated by $C_H$ thanks to the explicit computation of the derivatives of *V* (see Lemma 4.1) and thanks to the quantities that we have under control at *H* fixed in (4.7). The difference vector *v* is orthogonal to ${{\,\textrm{graph}\,}}(T)$ at $(x_0,y_0)$, therefore it is in the direction of the eigenvector of $\sqrt{\nabla ^2 V(x_0,y_0)}$ associated to the non-zero eigenvalue $q(x_0,y_0)$. Therefore

$$\begin{aligned} V(x,y) \ge v^T \nabla ^2 V(x_0,y_0) v - \Vert {\nabla ^3 V}\Vert _\infty d^3 \ge q^2 d^2 - C_H d^3 \ge \frac{1}{2} \Bigl (\min _{\Omega _H'} q\Bigr )^2 d^2 \end{aligned}$$

as soon as $d\le \frac{1}{2} C_H^{-1} \bigl (\min _{\Omega _H'} q\bigr )^2$. Therefore, for

5.7 $$\begin{aligned} \textsf{d}\bigl ((x,y),{{\,\textrm{graph}\,}}(T)\bigr ) < \min \big \{ c_{0,H}, \frac{1}{2} C_H^{-1} \bigl (\min _{\Omega _H'} q\bigr )^2 \big \}=:c_L, \end{aligned}$$

we have

5.8 $$\begin{aligned} |{\nabla V(x,y)}|^2 \le \Vert {\nabla ^2 V}\Vert _{\infty }^2 d^2 \le 2 \Vert {\nabla ^2 V}\Vert _{\infty }^2 \Vert {1/q}\Vert _{L^\infty (\Omega _H')}^2 V(x,y) \le C_H V(x,y) . \end{aligned}$$

Using Cauchy-Schwarz, the second integral in (5.6) can be bounded by

$$\begin{aligned} \begin{aligned} \int _{\mathbb {R}^4}&\nabla V(x',y')\cdot (x-x',y-y') P(x,y,x',y')\textrm{d}x\textrm{d}y\textrm{d}x'\textrm{d}y' \\&\le \int _{\mathbb {R}^4} \bigl (|{\nabla V(x',y')}|^2 + (|{x-x'}|^2+|{y-y'}|^2)\bigr ) P(x,y,x',y')\textrm{d}x\textrm{d}y\textrm{d}x'\textrm{d}y' . \end{aligned} \end{aligned}$$

Using (5.8) in the point $(x' y')$, which for $\varepsilon$ sufficiently small satisfies (5.7) thanks to (5.4) and (5.2), the first term can be estimated as

$$\begin{aligned} \int _{\mathbb {R}^4}&|{\nabla V(x',y')}|^2 P(x,y,x',y') \textrm{d}x \textrm{d}y \textrm{d}x' \textrm{d}y' = \int _{\mathbb {R}^2} |{\nabla V(x',y')}|^2 \Pi _\varepsilon (x',y') \textrm{d}x' \textrm{d}y' \\&= \int _{\mathbb {R}^4} |{\nabla V(x,y)}|^2 \Theta _\varepsilon (x-x',y-y') \textrm{d}\Pi _0(x',y') \textrm{d}x \textrm{d}y \\&\le 2\int _{\mathbb {R}^4} |{\nabla V(x',y')}|^2 \Theta _\varepsilon (x-x',y-y') \textrm{d}\Pi _0(x',y') \textrm{d}x \textrm{d}y \\&\quad + 2\int _{\mathbb {R}^4} |{\nabla V(x,y)-\nabla V(x',y')}|^2 \Theta _\varepsilon (x-x',y-y') \textrm{d}\Pi _0(x',y') \textrm{d}x \textrm{d}y \\&\le 2\int _{\mathbb {R}^2} |{\nabla V(x',y')}|^2 \textrm{d}\Pi _0(x',y') + 2\bigl ({{\,\textrm{Lip}\,}}(\nabla V)\bigr )^2\\&\quad \times \int _{\mathbb {R}^4} \bigl (|{x-x'}|^2+|{y-y'}|^2\bigr ) \Theta _\varepsilon (x-x',y-y')\textrm{d}\Pi _0(x',y')\textrm{d}x\textrm{d}y \\&\le C_H \int _{\mathbb {R}^2} V\textrm{d}\Pi _0 + C\sqrt{\varepsilon }\Vert {\Pi _0}\Vert _1 . \end{aligned}$$

The third integral in (5.6) can be computed exploiting (5.1) with $F(x,x') = |x-x'|^2$ to get

$$\begin{aligned} \begin{aligned} \int _{\mathbb {R}^4} |{x-x'}|^2P \textrm{d}x \textrm{d}y \textrm{d}x' \textrm{d}y'&= \int _{\mathbb {R}^2} |{x-x'}|^2 \theta _\varepsilon (x'-x)\sigma ^1(x)\textrm{d}x\textrm{d}x'\\&=\Vert {\sigma ^1}\Vert _1 \int _{\mathbb {R}} |z|^2 \theta _\varepsilon (z) \textrm{d}z = C\sqrt{\varepsilon }\Vert {\sigma ^1}\Vert _1 = C\sqrt{\varepsilon }\Vert {\Pi _0}\Vert _1 \end{aligned} \end{aligned}$$

and analogously for the term with $|y-y'|^2$. Putting the pieces together, we obtain

$$\begin{aligned} \begin{aligned} \int _{\mathbb {R}^2} V\textrm{d}{\tilde{\Pi }}_\varepsilon&\le C_H \int _{\mathbb {R}^2} V\textrm{d}\Pi _\varepsilon + C\sqrt{\varepsilon }\Vert {\Pi _0}\Vert _1 . \end{aligned} \end{aligned}$$

$$\square $$

## Potential cost of the remaining mass and kinetic energy of its marginals

In Sect.  4 we described how to build the main portion of the recovery sequence ${\bar{\gamma }}_\varepsilon$. As stated in (4.18)–(4.19), this procedure leaves out a small positive mass that we still have to account for. The goal of this section is to build a transport plan for the remaining mass which satisfies suitable energy bounds and allows to apply the deconvolution procedure of Sect.  5, namely Proposition 5.1. To this end we need to build a transport plan $\pi \in \Pi (\rho -\rho ^1_\varepsilon , \rho -\rho ^2_\varepsilon )$ with potential energy smaller than $\sqrt{\varepsilon }$ and to have a suitable control on how the kinetic energy of its marginals explodes in $\varepsilon$. This is the content of the following proposition and this section is dedicated to its proof.

### Proposition 6.1

Let $\rho \in C^1(\mathbb {R})$, $\gamma \in \Pi (\rho ,\rho )$, $u\in W^{2,\infty }$ and $V(x,y)=|{x-y}|^{-1}-u(x)-u(y)$ be as in Theorem 1.3. Let ${\bar{\gamma }}_\varepsilon$ be the plan introduced in (4.14) and (4.15) and let $\rho ^1_\varepsilon = {{\,\textrm{proj}\,}}^1_\# {\bar{\gamma }}_\varepsilon$ and $\rho ^2_\varepsilon = {{\,\textrm{proj}\,}}^2_\# {\bar{\gamma }}_\varepsilon$ be its two marginals introduced in (4.16). Then for every $c>0$ and for some constant $C_H>1$ depending only on *H*

i) (construction of a plan with $o_L(\sqrt{\varepsilon })$ potential energy) for every $\varepsilon$ sufficiently small there is $\pi _\varepsilon \in \Pi (\rho -\rho ^1_\varepsilon , \rho -\rho ^2_\varepsilon )$ such that
  6.1 $$\begin{aligned} \int V\textrm{d}\pi _\varepsilon \le C_H\left( \frac{\delta ^4}{\tau } + \frac{\varepsilon N^2}{\beta \tau } + \frac{\beta \delta ^2}{\tau } \right) \end{aligned}$$
  and
  6.2 $$\begin{aligned} \textsf{d}\bigl ((x,y),{{\,\textrm{graph}\,}}(T)\bigr ) \le c {\varvec{1}}_{\Omega _H'}(x) \qquad \text {for every } (x,y)\in {{\,\textrm{supp}\,}}\Pi _0; \end{aligned}$$
ii) (bound on the kinetic energy of the marginals) we have
  6.3 $$\begin{aligned} {{\,\textrm{KE}\,}}(\rho -\rho ^1_\varepsilon ) + {{\,\textrm{KE}\,}}(\rho -\rho ^2_\varepsilon ) \le \frac{C_H}{\tau }\left( {{\,\textrm{KE}\,}}(\rho ) + \frac{1}{\beta }\right) , \end{aligned}$$

Notice that the right hand side of (6.1) is $o_H(\varepsilon ^{1/2})$ and the right hand side of (6.3) is $o_H(\varepsilon ^{-1/2})$ as $\varepsilon \rightarrow 0$ because of (4.9)-(4.11).

To build the plan $\pi _\varepsilon$, we will first estimate the Wasserstein distance between two suitable measures. We reduce to this estimate since an essential tool of its proof is the Benamou-Brenier formulation of optimal transport, which is not available with a general potential *V*. As a matter of fact fact, even a slightly more precise estimate than the bound on the Wasserstein distance by $o_L(\varepsilon ^{1/2})$ is needed: namely, we need a map with $o_L(\varepsilon ^{1/2})$ cost and with a control on the support. Since this second condition is more technical and comes naturally from the proof of (6.4), the reader may decide to skip it at a first reading and focus on (6.4).

### Proposition 6.2

Let $\Omega _H''$ be the convex hull of $\Omega _H'$. Under the same assumptions as in Proposition 6.1, there exists a constant $C_H$ depending only on *H* such that

6.4 $$\begin{aligned} W_2^2\left( \rho -\rho ^2_\varepsilon , {T_\delta }_\#(\rho -\rho ^1_\varepsilon )\right) \le C_H\left( \frac{\delta ^4}{\tau } + \frac{\varepsilon N^2}{\beta \tau } + \frac{\beta \delta ^2}{\tau } \right) \end{aligned}$$

Moreover, the optimal map $S_\varepsilon$ between $\rho -\rho ^2_\varepsilon$ and ${T_\delta }_\#(\rho -\rho ^1_\varepsilon )$ satisfies a pointwise bound

6.5 $$\begin{aligned} |{S_\varepsilon (x)-x}| \le C_H\left( \frac{\delta ^4}{\tau ^2} + \frac{\varepsilon N^2}{\beta \tau ^2} + \frac{\beta \delta ^2}{\tau ^2} \right) ^{1/3} {\varvec{1}}_{\Omega _H'}(x) \qquad \text {for a.e.}\, x\in \mathbb {R}. \end{aligned}$$

In particular, the right-hand sides of (6.4) and (6.5) are $o_H(\sqrt{\varepsilon })$ and $O_H(1)$ respectively as soon as (4.9) and (4.11) are satisfied.

We immediately show how Proposition 6.1(i) can be deduced from this estimate.

### Proof of Proposition 6.1(i) from Proposition 6.2

Let $\sigma _1 = \rho -\rho ^1_\varepsilon$, $\sigma _2 = \rho -\rho ^2_\varepsilon$ and let $S_\varepsilon$ be the map given by Proposition 6.2 . Let then $\pi _\varepsilon =(\textrm{Id}, S_\varepsilon \circ T_\delta )_\#\sigma _1\in \Pi (\sigma _1,\sigma _2)$. We observe that (recalling that $T_\delta$ maps $\Omega _H'$ in itself and coincides with *T* outside, and that $S_\varepsilon (x)= x$ outside $\Omega _H'$)

6.6 $$\begin{aligned} \begin{aligned} |{S_\varepsilon (T_\delta (x)) - T(x)}|&\le |{S_\varepsilon (T_\delta (x)) - T_\delta (x)}| + |{T_\delta (x) - T(x)}| \\&\le \left[ C_H\left( \frac{\delta ^4}{\tau ^2} + \frac{\varepsilon N^2}{\beta \tau ^2} + \frac{\beta \delta ^2}{\tau ^2} \right) + L \delta ^2\right] {\varvec{1}}_{\Omega _H'}(x) \end{aligned} \end{aligned}$$

and the right-hand side converges to 0 as $\varepsilon \rightarrow 0$, therefore proving (6.2) for $\varepsilon$ sufficiently small. Under the assumptions of Proposition 6.1 we have that (4.4) of Lemma 4.1 holds, that is $V(x,y) \le C_H \textsf{d}\bigl ((x,y),{{\,\textrm{graph}\,}}(T)\bigr )^2$ in a neighborhood (independent of $\varepsilon$) of ${{\,\textrm{graph}\,}}(T)$. For $\varepsilon$ sufficiently small, the plan $\pi _\varepsilon$ is supported in a neighborhood of ${{\,\textrm{graph}\,}}(T)$ which converges to 0 as $\varepsilon \rightarrow 0$ thanks to (6.5), so in particular the quadratic upper bound for *V* applies. Therefore,

$$\begin{aligned}\begin{aligned} \int V(x,y) \textrm{d}\pi _\varepsilon (x,y)&= \int _{\Omega _H'} V(x,S_\varepsilon (T_\delta (x))) \textrm{d}\sigma _1(x) \\&\le C_H\int _{\Omega _H'} |{S_\varepsilon (T_\delta (x))-T_\delta (x)-T(x)+T_\delta (x)}|^2 \textrm{d}\sigma _1(x) \\&\le 2C_H \int _{\Omega _H'} |{S(T_\delta (x))-T_\delta (x)}|^2 \textrm{d}\sigma _1(x) + 2C_H \Vert {T-T_\delta }\Vert _\infty ^2 \\&\le 2C_H \int _{\Omega _H'} |{S(x)-x}|^2 \textrm{d}({T_\delta }_\#\sigma _1)(x) + 2C_H(L\delta ^2)^2 \\&\le C_H\left( \frac{\delta ^4}{\tau } + \frac{\varepsilon N^2}{\beta \tau } + \frac{\beta \delta ^2}{\tau } \right) . \end{aligned} \end{aligned}$$

$$\square $$

The estimate (6.4) relies on the particular structure that the marginals of the convolved plan have in the model case when *T* is (locally) a linear map, which is the main reason why we introduce $T_\delta$ in the construction. We carry out this surprising computation in Sect.  6.1 below. This computation could be interpreted by seeing the convolutions with Gaussian measures as the action of the heat semigroup. However, in our context the Gaussians need to be truncated to have a finite-range interaction, since the control of the potential is only local around its 0 level set rather than global, and hence this analogy remains only formal and we proceed in a different way.

It can be seen that $\rho$ and $T_\delta \rho$ are very close in Wasserstein distance, as well as $\rho ^2_\varepsilon$ and ${T_\delta }_\#\rho ^1_\varepsilon$. This sole property does not guarantee in general that they remain close when we subtract each other, namely when we consider the measures $\rho -\rho ^2_\varepsilon$ and ${T_\delta }_\#(\rho -\rho ^1_\varepsilon )$: for instance, when one considers the three measures $\nu _1 = 1_{[0,1]}{\mathcal {L}}$, $\nu _2 = 1_{[\alpha ,1+\alpha ]}{\mathcal {L}}$ and $\nu _0 = 1_{[\alpha ,1]}{\mathcal {L}}$, then $W_2(\nu _1, \nu _2) = \alpha$ but $W_2(\nu _1- \nu _0, \nu _2-\nu _0) =\sqrt{\alpha }\gg \alpha$ as $\alpha \rightarrow 0$. However, in our particular situation we can control the distance (6.4) via the Benamou-Brenier formula, since we can provide an interpolating curve between $\rho -\rho ^2_\varepsilon$ and ${T_\delta }_\#(\rho -\rho ^1_\varepsilon )$ which enjoys some extra properties. For instance, we estimate how close the density of the interpolating curve is to that of the endpoints, providing then a lower bound on this density along the entire curve.

The proof of Proposition 6.2 in Sect.  6.2 deals then with this Benamou-Brenier estimate and combines it with a linearization argument, which allows to pass from *T* to its piecewise linear approximation $T_\delta$, and with a suitable control of the errors generated at the interface between two consecutive intervals where $T_\delta$ is linear. A crucial ingredient of the proof is the particular way the two marginals of a truncated Gaussian can be mapped one onto the other at a low cost, which is explained in Sect.  6.1.

Finally, we dedicate Sect.  6.3 to the kinetic energy bounds of Proposition 6.1(ii).

### Marginals of kernels of product type along a linear map

In this section we study the properties of the marginals of the rectangularly truncated Gaussian (3.1), which is a convolution kernel of product type

$$\begin{aligned} \Gamma _{M_{\varepsilon ,\beta },N}({{\varvec{x}}}) = \frac{\left( e^{-\left( \frac{q}{\varepsilon ^{1/2}}+\frac{1}{\beta }\right) w^2/2} - e^{-N/2}\right) _+^2}{G_{\frac{q}{\varepsilon ^{1/2}}+\frac{1}{\beta },N}} \cdot \frac{\left( e^{-\frac{1}{\beta }z^2/2} - e^{-N/2}\right) _+^2}{G_{\frac{1}{\beta },N}} = h^1_\varepsilon (w) h^2_\varepsilon (z) \end{aligned}$$

where $M_{\varepsilon ,\beta }=A/\varepsilon ^{1/2}+I/\beta$ as usual, *q* is the positive eigenvalue of *A* which is assumed to lie in a certain interval [1/*L*, *L*], *z* is the coordinate in the direction of $\ker (A)$ and *w* is the transversal coordinate. The change of variables is given by the rotation

$$\begin{aligned} (w,z) = R(x,y) = \left( \frac{y-ax}{\sqrt{1+a^2}}, \frac{ay+x}{\sqrt{1+a^2}} \right) = (\cos \theta \,y-\sin \theta \,x, \sin \theta \,y+\cos \theta \,x) . \end{aligned}$$

where $(1,a)\in \ker (A)$, hence $\ker (A)={{\,\textrm{graph}\,}}(a\textrm{Id}+b)$, and $\theta =\arctan (a)$. Observe that $c_L< \theta < \pi /2 - c_L$ for some constant $c_L>0$ depending only on *L*.

In fact, for the computations of this section the precise form of $h^i_\varepsilon$ does not play a role: we only need $h^1_\varepsilon$ and $h^2_\varepsilon$ to be positive, symmetric $L^1$ functions supported in an interval of length $C_H \varepsilon ^{1/4}N^{1/2}$ and $\beta ^{1/2}N^{1/2}$ respectively, and with

6.7 $$\begin{aligned} \beta ^{1/2} \Vert \partial _z h^2_\varepsilon (z)\Vert _{L^1} + \beta \Vert \partial ^2_z h^2_\varepsilon (z)\Vert _{L^1} \le C . \end{aligned}$$

This property is satisfied by our kernel, since

$$\begin{aligned} (h^2_\varepsilon )'(z)= &   -2G_{\frac{1}{\beta },N}^{-1} \frac{z}{\beta }e^{-\frac{1}{\beta }z^2/2} \left( e^{-\frac{1}{\beta }z^2/2} - e^{-N/2}\right) _+,\\ (h^2_\varepsilon )''(z)= &   2G_{\frac{1}{\beta },N}^{-1} e^{-\frac{1}{\beta }z^2/2}\Big [\Big (-\frac{1}{\beta }+\frac{z^2}{\beta ^2} \Big ) \left( e^{-\frac{1}{\beta }z^2/2} - e^{-N/2}\right) _+ \\  &   + \frac{z^2}{\beta ^2} {\varvec{1}}_{\bigl [-\sqrt{\beta N},\sqrt{\beta N}\bigr ]}(z)\Big ] , \end{aligned}$$

from which

$$\begin{aligned} \beta ^{1/2} |{(h^2_\varepsilon )'(z)}| + \beta |{(h^2_\varepsilon )''(z)}| \le 2 G_{\frac{1}{\beta },N}^{-1} \left( \frac{|{z}|}{\beta ^{1/2}}+ 1 + 2\frac{z^2}{\beta } \right) e^{-\frac{1}{\beta }z^2} {\varvec{1}}_{\bigl [-\sqrt{\beta N},\sqrt{\beta N}\bigr ]}(z); \end{aligned}$$

integrating this inequality and disregarding the last factor ${\varvec{1}}_{\bigl [-\sqrt{\beta N},\sqrt{\beta N}\bigr ]}(z)$ we get (6.7).

We adopt the following notation to denote rescaled functions by putting a subscript between curly braces

$$\begin{aligned} \varphi _{\{\zeta \}}(x) = \frac{1}{\zeta }\varphi \left( \frac{x}{\zeta }\right) \end{aligned}$$

and we observe that $\Vert {\varphi _{\{\zeta \}}}\Vert _{L^1} = \Vert {\varphi }\Vert _{L^1}$, $(f*g)_{\{\zeta \}} = f_{\{\zeta \}}*g_{\{\zeta \}}$ and $(a\textrm{Id}+b)_\#\rho (y) = \rho _{\{a\}}(y-b)$.

Let $\eta _\varepsilon ^i = {{\,\textrm{proj}\,}}^i_\#\Gamma _{M_{\varepsilon ,\beta },N}$ be the two marginals of the Gaussian, namely,

$$\begin{aligned} \eta ^1_\varepsilon (x)&= \int _\mathbb {R}h^1_\varepsilon (w) h^2_\varepsilon (z) \textrm{d}y = \int _\mathbb {R}h^1_\varepsilon (\cos \theta \,y-\sin \theta \,x) h^2_\varepsilon (\sin \theta \,y+\cos \theta \,x) \textrm{d}y, \\ \eta ^2_\varepsilon (y)&= \int _\mathbb {R}h^1_\varepsilon (w) h^2_\varepsilon (z) \textrm{d}x = \int _\mathbb {R}h^1_\varepsilon (\cos \theta \,y-\sin \theta \,x) h^2_\varepsilon (\sin \theta \,y+\cos \theta \,x) \textrm{d}x, \end{aligned}$$

and ${\tilde{\eta }}_\varepsilon ^2=(a\textrm{Id})_\#\eta ^1_\varepsilon$. We claim that we can write them as a convolution of rescalings of $h^1_\varepsilon$ and $h^2_\varepsilon$, where the only difference between $\eta _\varepsilon ^2$ and ${\tilde{\eta }}_\varepsilon ^2$ lies in the parameter of rescaling of the first function

$$\begin{aligned} \eta ^2_\varepsilon (y)&= (h^1_\varepsilon )_{\{\sin \theta \cot \theta \}} * (h^2_\varepsilon )_{\{\sin \theta \}} , \\ {\tilde{\eta }}^2_\varepsilon (y)&= (h^1_\varepsilon )_{\{\sin \theta \tan \theta \}} * (h^2_\varepsilon )_{\{\sin \theta \}} . \end{aligned}$$

Indeed, with the change of variable $\cos \theta \,y-\sin \theta \,x = \frac{t-x}{\sin \theta }$, which rewrites also as $\sin \theta \,y+\cos \theta \,x = \frac{t}{\cos \theta }$ and $\textrm{d}y = \frac{\textrm{d}t}{\sin \theta \cos \theta }$, and using the fact that $h^1_\varepsilon$ is symmetric, we can compute

6.8 $$\begin{aligned} \begin{aligned} \eta ^1_\varepsilon (x)&= \int _\mathbb {R}\frac{1}{\sin \theta } h^1_\varepsilon \left( \frac{t-x}{\sin \theta }\right) \frac{1}{\cos \theta } h^2_\varepsilon \left( \frac{t}{\cos \theta }\right) \textrm{d}t \\&= \int _\mathbb {R}(h^1_\varepsilon )_{\{\sin \theta \}}(x-t) \cdot (h^2_\varepsilon )_{\{\cos \theta \}}(t) \textrm{d}t = \bigl [(h^1_\varepsilon )_{\{\sin \theta \}} * (h^2_\varepsilon )_{\{\cos \theta \}} \bigr ](x) . \end{aligned} \end{aligned}$$

In a similar fashion, with another substitution $\cos \theta \,y-\sin \theta \,x = \frac{t}{\cos \theta }$, which implies $\sin \theta \,y+\cos \theta \,x = \frac{y-t}{\sin \theta }$ and $\textrm{d}x = \frac{\textrm{d}t}{\sin \theta \cos \theta }$, we find that

6.9 $$\begin{aligned} \begin{aligned} \eta ^2_\varepsilon (y)&= \int _\mathbb {R}\Gamma _{M_{\varepsilon ,\beta },N}(x,y) \textrm{d}x = \int _\mathbb {R}h^1_\varepsilon (w) h^2_\varepsilon (z) \textrm{d}x \\&= \int _\mathbb {R}\frac{1}{\cos \theta } h^1_\varepsilon \left( \frac{t}{\cos \theta }\right) \frac{1}{\sin \theta } h^2_\varepsilon \left( \frac{y-t}{\sin \theta }\right) \textrm{d}t \\&= \int _\mathbb {R}(h^1_\varepsilon )_{\{\cos \theta \}}(t) \cdot (h^2_\varepsilon )_{\{\sin \theta \}}(y-t) \textrm{d}t = \bigl [(h^1_\varepsilon )_{\{\cos \theta \}} * (h^2_\varepsilon )_{\{\sin \theta \}} \bigr ](y) . \end{aligned} \end{aligned}$$

Finally, by the properties of the rescalings, we have that

$$\begin{aligned} \begin{aligned} {\tilde{\eta }}^2_\varepsilon = (\tan \theta \textrm{Id})_\#\eta ^1_\varepsilon = (\eta ^1_\varepsilon )_{\{\tan \theta \}} = (h^1_\varepsilon )_{\{\sin \theta \,\tan \theta \}} * (h^2_\varepsilon )_{\{\sin \theta \}}. \end{aligned} \end{aligned}$$

A simple consequence of these representations of $\eta ^i_\varepsilon$ is for instance an $L^\infty$ bound which will be useful later: from (6.8), since $\Vert (h^1_\varepsilon )_{\{\sin \theta \}}\Vert _{L^1}= \Vert h^1_\varepsilon \Vert _{L^1}= 1$ and by (7.2) we have

6.10 $$\begin{aligned} \Vert \eta ^1_\varepsilon \Vert _{L^\infty } \le \Vert (h^1_\varepsilon )_{\{\sin \theta \}}\Vert _{L^1} \Vert (h^2_\varepsilon )_{\{\cos \theta \}}\Vert _{L^\infty } \le C_H \Vert h^2_\varepsilon \Vert _{L^\infty } \le \frac{C_H}{G_{\frac{1}{\beta },N}} \le \frac{C_H}{G_{\frac{1}{\beta },\infty }} = \frac{C_H}{\beta ^{1/2}} . \end{aligned}$$

The next proposition considers a plan $\lambda$ supported on the graph of $(a\textrm{Id}+b)$ and introduces the convolved plan $\lambda *\Gamma _{M_{\varepsilon ,\beta },N}$, its marginals $\rho ^1_\varepsilon$ and $\rho ^2_\varepsilon$ and the pushforward ${\tilde{\rho }}^2_\varepsilon$ of the first marginal through the linear map $(a\textrm{Id}+b)$; it uses the representation of the marginals in terms of convolutions of $\eta _\varepsilon ^i$ with the marginal of $\lambda$ to provide estimates on their distance and an interpolating curve between $\rho ^2_\varepsilon$ and ${\tilde{\rho }}^2_\varepsilon$.

#### Proposition 6.3

Let $a\in (L^{-1},L)$, $\theta =\arctan (a)$, $b\in \mathbb {R}$, *A* a degenerate positive-semidefinite symmetric $2\times 2$ matrix with a positive eigenvalue $q\in (L^{-1},L)$, $\ker (A)={{\,\textrm{graph}\,}}(a\textrm{Id})$ and $M_{\varepsilon ,\beta }=A/\varepsilon ^{1/2}+I/\beta$.

Given $\lambda \in \mathscr {M}_+(\mathbb {R}^2)$ with ${{\,\textrm{supp}\,}}\lambda \subset {{\,\textrm{graph}\,}}(a\textrm{Id}+b)$, define $\lambda _\varepsilon = \lambda * \Gamma _{M_{\varepsilon ,\beta },N}$, let $\rho ^i = {{\,\textrm{proj}\,}}^i_\#\lambda$, $\rho ^i_\varepsilon = {{\,\textrm{proj}\,}}^i_\#\lambda _\varepsilon$ for $i=1,2$ and define ${\tilde{\rho }}^2_\varepsilon = (a\textrm{Id}+b)_\#\rho ^1_\varepsilon$. Then we have that

$$\begin{aligned} \mu _t = (h^1_\varepsilon )_{\{\sin \theta \,(\tan \theta )^{2t-1}\}} * (h^2_\varepsilon )_{\{\sin \theta \}} * \rho ^2, \qquad t\in [0,1], \end{aligned}$$

is a curve interpolating between $\rho ^2_\varepsilon$ and ${\tilde{\rho }}^2_\varepsilon$ satisfying $\partial _t \mu _t = {{\,\textrm{div}\,}}(m_t)$ with

6.11 $$\begin{aligned} \Vert {\mu _t - \rho ^2_\varepsilon }\Vert _\infty \le CL \frac{\varepsilon ^{1/2}N}{\beta } \Vert {\rho ^2}\Vert _\infty , \end{aligned}$$

$\mu _t$ and $m_t$ supported on the convex hull of ${{\,\textrm{supp}\,}}{\tilde{\rho }}^2_\varepsilon \cup {{\,\textrm{supp}\,}}\rho ^2_\varepsilon$ and

6.12 $$\begin{aligned} |{m_t}| \le C_H \frac{\varepsilon ^{1/2} N}{\beta ^{1/2}} \Vert {\rho ^2}\Vert _\infty . \end{aligned}$$

#### Proof

Up to a translation, we may assume that $b=0$. We have that $\rho ^i_\varepsilon = \eta ^i_\varepsilon *\rho ^i$ and, recalling that the kernel $h^2_\varepsilon$ is symmetric,

$$\begin{aligned} \begin{aligned} \mu _t(y) - \rho ^2_\varepsilon (y)&= \int _\mathbb {R}\bigl [(h^1_\varepsilon )_{\{\sin \theta \,(\tan \theta )^{2t-1}\}} - (h^1_\varepsilon )_{\{\sin \theta \cot \theta \}}\bigr ](y-z)\\&\quad \cdot \bigl ((h^2_\varepsilon )_{\{\sin \theta \}} * \rho ^2\bigr )(z) \textrm{d}z \\&= \int _\mathbb {R}\bigl [(h^1_\varepsilon )_{\{\sin \theta \,(\tan \theta )^{2t-1}\}} - (h^1_\varepsilon )_{\{\sin \theta \cot \theta \}}\bigr ](y-z) \\&\quad \cdot \biggl [\bigl ((h^2_\varepsilon )_{\{\sin \theta \}} * \rho ^2\bigr )(y) + \bigl ((h^2_\varepsilon )_{\{\sin \theta \}} * \rho ^2\bigr )'(y) \cdot (z-y) \\&\quad + \frac{1}{2}\bigl ((h^2_\varepsilon )_{\{\sin \theta \}} * \rho ^2\bigr )''(y_z) \cdot (z-y)^2 \biggr ] \textrm{d}z \\&= \int _\mathbb {R}\bigl [(h^1_\varepsilon )_{\{\sin \theta \,(\tan \theta )^{2t-1}\}} - (h^1_\varepsilon )_{\{\sin \theta \cot \theta \}}\bigr ](y-z) \\&\quad \cdot \frac{\bigl ((h^2_\varepsilon )_{\{\sin \theta \}} * \rho ^2\bigr )''(y_z)}{2} \cdot (z-y)^2 \textrm{d}z, \end{aligned} \end{aligned}$$

where $y_z$ is a point in the segment between *y* and *z*. Therefore, since the integrand is nonzero only for $|y-z|^2 \le C_H \varepsilon ^{1/2}N$ and thanks to (6.7), we obtain (6.11)

$$\begin{aligned} \begin{aligned} \Vert {\mu _t - \rho ^2_\varepsilon }\Vert _\infty&\le C_H\left\Vert {(h^1_\varepsilon )_{\{\sin \theta \,(\tan \theta )^{2t-1}\}} - (h^1_\varepsilon )_{\{\sin \theta \cot \theta \}}}\right\Vert _1\\&\quad \cdot \left\Vert {[(h^2_\varepsilon )_{\{\sin \theta \}} * \rho ^2]''}\right\Vert _\infty \varepsilon ^{1/2}N \\&\le C_H \varepsilon ^{1/2}N \Vert {[(h^2_\varepsilon )_{\{\sin \theta \}}]''}\Vert _1 \cdot \Vert {\rho ^2}\Vert _\infty \le C_H \frac{\varepsilon ^{1/2}N}{\beta } \Vert {\rho ^2}\Vert _\infty . \end{aligned} \end{aligned}$$

Define $H^1_\varepsilon (x) = \int _{-\infty }^x rh^1_\varepsilon (r)\textrm{d}r$, so that $(H^1_\varepsilon )_{\{\zeta \}}(x)$ is the primitive of $\frac{x}{\zeta ^2}(h^1_\varepsilon )_{\{\zeta \}}(x)$ with the same compact support.

This allows us to express the derivative of $(h^1_\varepsilon )_{\{\zeta \}}(x)$ with respect to the parameter $\zeta$ as

$$\begin{aligned} \begin{aligned} \frac{\partial (h^1_\varepsilon )_{\{\zeta \}}(x)}{\partial \zeta }&= -\frac{1}{\zeta ^2}h^1_\varepsilon \left( \frac{x}{\zeta }\right) - \frac{x}{\zeta ^3}(h^1_\varepsilon )'\left( \frac{x}{\zeta }\right) = -\frac{\partial }{\partial x}\left( \frac{x}{\zeta ^2}h^1_\varepsilon \left( \frac{x}{\zeta }\right) \right) \\&= -\frac{\partial }{\partial x}\left( \zeta \frac{x}{\zeta ^2}(h^1_\varepsilon )_{\{\zeta \}}(x)\right) = -\zeta \frac{\partial ^2}{\partial x^2}\left( (H^1_\varepsilon )_{\{\zeta \}}(x)\right) , \end{aligned} \end{aligned}$$

Let now $\zeta (t) = \sin \theta (\tan \theta )^{2t-1}$. We deduce that

$$\begin{aligned} \begin{aligned} \partial _t\mu _t&= \frac{\partial ( h^1_\varepsilon )_{\{\zeta (t)\}}}{\partial t} * {\tilde{h}}^2_\varepsilon * \rho ^2 \\&= \zeta '(t) \frac{\partial ( h^1_\varepsilon )_{\{\zeta (t)\}}}{\partial \zeta } * {\tilde{h}}^2_\varepsilon * \rho ^2 = -\zeta '(t) \zeta (t) [(H^1_\varepsilon )_{\{\zeta (t)\}}]'' * {\tilde{h}}^2_\varepsilon * \rho ^2 \\&= -\zeta '(t) \zeta (t) {{\,\textrm{div}\,}}\left( (H^1_\varepsilon )_{\{\zeta (t)\}} * [(h^2_\varepsilon )_{\{\sin \theta \}}]' * \rho ^2 \right) = {{\,\textrm{div}\,}}(m_t) . \end{aligned} \end{aligned}$$

We can bound by (6.7)

$$\begin{aligned} \Vert {m_t}\Vert _\infty \le C_H \Vert { (H^1_\varepsilon )_{\zeta (t)} }\Vert _1 \cdot \Vert {[(h^2_\varepsilon )_{\{\sin \theta \}}]'}\Vert _1 \cdot \Vert {\rho ^2}\Vert _\infty \le C_H \Vert { H^1_\varepsilon }\Vert _1 \cdot \frac{C}{\beta ^{1/2}} \cdot \Vert {\rho ^2}\Vert _\infty \end{aligned}$$

and estimate the first factor in the right-hand side in terms of powers of $\varepsilon$ using that $h^1_\varepsilon$ is supported in a set of size $C_H \varepsilon ^{1/4} N^{1/2}$

$$\begin{aligned} \begin{aligned} \Vert {H^1_\varepsilon }\Vert _1&\le -2\int _{-\infty }^0 \int _{-\infty }^x r h^1_\varepsilon (r)\textrm{d}r \textrm{d}x = -2\int _{-\infty }^0 \int _{r}^0 {r}h^1_\varepsilon (r)\textrm{d}x \textrm{d}r \\&= 2\int _{-\infty }^0 {r^2}h^1_\varepsilon (r) \textrm{d}r \le C_H \varepsilon ^{1/2}N \Vert {h^1_\varepsilon }\Vert _1 \le C_H \varepsilon ^{1/2}N. \end{aligned} \end{aligned}$$

Hence we obtain (6.12). $\square$

### Wasserstein estimate of Proposition 6.2

#### Proof of Proposition 6.2

The proof is organized as follows: in steps 1 through 3 we prove the $W_2$ estimate (6.4); whereas in step 4 we show how to deduce from this the $L^\infty$ estimate (6.5) for the optimal map, which is our true objective.

Define the map $T_t=(1-t)T+tT_\delta$ and define $T_\delta ^i$ to be the affine extension of ${T_\delta }|_{I^1_i}$. Define also the marginals $\rho ^1_{\varepsilon ,i}$ and $\rho ^2_{\varepsilon ,i}$ of ${\bar{\gamma }}_{\varepsilon ,i}$. We introduce the following three curves of measures:

- $$\rho _t = {T_t}_\#\rho $$ from $$T_\#\rho =\rho $$ to $${T_\delta }_\#\rho $$;
- $\xi ^i_t$ from $\rho ^2_{\varepsilon ,i}$ to ${T_\delta ^i}_\#\rho ^1_{\varepsilon ,i}$ using the construction presented in Proposition 6.3
- $\zeta ^i_t$ from ${T_\delta ^i}_\#\rho ^1_{\varepsilon ,i}$ to ${T_\delta }_\#\rho ^1_{\varepsilon ,i}$ defined by linearly stretching  from $T^i_\delta$ to $T^{i+1}_\delta$ and similarly for  with Lemma 6.4.

We transform the remaining mass in three steps:

$$\begin{aligned}  &   \rho - \rho ^2_\varepsilon \xrightarrow {\rho _t-\rho ^2_\varepsilon } {T_\delta }_\#\rho - \rho ^2_\varepsilon \xrightarrow {{T_\delta }_\#\rho - \sum _i \xi ^i_t} {T_\delta }_\#\rho - \sum _i {T^i_\delta }_\#\rho ^1_{\varepsilon ,i}\\  &   \quad \xrightarrow {{T_\delta }_\#\rho - \sum _i \zeta ^i_t} {T_\delta }_\#\rho - \sum _i {T_\delta }_\#\rho ^1_{\varepsilon ,i} . \end{aligned}$$

We estimate the $W_2$ distance of each step with Benamou-Brenier.

*Step 1.* For every $x\in \mathbb {R}$ we observe that the map $t \rightarrow T_t(x)$ solves the ODE

$$\begin{aligned} \partial _t X(t) = (T_\delta -T) \circ T_t^{-1} \circ X(t) \end{aligned}$$

with initial datum $X(0)= x$. Notice that $(T_\delta -T) \circ T_t^{-1}$ is a locally Lipschitz vector field, therefore $T_t(x)$ represents its flow. As a consequence $\rho _t = (T_t)_\# \rho$ solves

$$\begin{aligned} \partial _t\rho _t=-{{\,\textrm{div}\,}}\bigl ((T_\delta -T)\circ T_t^{-1}\rho _t\bigr ). \end{aligned}$$

From the definition of $\rho _t$ we get that $\rho _t(T_tx) = \rho (x)/T_t'(x)$, whereas $\rho (Tx) = \rho (x)/T'(x)$, since $T_\#\rho =\rho$. These can be rewritten as

$$\begin{aligned} \rho (y)&= \frac{\rho \bigl (T^{-1}(y)\bigr )}{T'\bigl (T^{-1}(y)\bigr )},&\rho _t(y)&= \frac{\rho \bigl (T_t^{-1}(y)\bigr )}{T_t'\bigl (T_t^{-1}(y)\bigr )}. \end{aligned}$$

Our goal is to show that these two densities are close to one another for $y\in \Omega _H$. We start by comparing the denominators. Let $x\in I^1_i$ and $y=T(x)$; we have $T_t^{-1}(y)\in I^1_i$. Then $T'\bigl (T^{-1}(y)\bigr ) = T'(x) \in T'(I^1_i)$; but also $T_t'\bigl (T_t^{-1}(y)\bigr ) = (1-t)T'\bigl (T_t^{-1}(y)\bigr )+tT_\delta '\bigl (T_t^{-1}(y)\bigr ) \in {\textrm{conv}} \bigl (T'(I^1_i)\bigr ) = T'(I^1_i)$. Therefore we have

6.13 $$\begin{aligned}  &   \left|{T_t'\bigl (T_t^{-1}(y)\bigr ) - T'\bigl (T^{-1}(y)\bigr )}\right| \le {{\,\textrm{Lip}\,}}(T') {{\,\textrm{diam}\,}}(I^1_i) \le L\delta ,\nonumber \\  &   \quad \min \{T_t'\bigl (T_t^{-1}(y)\bigr ) : y \in \Omega _{H}\} \ge \min \{T'\bigl (T_t^{-1}(y)\bigr ) : y \in \Omega _{H}\} \ge \frac{1}{L}.\nonumber \\ \end{aligned}$$

Let us now estimate the numerators of the densities. Differentiating with respect to *t* the identity $y = T_t\bigl (T_t^{-1}(y)\bigr )$ and using the fact that $\textrm{d}T_t/\textrm{d}t = T_\delta -T$ we get

$$\begin{aligned} 0 = \frac{\textrm{d}T_t}{\textrm{d}t}\bigl (T_t^{-1}(y)\bigr ) + T_t'\bigl (T_t^{-1}(y)\bigr ) \frac{\textrm{d}T_t^{-1}}{\textrm{d}t}(y), \end{aligned}$$

from which we deduce, by (4.13) and (6.13), that, for $y \in \Omega _H$,

$$\begin{aligned} \left|{\frac{\textrm{d}T_t^{-1}}{\textrm{d}t}(y)}\right| = \left|{\frac{(T-T_\delta )\bigl (T_t^{-1}(y)\bigr )}{T_t'\bigl (T_t^{-1}(y)\bigr )} }\right| \le L^2 \delta ^2. \end{aligned}$$

This allows us to compute for $y \in \Omega _H$ (notice that for $y \in (\Omega _H)^c$ the quantity below is 0) that

$$\begin{aligned} \left|{T_t^{-1}(y) - T^{-1}(y)}\right| = \left|{T_t^{-1}(y) - T_0^{-1}(y)}\right| \le \int _0^t \left|{\frac{\textrm{d}T_t^{-1}}{\textrm{d}t}(y)}\right| \textrm{d}t \le L^2 \delta ^2, \end{aligned}$$

and therefore

$$\begin{aligned} \left|{\rho \bigl (T_t^{-1}(y)\bigr ) - \rho \bigl (T^{-1}(y)\bigr )}\right| \le {{\,\textrm{Lip}\,}}(\rho ) L^2 \delta ^2 \le L^3 \delta ^2 . \end{aligned}$$

From this we conclude that

$$\begin{aligned} \begin{aligned} |{\rho _t(x)- {\rho (x)}}|&\le \frac{|{\rho \bigl (T^{-1}(y)\bigr )-\rho \bigl (T_t^{-1}(y)\bigr )}|}{T'\bigl (T^{-1}(y)\bigr )} + \rho \bigl (T_t^{-1}(y)\bigr )\\&\quad \times \frac{|{T_t'\bigl (T_t^{-1}(y)\bigr )-T'\bigl (T^{-1}(y)\bigr )}|}{T_t'\bigl (T_t^{-1}(y)\bigr )T'\bigl (T^{-1}(y)\bigr )} \\&\le L^4\delta ^2 + L^4\delta \le C_H \delta . \end{aligned} \end{aligned}$$

On the other hand, by (4.18) of Lemma 4.2 we have that $\rho ^2_\varepsilon \le \rho - c_H\tau$.

Combined together, these two density estimates imply that

$$\begin{aligned} C_H \ge \rho _t(x) \ge \rho ^2_\varepsilon (x) + c_H\tau /2 \qquad \text {for every}\, x \in \Omega _H. \end{aligned}$$

Therefore, with Benamou-Brenier and noticing that $T_\delta (y)-T(y) =0$ for $y \in (\Omega _H)^c$, we can compute that

$$\begin{aligned} \begin{aligned} W_2^2(\rho -\rho ^2_\varepsilon , {T_\delta }_\#\rho -\rho ^2_\varepsilon )&\le \int _0^1 \int _{\Omega _H'} \frac{1}{\rho _t(x) - \rho ^2_\varepsilon (x)} \left|{(T_\delta -T)\circ T_t^{-1}(x) \rho _t(x)}\right|^2 \textrm{d}x \textrm{d}t \\&\le \int _0^1 \int _{\Omega _H'} \frac{C_H}{\tau } \Vert {T_\delta -T}\Vert _\infty ^2 \Vert {\rho }\Vert _\infty ^2 \textrm{d}x \textrm{d}t \le \frac{C_H\delta ^4}{\tau }, \end{aligned} \end{aligned}$$

where in the last step we use the estimate (4.13).

*Step 2.* By Proposition 6.3 applied with $\lambda = (\textrm{Id},T_\delta )_\#\bigl ((\rho -\tau ){\varvec{1}}_{I^1_i}\bigr )$ (recall that by (4.15) with this definition $\lambda _\varepsilon$ has marginals $\rho ^1_{\varepsilon ,i}$ and $\rho ^2_{\varepsilon ,i}$) for every *i* there exists a curve $\xi ^i_t$ connecting $\rho ^1_{\varepsilon ,i}$ and $\rho ^2_{\varepsilon ,i}$ such that $\partial _t \xi ^i_t = {{\,\textrm{div}\,}}(m^i_t)$ and

$$\begin{aligned}  &   \Vert {\xi ^i_t - \rho ^2_{\varepsilon ,i}}\Vert _\infty = \Vert {\xi ^i_t - \xi ^i_0}\Vert _\infty \le C_H \frac{\varepsilon ^{1/2}N}{\beta },\\  &   |{m^i_t}| \le C_H \frac{\varepsilon ^{1/2} N }{\beta ^{1/2}}. \end{aligned}$$

The second curve to consider is ${T_\delta }_\#\rho - \sum _i \xi ^i_t$, which solves $\partial _t({T_\delta }_\#\rho -\sum _i\xi ^i_t) = {{\,\textrm{div}\,}}(\sum _i m^i_t)$ and connects ${T_\delta }_\#\rho - \rho ^2_\varepsilon$ and ${T_\delta }_\#\rho - \sum _i {T^i_\delta }_\#\rho ^1_{\varepsilon ,i}$. Since both $\xi ^i_t$ and $m^i_t$ are supported in a small neighborhood of $I^i_\delta$, at each point *x* at most two of each of these objects can overlap. Therefore for a.e. $x\in \Omega _H$

$$\begin{aligned} \left|{\sum _i\xi ^i_t - \rho ^2_\varepsilon }\right| + \left|{\sum _i m^i_t}\right| \le 4 C_H \frac{\varepsilon ^{1/2}N}{\beta ^{1/2}} \end{aligned}$$

because the terms overlap at most twice. This means again that the density of ${T_\delta }_\#\rho - \sum _i \xi ^i_t$ is at least $C_H\tau /2$ and

$$\begin{aligned} \begin{aligned} W_2^2\left( {T_\delta }_\#\rho -\rho ^2_\varepsilon , {T_\delta }_\#\rho -\sum _i{T^i_\delta }_\#\rho ^1_{\varepsilon ,i}\right)&\le \int _0^1 \int _{\Omega _H'} \frac{1}{{T_\delta }_\#\rho - \sum _i \xi ^i_t} \left|{\sum _i m^i_t}\right|^2 \textrm{d}x \textrm{d}t \\&\le \int _0^1 \int _{\Omega _H'} C_H \frac{\varepsilon N^2}{\beta \tau } \textrm{d}x \textrm{d}t \le C_H\frac{\varepsilon N^2}{\beta \tau } . \end{aligned} \end{aligned}$$

*Step 3*. Finally, we deal with the curve ${T_\delta }_\#\rho - \sum _i \zeta ^i_t$ which connects ${T_\delta }_\#\rho - \sum _i {T^i_\delta }_\#\rho ^1_{\varepsilon ,i}$ to ${T_\delta }_\#\rho - \sum _i {T_\delta }_\#\rho ^1_{\varepsilon ,i}$. We split each term $\rho ^1_{\varepsilon ,i}$ as the sum of three contributions

$$\begin{aligned} \rho ^1_{\varepsilon ,i} = \rho ^1_{\varepsilon ,i}{\varvec{1}}_{I^1_{i-1}} + \rho ^1_{\varepsilon ,i}{\varvec{1}}_{I^1_i} + \rho ^1_{\varepsilon ,i}{\varvec{1}}_{I^1_{i+1}} . \end{aligned}$$

On the interval $I^1_i$ the maps $T^i_\delta$ and $T_\delta$ coincide, therefore we leave the central mass $\rho ^1_{\varepsilon ,i}{\varvec{1}}_{I^1_i}$ still. On the interval $I^1_{i+1}$, on the other hand, the map $T_\delta$ coincides with $T^{i+1}_\delta$, so the mass $\rho ^1_{\varepsilon ,i}{\varvec{1}}_{I^1_{i+1}}$ must be transformed from ${T^i_\delta }_\#(\rho ^1_{\varepsilon ,i}{\varvec{1}}_{I^1_{i+1}})$ to ${T^{i+1}_\delta }_\#(\rho ^1_{\varepsilon ,i}{\varvec{1}}_{I^1_{i+1}})$. We perform this transformation by applying Lemma 6.4 to $\mu = \rho ^1_{\varepsilon ,i}{\varvec{1}}_{I^1_{i+1}}$, $x_0$ equal to the point separating $I^1_i$ and $I^1_{i+1}$, $\ell =\sqrt{\beta N}$, $S_{\lambda _0} = T^i_\delta$ and $S_{\lambda _1} = T^{i+1}_\delta$. Similarly, we need to transform the mass in $I^1_{i-1}$ from ${T^i_\delta }_\#(\rho ^1_{\varepsilon ,i}{\varvec{1}}_{I^1_{i-1}})$ to ${T^{i-1}_\delta }_\#(\rho ^1_{\varepsilon ,i}{\varvec{1}}_{I^1_{i-1}})$, and we do so with a mirrored version of Lemma 6.4. In summary, the curve $\zeta ^i_t$ is composed of three terms: a central mass $\rho ^1_{\varepsilon ,i}{\varvec{1}}_{I^1_i}$ which does not move, and two masses one on each side which move according to the construction in Lemma 6.4. Notice that, when summing over *i*, all the contributions in $\sum _i\zeta ^i_t$ deriving from the application of Lemma 6.4 are disjoint, because there are two terms in every $I^1_i$ but they don’t overlap (since $\sqrt{\beta N}\ll \delta$).

The lower bound on the density of ${T_\delta }_\#\rho -\sum _i\zeta ^i_t$ follows from the same estimate as in the first step and the estimate of the density in Lemma 6.4. Moreover, Lemma 6.4 also gives us the estimate on the momentum

$$\begin{aligned} |{m^i_t}| \le \beta ^{1/2}\delta \frac{\max T'}{(\min T')^2} \le L^3 \beta ^{1/2}\delta , \end{aligned}$$

which again do not overlap. Therefore we can compute that

$$\begin{aligned} \begin{aligned}&W_2^2\left( {T_\delta }_\#\rho -\sum _i{T^i_\delta }_\#\rho ^1_{\varepsilon ,i}, {T_\delta }_\#\rho -\sum _i{T_\delta }_\#\rho ^1_{\varepsilon ,i}\right) \\&\quad \le \int _0^1 \int _{\Omega _H'} \frac{1}{{T_\delta }_\#\rho -\sum _i\zeta ^i_t} \left|{\sum _i m^i_t}\right|^2 \textrm{d}x \textrm{d}t \\&\quad \le \int _0^1 \int _{\Omega _H'} \frac{C_H}{\tau } \beta \delta ^2 \textrm{d}x \textrm{d}t \le C_H \frac{\beta \delta ^2}{\tau } . \end{aligned} \end{aligned}$$

*Step 4: proof of* (6.5). We apply the main estimate in [5, Proposition 1.2]: if $\mu _0, \mu _1$ are probability measures on a bounded interval $I_0$, with $\mu _0$ absolutely continuous and with density bounded below by $\tau _0$, then the optimal transport map *S* between $\mu _0$ and $\mu _1$ satisfies the estimate

6.14 $$\begin{aligned} \Vert {S(x) - x}\Vert _{L^\infty }^3 \le \frac{C_\ell }{\tau _0} W_2^2(\mu _0, \mu _1). \end{aligned}$$

Let $S_\varepsilon$ be the optimal map from

$$\begin{aligned} \rho - \rho ^2_\varepsilon = \rho {\varvec{1}}_{\Omega _H'} - \rho ^2_\varepsilon + \rho {\varvec{1}}_{\Omega _H''\setminus \Omega _H} + \rho {\varvec{1}}_{\mathbb {R}\setminus \Omega _H''} \end{aligned}$$

to

$$\begin{aligned} {T_\delta }_\#(\rho -\rho ^1_\varepsilon ) = {T_\delta }_\#(\rho {\varvec{1}}_{\Omega _H'}-\rho ^1_\varepsilon ) + \rho {\varvec{1}}_{\Omega _H''\setminus \Omega _H} + \rho {\varvec{1}}_{\mathbb {R}\setminus \Omega _H''} . \end{aligned}$$

Since the tails outside $\Omega _H''$ of both the marginals coincide with $\rho {\varvec{1}}_{\mathbb {R}\setminus \Omega _H''}$, we claim that $S_\varepsilon (x)=x$ for $x\in \mathbb {R}\setminus \Omega _H''$ and that $S_\varepsilon$ is also an optimal map from

$$\begin{aligned} \mu _0 = (\rho - \rho ^2_\varepsilon ){\varvec{1}}_{\Omega _H''} = (\rho {\varvec{1}}_{\Omega _H'}-\rho ^2_\varepsilon ) + \rho {\varvec{1}}_{\Omega _H''\setminus \Omega _H} \end{aligned}$$

to

$$\begin{aligned} \mu _1 = {T_\delta }_\#(\rho -\rho ^1_\varepsilon ){\varvec{1}}_{\Omega _H''} = {T_\delta }_\#(\rho {\varvec{1}}_{\Omega _H'}-\rho ^1_\varepsilon ) + \rho {\varvec{1}}_{\Omega _H''\setminus \Omega _H}. \end{aligned}$$

Applying (6.14) with $I_0=\Omega _H''$ and $\tau _0 = c_H\tau$ (notice that in the gap $\Omega _H''\setminus \Omega _H'$ we have $\mu _0=\rho \gg \tau$) we deduce that

$$\begin{aligned} \begin{aligned} \Vert {S_\varepsilon (x) - x}\Vert _{L^\infty (\Omega _H'')}^3&\le \frac{C_H}{\tau } W_2^2\left( (\rho - \rho ^2_\varepsilon ){\varvec{1}}_{\Omega _H''}, {T_\delta }_\#(\rho -\rho ^1_\varepsilon ){\varvec{1}}_{\Omega _H''}\right) \\&= \frac{C_H}{\tau } \int _{\Omega _H''} |{S_\varepsilon (x)-x}|^2 (\rho (x)-\rho ^2_\varepsilon (x)) \textrm{d}x \\&\le \frac{C_H}{\tau } \int _\mathbb {R}|{S_\varepsilon (x)-x}|^2 (\rho (x)-\rho ^2_\varepsilon (x)) \textrm{d}x \\&= \frac{C_H}{\tau } W_2^2\left( \rho -\rho ^2_\varepsilon , {T_\delta }_\#(\rho -\rho ^1_\varepsilon )\right) \\&\le C_H\left( \frac{\delta ^4}{\tau ^2} + \frac{\varepsilon N^2}{\beta \tau ^2} + \frac{\varepsilon ^{1/2}N}{\beta \tau }\right) . \end{aligned} \end{aligned}$$

As a final remark, notice that even without invoking (6.14) one could directly verify that the map between $\rho -\rho ^2_\varepsilon$ and ${T_\delta }_\#(\rho -\rho ^1_\varepsilon )$ implicitly constructed above (via the curves of measures proposed in Steps 1,2,3) satisfies an $L^\infty$ estimate of the type (6.5). The proof would meet more technical difficulties than this Step 4, but would however be sufficient. $\square$

#### Lemma 6.4

Let $\mu \in \mathscr {M}_+([x_0,x_0+\ell ])$, $\mu \ll \mathscr {L}^1$, $\lambda _0,\lambda _1\in \mathbb {R}_+$ and $S_\lambda :\mathbb {R}\rightarrow \mathbb {R}:x\mapsto x_0+\lambda (x-x_0)$. Then $\mu _t=(S_{(1-t)\lambda _0+t\lambda _1})_\#\mu$ solves $\partial _t\mu _t={{\,\textrm{div}\,}}(m_t)$ with

$$\begin{aligned} |{m_t}| \le \ell \frac{|{\lambda _1-\lambda _0}|\max \{\lambda _0,\lambda _1\}}{\min \{\lambda _0,\lambda _1\}^2}\Vert {\mu }\Vert _\infty . \end{aligned}$$

Moreover,

$$\begin{aligned} \Vert {\mu _t-\mu _s}\Vert _{L^\infty (0,\infty )} \le C |{\lambda _t-\lambda _s}|+ \ell {{\,\textrm{Lip}\,}}(\mu ) \max \left\{ \frac{\lambda _t}{\lambda _s}, \frac{\lambda _s}{\lambda _t} \right\} . \end{aligned}$$

#### Proof

Without loss of generality, up to translation we may assume $x_0=0$. Let $\lambda _t=(1-t)\lambda _0+t\lambda _1$. Then $\mu _t(x)=\frac{1}{\lambda _t}\mu \left( \frac{x}{\lambda _t}\right)$. The measure $\mu _t$ is advected by the vector field $v_t(x)=\frac{\lambda '_t}{\lambda _t}x=\frac{\lambda _1-\lambda _0}{\lambda _t}x$: in fact

$$\begin{aligned} \partial _t\mu _t + {{\,\textrm{div}\,}}(v_t\mu _t) = \frac{\partial }{\partial t}\left[ \frac{1}{\lambda _t}\mu \left( \frac{x}{\lambda _t}\right) \right] + \frac{\partial }{\partial x} \left[ \frac{\lambda '_t}{\lambda _t^2}x\mu \left( \frac{x}{\lambda _t}\right) \right] = 0. \end{aligned}$$

Therefore $$m_t=-v_t\mu _t$$ and

$$\begin{aligned} |{m_t}| \le \ell \frac{|{\lambda _1-\lambda _0}|\max \{\lambda _0,\lambda _1\}}{\min \{\lambda _0,\lambda _1\}^2}\Vert {\mu }\Vert _\infty \end{aligned}$$

because $$|{x}| < \max {\lambda _0,\lambda _1}\ell $$ where $$\mu (x/\lambda _t)>0$$.

The $L^\infty$ estimate comes from the explicit formula for the density of $\mu _t$. $\square$

### Kinetic energy of the remaining mass

#### Proof of Proposition 6.1(ii)

We start by showing the bound for ${{\,\textrm{KE}\,}}(\rho -\rho ^1_\varepsilon )$. We split the kinetic energy in the domain $S={{\,\textrm{supp}\,}}\rho ^1_\varepsilon \subset \Omega _H'$ and the complement, making use of (4.18) of Lemma 4.2 and the fact that in $S^c=({{\,\textrm{supp}\,}}\rho ^1_\varepsilon )^c$ we have $\rho -\rho ^1_\varepsilon =\rho$. What we obtain is

$$\begin{aligned} \begin{aligned} {{\,\textrm{KE}\,}}(\rho -\rho ^1_\varepsilon )&= \int _{S} \frac{|{\partial _x[\rho (x)-\rho ^1_\varepsilon (x)]}|^2}{8[\rho (x)-\rho ^1_\varepsilon (x)]} \textrm{d}x \\&= \int _{S} \frac{|{\partial _x[\rho (x)-\rho ^1_\varepsilon (x)]}|^2}{8[\rho (x)-\rho ^1_\varepsilon (x)]} \textrm{d}x + \int _{S^c} \frac{|{\partial _x[\rho (x)-\rho ^1_\varepsilon (x)]}|^2}{8[\rho (x)-\rho ^1_\varepsilon (x)]} \textrm{d}x \\&\le \frac{C_H}{\tau } \int _{S} \left( |{\partial _x\rho (x)}|^2 + |{\partial _x\rho ^1_\varepsilon (x)}|^2 \right) \textrm{d}x + \int _{S^c} \frac{|{\partial _x \rho (x)}|^2}{4\rho (x)} \textrm{d}x \\&\le \frac{C_H\Vert {\rho }\Vert _{L^\infty (\Omega _H')}}{\tau } \int _{S} \frac{|{\partial _x\rho (x)}|^2}{4\rho (x)} \textrm{d}x + \frac{C_H}{\tau } \int _{S} |{\partial _x\rho ^1_\varepsilon (x)}|^2 \textrm{d}x \\&\quad + \int _{S^c} \frac{|{\partial _x \rho (x)}|^2}{4\rho (x)} \textrm{d}x \\&\le \frac{C_HL}{\tau } {{\,\textrm{KE}\,}}(\rho ) + \frac{C_H}{\tau } \int _{S} |{\partial _x\rho ^1_\varepsilon (x)}|^2 \textrm{d}x . \end{aligned} \end{aligned}$$

Let us recall that the domain $\Omega _H$ defined in (4.5) has two connected components which are both covered by an essentially disjoint family of intervals $I^1_i=[a_i,b_i]$ of length $\delta /2<b_i-a_i<\delta$ with

$$\begin{aligned} T(r_H) = a_1< b_1 = a_2< \dots< b_{k_0} = T(H) , \\ r_H = a_{k_0+1}< b_{k_0+1} = a_{k_0+2}< \dots < b_{k_1} = H . \end{aligned}$$

Notice that the cardinality of the intervals is $k_1 \le C_H/\delta$. As already mentioned in (4.20), we have that

$$\begin{aligned} \rho ^1_\varepsilon (x) = \sum _{i=1}^{k_1} \Bigl (\bigl [(\rho -\tau ){\varvec{1}}_{I^1_i}\bigr ]*\eta ^1_{\varepsilon ,i}\Bigr )(x), \end{aligned}$$

where $\eta ^1_{\varepsilon ,i} = {{\,\textrm{proj}\,}}^1_\# \Gamma _{M_{\varepsilon ,\beta }(x_i),N}$ were first introduced in the proof of Lemma 4.2. As a consequence, using the fact that the terms in the sum overlap at most twice, we have

$$\begin{aligned} \begin{aligned} \Vert {\partial _x\rho ^1_\varepsilon }\Vert _2^2&= \left\Vert {\sum _{i=1}^{k_1} \bigl [(\rho -\tau ){\varvec{1}}_{I^1_i}\bigr ] * \partial _x\eta ^1_{\varepsilon ,i}}\right\Vert _2^2 \\&\le 2\sum _{i=1}^{k_1} \left\Vert {\bigl [(\rho -\tau ){\varvec{1}}_{I^1_i}\bigr ] * \partial _x\eta ^1_{\varepsilon ,i}}\right\Vert _2^2 \le 2\sum _{i=1}^{k_1} \left\Vert {(\rho -\tau ){\varvec{1}}_{I^1_i}}\right\Vert _2^2 \Vert {\partial _x\eta ^1_{\varepsilon ,i}}\Vert _1^2 . \end{aligned} \end{aligned}$$

Using the representation (6.8) we see that the function $\eta ^1_{\varepsilon ,i}$ is increasing in $(-\infty ,0]$, decreasing in $[0,\infty )$ and vanishes at infinity, hence

$$\begin{aligned} \begin{aligned} \Vert {\partial _x\eta ^1_{\varepsilon ,i}}\Vert _1&= 2\Vert {\eta ^1_{\varepsilon ,i}}\Vert _\infty \le 2\Vert {(h^1_\varepsilon )_{\{\sin \theta \}}}\Vert _1 \Vert {(h^2_\varepsilon )_{\{\cos \theta \}}}\Vert _\infty \\&= 2(\cos \theta )^{-1} \Vert {h^2_\varepsilon }\Vert _\infty = 2(\cos \theta )^{-1} G_{\frac{1}{\beta },N}^{-1} \le C_H \beta ^{-1/2}. \end{aligned} \end{aligned}$$

As a consequence,

$$\begin{aligned} \Vert {\partial _x\rho ^1_\varepsilon }\Vert _2^2\le &   \frac{C_H}{\beta } \sum _{i=1}^{k_1} \left\Vert {(\rho -\tau ){\varvec{1}}_{I^1_i}}\right\Vert _2^2 = \frac{C_H}{\beta } \Vert {\rho -\tau }\Vert _{L^2(\Omega _H)}^2\\\le &   \frac{C_H}{\beta } \Vert {\rho }\Vert _1 \Vert {\rho }\Vert _{L^\infty (\Omega _H')} \le \frac{C_H}{\beta } . \end{aligned}$$

Using this inequality in the original estimate of the kinetic energy we finally get

$$\begin{aligned} {{\,\textrm{KE}\,}}(\rho -\rho ^1_\varepsilon ) \le \frac{C_H}{\tau } {{\,\textrm{KE}\,}}(\rho ) + \frac{C_H}{\tau \beta } . \end{aligned}$$

Let us now move on to the analogous estimate for ${{\,\textrm{KE}\,}}(\rho -\rho ^2_\varepsilon )$. With the same argument as we did at the beginning, splitting the kinetic energy in the domain $S={{\,\textrm{supp}\,}}\rho ^2_\varepsilon \subset \Omega _H'$ and the complement we get

$$\begin{aligned} {{\,\textrm{KE}\,}}(\rho -\rho ^2_\varepsilon ) \le \frac{C_H}{\tau } {{\,\textrm{KE}\,}}(\rho ) + \frac{C_H}{\tau } \int _\mathbb {R}|{\partial _x\rho ^2_\varepsilon (x)}|^2 \textrm{d}x . \end{aligned}$$

As already mentioned in the proof of (4.18) of Lemma 4.2, we have that

$$\begin{aligned} \rho ^2_\varepsilon (y) = \sum _{i=1}^{k_1} \Bigl (\bigl [{T_\delta }_\#(\rho -\tau ){\varvec{1}}_{I^2_i}\bigr ]*\eta ^2_{\varepsilon ,i}\Bigr )(y) , \end{aligned}$$

where $\eta ^2_{\varepsilon ,i} = {{\,\textrm{proj}\,}}^2_\#\Gamma _{M_{\varepsilon ,\beta }(x_i),N}$ are the second marginals of the Gaussians. The intervals $[c_i,d_i]=I^2_i=T_\delta (I^1_i)=T(I^1_i)=[T(a_i),T(b_i)]$ have lengths $\delta /(2L)\le {{\,\textrm{diam}\,}}(I^2_i)\le L\delta$. If $y\in I^2_i$ we have that

$$\begin{aligned} {T_\delta }_\#(\rho -\tau )(y) = [\rho (T_\delta ^{-1}(y))-\tau ]/T'(x_i) \le L \Vert {\rho }\Vert _{L^\infty (\Omega _H')} ; \end{aligned}$$

on the other hand, from (6.9), we have

$$\begin{aligned} \begin{aligned} \Vert {\eta ^2_{\varepsilon ,i}}\Vert _\infty \le \Vert {(h^1_\varepsilon )_{\{\cos \theta \}}}\Vert _1 \Vert {(h^2_\varepsilon )_{\{\sin \theta \}}}\Vert _\infty \le (\sin \theta )^{-1}\Vert {h^2_\varepsilon }\Vert _\infty \le C_H \beta ^{-1/2}, \end{aligned} \end{aligned}$$

and therefore

$$\begin{aligned} \Vert {\partial _y \eta ^2_{\varepsilon ,i}}\Vert _1 = 2\Vert {\eta ^2_{\varepsilon ,i}}\Vert _\infty \le C_H \beta ^{-1/2} . \end{aligned}$$

This implies that

$$\begin{aligned} \begin{aligned} \Vert {\partial _y \rho ^2_\varepsilon }\Vert _2^2&= \left\Vert {\sum _{i=1}^{k_1} \bigl [{T_\delta }_\#(\rho -\tau ){\varvec{1}}_{I^2_i}\bigr ] * \partial _y \eta ^2_{\varepsilon ,i}}\right\Vert _2^2 \le 2\sum _{i=1}^{k_1} \left\Vert {\bigl [{T_\delta }_\#(\rho -\tau ){\varvec{1}}_{I^2_i}\bigr ] * \partial _y \eta ^2_{\varepsilon ,i}}\right\Vert _2^2 \\&\le 2\sum _{i=1}^{k_1} \left\Vert {{T_\delta }_\#(\rho -\tau ){\varvec{1}}_{I^2_i}}\right\Vert _2^2 \Vert {\partial _y\eta ^2_{\varepsilon ,i}}\Vert _1^2 \le \frac{C_H}{\beta } \sum _{i=1}^{k_1} \left\Vert {{T_\delta }_\#\rho {\varvec{1}}_{I^2_i}}\right\Vert _1 \left\Vert {{T_\delta }_\#\rho {\varvec{1}}_{I^2_i}}\right\Vert _\infty \\&\le \frac{C_H}{\beta } L \Vert {\rho }\Vert _{L^\infty (\Omega _H')} \sum _{i=1}^{k_1} \left\Vert {\rho {\varvec{1}}_{I^2_i}}\right\Vert _1 \le \frac{C_H}{\beta } L \Vert {\rho }\Vert _{L^\infty (\Omega _H')} \left\Vert {\rho }\right\Vert _{L^1(\Omega _H')} \le \frac{C_H}{\beta } , \end{aligned} \end{aligned}$$

which, inserted into the original estimate of the kinetic energy, leads to

$$\begin{aligned} {{\,\textrm{KE}\,}}(\rho -\rho ^2_\varepsilon ) \le \frac{C_H}{\tau } {{\,\textrm{KE}\,}}(\rho ) + \frac{C_H}{\tau \beta }. \end{aligned}$$

$$\square $$

## Proof of Theorems 1.1 and 1.3

### Proof of Theorem 1.3

Given $H>1$ and $L=L(H)$ defined by (4.7), let ${\bar{\gamma }}_\varepsilon$ be as defined in (4.14), built with admissible parameters $N,\beta ,\delta ,\tau$ satisfying (4.8)–(4.11), for instance the explicit choice (4.12). By Lemma 4.2 we have that

$$\begin{aligned} \limsup _{\varepsilon \rightarrow 0} E_\varepsilon (\sqrt{{\bar{\gamma }}_\varepsilon }) \le \frac{1}{2} \int _{\Omega _H} {{\,\textrm{tr}\,}}\left( \sqrt{\nabla ^2V(x, T(x))}\right) \rho (x) \textrm{d}x \le \frac{1}{2} \int _{\mathbb {R}^2} {{\,\textrm{tr}\,}}\bigl (\sqrt{D^2V}\bigr ) \textrm{d}\gamma . \end{aligned}$$

The problem is that ${\bar{\gamma }}_\varepsilon$ is not a recovery sequence because it has the wrong mass and marginals, so we have to use the remaining mass to fix the marginals.

For $i=1,2$, let $\upsilon ^i_\varepsilon = {{\,\textrm{proj}\,}}^i_\#(\gamma ) - {{\,\textrm{proj}\,}}^i_\#({\bar{\gamma }}_\varepsilon ) = \rho - \rho ^i_\varepsilon$, which is a positive measure as remarked in (4.18) of Lemma 4.2.

Let $\pi _\varepsilon \in \Pi (\upsilon ^1_\varepsilon ,\upsilon ^2_\varepsilon )$ be the plan given by Proposition 6.1(i). Notice that, by (4.19), $\Vert {\upsilon ^i_\varepsilon }\Vert _1=\Vert {\pi _\varepsilon }\Vert _1\le C_H\tau + \rho (\Omega _H^c)$. With *H* fixed the first term goes to 0 when $\varepsilon \rightarrow 0$, whereas the second term goes to zero as $H\rightarrow \infty$.

Let ${\tilde{\pi }}_\varepsilon \in \Pi (\upsilon ^1_\varepsilon ,\upsilon ^2_\varepsilon )$ be the deconvolved plan defined in Sect.  5 starting from $\Pi _0=\pi _\varepsilon$ (whose marginals are $\sigma ^1=\upsilon ^1_\varepsilon$ and $\sigma ^2=\upsilon ^2_\varepsilon$) . By Proposition 5.1 (notice that the assumption (5.4) is satisfied because of (6.2)) and the bound on the mass of $\pi _\varepsilon$ we have

$$\begin{aligned} \begin{aligned} E_\varepsilon (\sqrt{{\tilde{\pi }}_\varepsilon })&= \varepsilon ^{1/2}{{\,\textrm{KE}\,}}({\tilde{\pi }}_\varepsilon ) + \varepsilon ^{-1/2} \int _{\mathbb {R}^2} V\textrm{d}{\tilde{\pi }}_\varepsilon \\&\le \varepsilon ^{1/2}[{{\,\textrm{KE}\,}}(\upsilon ^1_\varepsilon )+{{\,\textrm{KE}\,}}(\upsilon ^2_\varepsilon )] + C_H\varepsilon ^{-1/2} \int _{\mathbb {R}^2} V\textrm{d}\pi _\varepsilon + C\Vert {\pi _\varepsilon }\Vert _1 \\&\le \varepsilon ^{1/2}[{{\,\textrm{KE}\,}}(\upsilon ^1_\varepsilon )+{{\,\textrm{KE}\,}}(\upsilon ^2_\varepsilon )] + C_H\varepsilon ^{-1/2}\int _{\mathbb {R}^2} V\textrm{d}\pi _\varepsilon + C_H\tau + C\rho (\Omega _H^c). \end{aligned} \end{aligned}$$

By Proposition 6.1, we have $\lim _{\varepsilon \rightarrow 0}\varepsilon ^{-1/2}\int _{\mathbb {R}^2} V\textrm{d}\pi _\varepsilon =0= \lim _{\varepsilon \rightarrow 0}\varepsilon ^{1/2}{{\,\textrm{KE}\,}}(\upsilon ^i_\varepsilon )$, and therefore

7.1 $$\begin{aligned} \lim _{\varepsilon \rightarrow 0}E_\varepsilon (\sqrt{{\tilde{\pi }}_\varepsilon }) \le C\rho (\Omega _H^c). \end{aligned}$$

Finally, let $\psi _\varepsilon = \sqrt{{\bar{\gamma }}_\varepsilon +{\tilde{\pi }}_\varepsilon }$; then $\psi _\varepsilon$ has the correct marginals to be a recovery sequence for $\gamma$. Moreover, by the subadditivity of $E_\varepsilon (\sqrt{\gamma })$ with respect to $\gamma$ and (7.1), we have

$$\begin{aligned}\begin{aligned} \limsup _{\varepsilon \rightarrow 0} E_\varepsilon (\psi _\varepsilon )&\le \limsup _{\varepsilon \rightarrow 0} [E_\varepsilon (\sqrt{{\bar{\gamma }}_\varepsilon }) + E_\varepsilon (\sqrt{{\tilde{\pi }}_\varepsilon })] \\&\le \limsup _{\varepsilon \rightarrow 0} E_\varepsilon (\sqrt{{\bar{\gamma }}_\varepsilon }) + C\rho (\Omega _H^c)\\&\le \frac{1}{2} \int _{\mathbb {R}^2} {{\,\textrm{tr}\,}}\bigl (\sqrt{D^2V}\bigr ) \textrm{d}\gamma + C\rho (\Omega _H^c). \end{aligned} \end{aligned}$$

We now use a diagonal argument to conclude. For every $n\in \mathbb {N}_+$ we can find $H_n$ large enough such that $C\rho (\Omega _{H_n}^c)<1/n$. We can then find $\varepsilon _n$ such that the $\psi _{\varepsilon _n}$ constructed as above with parameters $H_n,\varepsilon _n,\beta _n,\delta _n,\tau _n,N_n$ satisfies

$$\begin{aligned} E_{\varepsilon _n}(\psi _{\varepsilon _n})\le &   \frac{1}{2} \int _{\mathbb {R}^2} {{\,\textrm{tr}\,}}\bigl (\sqrt{D^2V}\bigr ) \textrm{d}\gamma + C\rho (\Omega _{H_n}^c) + 1/n \\\le &   \frac{1}{2} \int _{\mathbb {R}^2} {{\,\textrm{tr}\,}}\bigl (\sqrt{D^2V}\bigr ) \textrm{d}\gamma + 2/n, \end{aligned}$$

which is precisely the thesis of the theorem. $\square$

### Proof of Theorem 1.1

To prove (1.4), we take a sequence $\psi _\varepsilon$ which almost realizes the minimum in (1.6) up to a vanishing error. Up to subsequence, $|{\psi _\varepsilon }|^2\rightharpoonup \gamma$ where $\gamma \in \Pi _0(\rho )$ with $\gamma (\{V>0\})=0$. Applying Theorem 1.2 to this sequence we get

$$\begin{aligned} \liminf _{\varepsilon \rightarrow 0} \frac{F^\varepsilon (\rho )-F_{OT}(\rho )}{\sqrt{\varepsilon }} \ge \liminf _{\varepsilon \rightarrow 0} E_\varepsilon (\psi _\varepsilon ) \ge \frac{1}{2} \int _{\mathbb {R}^{dN}} {{\,\textrm{tr}\,}}\left( \sqrt{D^2V}\right) \textrm{d}\gamma = F_{ZPO}(\rho ) . \end{aligned}$$

To prove (1.5), we take $\gamma \in \Pi _0(\rho )$ with $\gamma (\{V>0\})=0$ and $F_{ZPO}(\rho ) = \frac{1}{2} \int _{\mathbb {R}^{dN}} {{\,\textrm{tr}\,}}\left( \sqrt{D^2V}\right) \textrm{d}\gamma$. If $\psi _\varepsilon$ is a recovery sequence given by Theorem 1.3, we get

$$\begin{aligned} \limsup _{\varepsilon \rightarrow 0} \frac{F^\varepsilon (\rho )-F_{OT}(\rho )}{\sqrt{\varepsilon }} \le \limsup _{\varepsilon \rightarrow 0} E_\varepsilon (\psi _\varepsilon ) \le F_{ZPO}(\rho ). \end{aligned}$$

$$\square $$

## Appendix: proof of Lemmas 3.1 and 3.2

### Proof of Lemma 3.1

Notice that $G_{M,N}<G_{M,\infty }$ by definition, so we only need to prove the second inequality of (3.2). We have $G_{\alpha ,N}<G_{\alpha ,\infty }$ and we claim that

7.2 $$\begin{aligned} G_{\alpha ,\infty } - G_{\alpha ,N} \le 3e^{-N/2} G_{\alpha ,\infty } . \end{aligned}$$

By a change of variable we reduce to prove it only for $\alpha =1$. We apply the pointwise inequality

7.3 $$\begin{aligned} f^2\le (f-e^{-N/2})^2 + 2fe^{-N/2} \end{aligned}$$

to $f= e^{-t^2/2}$ for $t< \sqrt{N}$ and use $N\ge 1$ to get

$$\begin{aligned} \begin{aligned} G_{1,\infty }&= \int _{\{|{t}|\le \sqrt{N}\}} e^{-t^2} \textrm{d}t + \int _{\{|{t}|>\sqrt{N}\}} e^{-t^2} \textrm{d}t \\&\le \int _{\{|{t}|\le \sqrt{N}\}} \left[ \left( e^{- t^2/2}-e^{-N/2}\right) ^2 + 2e^{-t^2/2}e^{-N/2} \right] \textrm{d}t + \int _{\{|{t}|> \sqrt{N}\}} t e^{- t^2} \textrm{d}t \\&\le G_{1,N} + 2 \sqrt{2\pi }e^{-N/2} + e^{-N} = G_{1,N} + \left( 2\sqrt{2} + \frac{e^{-N/2}}{\sqrt{\pi }}\right) e^{-N/2} G_{1,\infty } , \end{aligned} \end{aligned}$$

which proves the claim (7.2) for $N\ge 3$, because the factor inside parentheses is less than 3. From this, (3.2) follows because

$$\begin{aligned} \begin{aligned} G_{M,\infty } - G_{M,N}&= G_{a,\infty } (G_{b,\infty }-G_{b,N}) + G_{b,N} (G_{a,\infty } - G_{a,N}) \\&\le G_{a,\infty }3e^{-N/2}G_{b,\infty } + G_{b,N}3e^{-N/2}G_{a,\infty } \\&\le 6e^{-N/2}G_{a,\infty }G_{b,\infty } = 6e^{-N/2}G_{M,\infty } . \end{aligned} \end{aligned}$$

As a consequence, we also get that

7.4 $$\begin{aligned} \frac{1}{G_{M,N}}-\frac{1}{G_{M,\infty }} = \frac{G_{M,\infty }-G_{M,N}}{G_{M,\infty }G_{M,N}} \le \frac{6e^{-N/2}}{G_{M,N}} \le \frac{6e^{-N/2}}{(1-6e^{-N/2})G_{M,\infty }} \le \frac{7e^{-N/2}}{G_{M,\infty }} . \end{aligned}$$

Now let $f=e^{-aw^2/2}$ and $g=e^{-bz^2/2}$. Notice that $f,g\le 1$ and that we can apply (7.3) to *f* and *g*. Therefore,

$$\begin{aligned} \begin{aligned} \left|{{\tilde{\Gamma }}_{M,\infty }({{\varvec{x}}})-{\tilde{\Gamma }}_{M,N}({{\varvec{x}}})}\right|&= f^2g^2 - \bigl (f-e^{-N/2}\bigr )_+^2 \bigl (g-e^{-N/2}\bigr )_+^2 \\&= f^2 \left( g^2 - \bigl (g-e^{-N/2}\bigr )_+^2\right) + \left( f^2 - \bigl (f-e^{-N/2}\bigr )_+^2\right) \\&\quad \, \bigl (g-e^{-N/2}\bigr )_+^2 \\&\le 2 (f^2g+fg^2) e^{-N/2} \le 4 e^{-N/2}. \end{aligned} \end{aligned}$$

We can now use this information and (7.4) to estimate the difference of the two normalized Gaussians as

$$\begin{aligned} \begin{aligned} \Vert {\Gamma _{M,\infty }-\Gamma _{M,N}}\Vert _\infty&= \left\Vert { \frac{{\tilde{\Gamma }}_{M,\infty } - {\tilde{\Gamma }}_{M,N}}{G_{M,\infty }} - {\tilde{\Gamma }}_{M,N} \left( \frac{1}{G_{M,N}} - \frac{1}{G_{M,\infty }}\right) }\right\Vert _\infty \\&= \frac{\Vert {{\tilde{\Gamma }}_{M,\infty }(x)-{\tilde{\Gamma }}_{M,N}(x)}\Vert _\infty }{G_{M,\infty }} + \Vert {{\tilde{\Gamma }}_{M,N}}\Vert _\infty \left( \frac{1}{G_{M,N}} - \frac{1}{G_{M,\infty }}\right) \\&\le \frac{4e^{-N/2}}{G_{M,\infty }} + \frac{7e^{-N/2}}{G_{M,\infty }} = \frac{11e^{-N/2}}{G_{M,\infty }} = \frac{11}{\pi }\sqrt{\det M} e^{-N/2} , \end{aligned} \end{aligned}$$

proving (3.3). To prove the $L^1$ estimate (3.4), we divide the integral on ${{\,\textrm{supp}\,}}\Gamma _{M,N}$ and the complement. In the first, thanks to (3.3), we obtain

$$\begin{aligned} \begin{aligned} \Vert {\Gamma _{M,N}-\Gamma _{M,\infty }}\Vert _{L^1({{\,\textrm{supp}\,}}\Gamma _{M,N})}&\le \Vert {\Gamma _{M,N}-\Gamma _{M,\infty }}\Vert _\infty \cdot |{{{\,\textrm{supp}\,}}\Gamma _{M,N}}|\\&\le C\sqrt{\det M}e^{-N/2} \frac{4N}{\sqrt{ab}} \le C e^{-N/2} N . \end{aligned} \end{aligned}$$

In the complement, only $\Gamma _{M,\infty }$ is nonzero, and by (3.2) we have that

$$\begin{aligned} \begin{aligned}&\Vert {\Gamma _{M,N}-\Gamma _{M,\infty }}\Vert _{L^1(({{\,\textrm{supp}\,}}\Gamma _{M,N})^c)} \\&\quad = \int _{({{\,\textrm{supp}\,}}\Gamma _{M,N})^c} \Gamma _{M,\infty }({{\varvec{x}}}) \textrm{d}{{\varvec{x}}}= 1 - \int _{{{\,\textrm{supp}\,}}\Gamma _{M,N}} \Gamma _{M,\infty }({{\varvec{x}}}) \textrm{d}{{\varvec{x}}}\\&\quad \le 1 - \int _{{{\,\textrm{supp}\,}}\Gamma _{M,N}} \frac{{\tilde{\Gamma }}_{M,N}({{\varvec{x}}})}{G_{M,\infty }} \textrm{d}{{\varvec{x}}}= 1 - \frac{G_{M,N}}{G_{M,\infty }} \le C e^{-N/2}N . \end{aligned} \end{aligned}$$

Let us now prove (3.5). Define $R_x$ to be the section above *x* of the rectangle ${{\,\textrm{supp}\,}}\Gamma _{M,N}$, i.e.

$$\begin{aligned} R_x = \{{y}\}{(x,y)\in {{\,\textrm{supp}\,}}\Gamma _{M,N}} = \{{y}\}{aw^2\le N,\ bz^2\le N}. \end{aligned}$$

Since, $b y^2 \le b(w^2+z^2) \le aw^2+bz^2 \le a(w^2+z^2) = a(x^2+y^2)$, we have that

$$\begin{aligned} \{{(x,y)\in \mathbb {R}^2}\}{a(x^2+y^2)\le N} \subset {{\,\textrm{supp}\,}}\Gamma _{M,N} \end{aligned}$$

and

$$\begin{aligned} R_x \subseteq \{{y}\}{by^2\le 2N} ,\qquad R_x^c \subseteq \{{y}\}{ay^2 > N-ax^2}. \end{aligned}$$

Therefore, using (3.3), we can estimate that

$$\begin{aligned} \begin{aligned} |{\eta _{M,\infty }(x) - \eta _{M,N}(x)}|&\le \int _\mathbb {R}|{\Gamma _{M,\infty }(x,y)-\Gamma _{M,N}(x,y)}| \textrm{d}y \\&= \int _{R_x} |{\Gamma _{M,\infty }(x,y)-\Gamma _{M,N}(x,y)}| \textrm{d}y + \int _{R_x^c} \Gamma _{M,\infty }(x,y) \textrm{d}y . \\ \end{aligned} \end{aligned}$$

The first integral can be estimated with (3.3) as

$$\begin{aligned} \begin{aligned} \int _{R_x} |{\Gamma _{M,\infty }(x,y)-\Gamma _{M,N}(x,y)}| \textrm{d}y&\le |{R_x}| \cdot \Vert {\Gamma _{M,\infty }-\Gamma _{M,N}}\Vert _\infty \\&\le \left( 2\sqrt{2}\frac{\sqrt{N}}{\sqrt{b}}\right) \left( C\sqrt{ab}e^{-N/2}\right) \\&\le C\sqrt{a} \sqrt{N} e^{-N/2} . \end{aligned} \end{aligned}$$

For the second integral we proceed as follows. Let $B=B(0,\sqrt{N/b}) = \{b(w^2+z^2)<N\} = \{b(x^2+y^2)<N\}$. Note that in $R^c_x$ we have $e^{-aw^2-bz^2}<e^{-N}$ because at least one among $aw^2>N$ and $bz^2>N$ is true. As a consequence, we have that

$$\begin{aligned} \int _{\mathbb {R}^c_x\cap B} e^{-aw^2-bz^2}\textrm{d}y \le e^{-N} {{\,\textrm{diam}\,}}(B) = \frac{2}{\sqrt{b}}e^{-N} \sqrt{N}, \end{aligned}$$

while on the other hand,

$$\begin{aligned} \begin{aligned} \int _{\mathbb {R}^c_x\setminus B} e^{-aw^2-bz^2}\textrm{d}y&\le \int _{\mathbb {R}^c_x\setminus B} e^{-bw^2-bz^2} \textrm{d}y = \int _{\{by^2>N-bx^2\}} e^{-bx^2-by^2} \textrm{d}y \\&= \frac{\sqrt{\pi }}{\sqrt{b}} e^{-bx^2} {{\,\textrm{erfc}\,}}\left( \sqrt{N-bx^2}\right) \\&\le \frac{\sqrt{\pi }}{\sqrt{b}} e^{-bx^2} e^{-N+bx^2} = \frac{\sqrt{\pi }}{\sqrt{b}} e^{-N} , \end{aligned} \end{aligned}$$

and therefore

$$\begin{aligned} \begin{aligned} \int _{\mathbb {R}^c_x} e^{-aw^2-bz^2}\textrm{d}y = \int _{\mathbb {R}^c_x\cap B} e^{-aw^2-bz^2}\textrm{d}y + \int _{\mathbb {R}^c_x\setminus B} e^{-aw^2-bz^2}\textrm{d}y \le \frac{C}{\sqrt{b}} e^{-N}\sqrt{N} . \end{aligned} \end{aligned}$$

Hence,

$$\begin{aligned} \int _{R_x^c} \Gamma _{M,\infty }(x,y) \textrm{d}y= &   G_{M,\infty }^{-1} \int _{R_x^c} e^{-(aw^2+bz^2)} \textrm{d}y \le \frac{\sqrt{ab}}{\pi } \frac{C}{\sqrt{b}} e^{-N}\sqrt{N}\\\le &   C \sqrt{a} e^{-N}\sqrt{N}. \end{aligned}$$

In conclusion, putting the two estimates together, we have

$$\begin{aligned} |{\eta _{M,\infty }(x) - \eta _{M,N}(x)}| \le C\sqrt{a} \sqrt{N} e^{-N/2} . \end{aligned}$$

$$\square $$

### Proof of Lemma 3.2

In some computations of this proof we use the standard error function and the complementary error function

$$\begin{aligned} {{\,\textrm{erf}\,}}(z)&= \frac{2}{\sqrt{\pi }} \int _0^z e^{-t^2}\textrm{d}t,&{{\,\textrm{erfc}\,}}(z)&= \frac{2}{\sqrt{\pi }} \int _z^\infty e^{-t^2}\textrm{d}t = 1-{{\,\textrm{erf}\,}}(z). \end{aligned}$$

The crucial property that we will need is the fast decay at infinity of ${{\,\textrm{erfc}\,}}$ implied by the bound

$$\begin{aligned} {{\,\textrm{erfc}\,}}(z) \le \frac{2}{\sqrt{\pi }} \int _z^\infty \frac{t}{z}e^{-t^2}\textrm{d}t \le \frac{e^{-z^2}}{\sqrt{\pi }z} . \end{aligned}$$

Since

$$\begin{aligned} ({{\,\textrm{supp}\,}}\Gamma _{M,N})^c \subset \{|{w}|>\sqrt{N/a}\} \cup \{|{z}|>\sqrt{N/b}\}, \end{aligned}$$

with the aid of (3.4) we can estimate that

$$\begin{aligned} \begin{aligned} \int _{\mathbb {R}^2}&|{M{{\varvec{x}}}}|^2 |{\Gamma _{M,N}({{\varvec{x}}})-\Gamma _{M,\infty }({{\varvec{x}}})}| \textrm{d}{{\varvec{x}}}\\&\le \int _{{{\,\textrm{supp}\,}}\Gamma _{M,N}} |{M{{\varvec{x}}}}|^2 |{\Gamma _{M,N}({{\varvec{x}}})-\Gamma _{M,\infty }({{\varvec{x}}})}| \textrm{d}{{\varvec{x}}}\\&\quad + \int _{({{\,\textrm{supp}\,}}\Gamma _{M,N})^c} |{M{{\varvec{x}}}}|^2 \Gamma _{M,\infty }({{\varvec{x}}}) \textrm{d}{{\varvec{x}}}\\&\le \left( \sup _{{{\,\textrm{supp}\,}}\Gamma _{M,N}} |{M{{\varvec{x}}}}|^2 \right) \Vert {\Gamma _{M,N}-\Gamma _{M,\infty }}\Vert _1\\&\quad + G_{M,\infty }^{-1} 2\int _{\sqrt{N/a}}^\infty \int _\mathbb {R}(a^2w^2+b^2z^2) e^{-aw^2-bz^2} \textrm{d}z \textrm{d}w\\&\quad + G_{M,\infty }^{-1} 2\int _{\sqrt{N/b}}^\infty \int _\mathbb {R}(a^2w^2+b^2z^2) e^{-aw^2-bz^2} \textrm{d}w \textrm{d}z \\&\le (a+b)CNe^{-N/2} + (a+b)\left( {{\,\textrm{erfc}\,}}(\sqrt{\pi }) + \frac{\sqrt{N}}{\sqrt{\pi }} e^{-N} \right) \\&\le C{{\,\textrm{tr}\,}}(M) N e^{-N/2} . \end{aligned} \end{aligned}$$

For the kinetic energy, we can compute that

7.5 $$\begin{aligned} \begin{aligned} \nabla _{w,z}\sqrt{\Gamma _{M,N}({{\varvec{x}}})}&= G_{M,N}^{-1/2} \begin{pmatrix} -aw e^{-aw^2/2} \left( e^{-bz^2/2} - e^{-N/2}\right) _+ \\ -bz e^{-bz^2/2} \left( e^{-aw^2/2} - e^{-N/2}\right) _+ \end{pmatrix} {\varvec{1}}_{{{\,\textrm{supp}\,}}\Gamma _{M,N}}, \end{aligned} \end{aligned}$$

and hence,

$$\begin{aligned} \begin{aligned} \left|{\nabla \sqrt{\Gamma _{M,N}({{\varvec{x}}})}}\right|^2&= G_{M,N}^{-1} \biggl [ a^2 w^2 e^{-aw^2} \left( e^{-bz^2/2} - e^{-N/2}\right) _+^2\\&\quad + b^2 z^2 e^{-bz^2} \left( e^{-aw^2/2} - e^{-N/2}\right) _+^2 \biggr ] {\varvec{1}}_{aw^2\le N, bz^2\le N}, \end{aligned} \end{aligned}$$

and the kinetic energy of the truncated Gaussian ends up being

$$\begin{aligned} \begin{aligned} {{\,\textrm{KE}\,}}(\Gamma _{M,N})&= \frac{1}{2} G_{M,N}^{-1} \left( \int _{\{aw^2\le N\}} a^2 w^2 e^{-aw^2} \textrm{d}w\right) G_{b,N}\\&\quad + \frac{1}{2} G_{M,N}^{-1} \left( \int _{\{bz^2\le N\}} b^2 z^2 e^{-bz^2} \textrm{d}z\right) G_{a,N} \\&= \frac{1}{2} G_{a,N}^{-1} \sqrt{a} \left( \frac{\sqrt{\pi }}{2}{{\,\textrm{erf}\,}}(\sqrt{N}) - e^{-N}\sqrt{N}\right) \\&\quad + \frac{1}{2} G_{b,N}^{-1} \sqrt{b} \left( \frac{\sqrt{\pi }}{2}{{\,\textrm{erf}\,}}(\sqrt{N}) - e^{-N}\sqrt{N}\right) \\&= (G_{a,N}^{-1}\sqrt{a} + G_{b,N}^{-1}\sqrt{b}) \frac{\sqrt{\pi }}{4} \left( {{\,\textrm{erf}\,}}(\sqrt{N}) - \frac{2}{\sqrt{\pi }}e^{-N}\sqrt{N}\right) , \end{aligned} \end{aligned}$$

where to pass from the first to the second line we integrated by parts and changed variables. Recalling that $G_{\alpha ,\infty }^{-1}=\sqrt{\alpha }/\sqrt{\pi }$, for $N=\infty$, this gives

$$\begin{aligned} {{\,\textrm{KE}\,}}(\Gamma _{M,\infty }) = \left( \frac{\sqrt{a}}{\sqrt{\pi }}\sqrt{a} + \frac{\sqrt{b}}{\sqrt{\pi }}\sqrt{b}\right) \frac{\sqrt{\pi }}{4} = \frac{a+b}{4} = \frac{{{\,\textrm{tr}\,}}M}{4}, \end{aligned}$$

as already stated in (3.7). In order to compare the two energies, we estimate separately the difference of the terms involving *a* and *b*. Using the fact that, thanks to (7.2),

$$\begin{aligned} \frac{1}{G_{\alpha ,N}}-\frac{1}{G_{\alpha ,\infty }} = \frac{G_{\alpha ,\infty }-G_{\alpha ,N}}{G_{\alpha ,\infty }G_{\alpha ,N}} \le \frac{3e^{-N/2}}{G_{\alpha ,N}} \le \frac{3e^{-N/2}}{(1-3e^{-N/2})G_{\alpha ,\infty }} \le \frac{4e^{-N/2}}{G_{\alpha ,\infty }} , \end{aligned}$$

we can estimate that

$$\begin{aligned} \begin{aligned} \biggl |\frac{a}{4}&- G_{a,N}^{-1} \sqrt{a} \frac{\sqrt{\pi }}{4} \left( {{\,\textrm{erf}\,}}(\sqrt{N}) - \frac{2}{\sqrt{\pi }}e^{-N}\sqrt{N}\right) \biggr |\\&\le \left|{\frac{a}{4} - G_{a,\infty }^{-1} \sqrt{a} \frac{\sqrt{\pi }}{4} \left( {{\,\textrm{erf}\,}}(\sqrt{N}) - \frac{2}{\sqrt{\pi }}e^{-N}\sqrt{N}\right) }\right| \\&\quad + \left|{(G_{a,N}^{-1}-G_{a,\infty }^{-1}) \sqrt{a} \frac{\sqrt{\pi }}{4} \left( {{\,\textrm{erf}\,}}(\sqrt{N}) - \frac{2}{\sqrt{\pi }}e^{-N}\sqrt{N}\right) }\right| \\&\le \frac{a}{4} \left|{{{\,\textrm{erfc}\,}}(\sqrt{N}) + \frac{2}{\sqrt{\pi }}e^{-N}\sqrt{N}}\right| + 4 e^{-N/2} G_{a,\infty }^{-1} \sqrt{a} \frac{\sqrt{\pi }}{4}\\&\quad \times \left|{{{\,\textrm{erf}\,}}(\sqrt{N}) - \frac{2}{\sqrt{\pi }}e^{-N}\sqrt{N}}\right| \\&\le C \frac{a}{4} e^{-N/2}, \end{aligned} \end{aligned}$$

and similarly for *b*, and therefore

$$\begin{aligned} \begin{aligned} \left|{{{\,\textrm{KE}\,}}(\Gamma _{M,\infty }) - {{\,\textrm{KE}\,}}(\Gamma _{M,N})}\right|&\le C \frac{a+b}{4} e^{-N/2} = C {{\,\textrm{KE}\,}}(\Gamma _{M,\infty }) e^{-N/2}. \end{aligned} \end{aligned}$$

$$\square $$

## Footnotes

1 This is a standard fact about the ground state energy of an *n*-dimensional harmonic oscillator: in fact the ground state energy of the 1-dimensional harmonic oscillator Hamiltonian $h_i=-\varepsilon \partial _{x_i}^2+(a_ix_i)^2$ is $\sqrt{\varepsilon }a_i$ and the corresponding wave function is $\psi _i=e^{-2a_ix_2^2/(2\sqrt{\varepsilon })}$. After an orthonormal change of variables, any quadratic potential is equivalent to the *n*-dimensional Hamiltonian $\sum _i h_i$, which has ground state energy $\sqrt{\varepsilon }\sum _i a_i$ and ground state $\otimes _i \psi _i$.

2 When applying (3.8) to the integral of $\left|{\sqrt{\nabla ^2V({{\varvec{x}}})}{{\varvec{y}}}}\right|^2$ times the difference of the Gaussians, we use the fact that the matrix $\sqrt{\nabla ^2V({{\varvec{x}}})}$ is bounded from above by $\varepsilon ^{1/2}M_{\varepsilon ,\beta }(x)$.
